# A role for *BELLRINGER* in cell wall development is supported by loss-of-function phenotypes

**DOI:** 10.1186/1471-2229-12-212

**Published:** 2012-11-13

**Authors:** J Peter Etchells, Lucy Moore, Wen Zhi Jiang, Helen Prescott, Richard Capper, Nigel J Saunders, Anuj M Bhatt, Hugh G Dickinson

**Affiliations:** 1 Department of Plant Sciences, University of Oxford, South Parks Road, Oxford, UK; 2 Department of Pathology, University of Oxford, South Parks Road, Oxford, UK; 3 Faculty of Life Sciences, University of Manchester, Manchester, UK; 4 Present address: Department of Biochemistry, University of Nebraska, Lincoln, NE, USA; 5 Present address: Agilent Technologies, Wokingham, UK; 6 Present address: Centre for Systems and Synthetic Biology, Brunel University, London, UK

## Abstract

**Background:**

Homeodomain transcription factors play critical roles in metazoan development. *BELLRINGER* (*BLR*), one such transcription factor, is involved in diverse developmental processes in *Arabidopsis*, acting in vascular differentiation, phyllotaxy, flower and fruit development. *BLR* also has a redundant role in meristem maintenance. Cell wall remodelling underpins many of these processes, and *BLR* has recently been shown to regulate expression of *PECTIN METHYL-ESTERASE 5 (PME5)*, a cell wall modifying enzyme in control of phyllotaxy. We have further explored the role of *BLR* in plant development by analysing phenotypes and gene expression in a series of plants over-expressing *BLR*, and generating combinatorial mutants with *blr*, *brevipedicellus* (*bp),* a member of the *KNOX1* family of transcription factors that has previously been shown to interact with *blr*, and the homeodomain transcription factor *revoluta* (*rev*), required for radial patterning of the stem.

**Results:**

Plants over-expressing BLR exhibited a wide range of phenotypes. Some were defective in cell size and demonstrated misregulation of genes predominantly affecting cell wall development. Other lines with more extreme phenotypes failed to generate lateral organs, consistent with BLR repressing transcription in the shoot apex. Cell wall dynamics are also affected in *blr* mutant plants, and *BLR* has previously been shown to regulate vascular development in conjunction with *BP*. We found that when *bp* and *blr* were combined with *rev*, a set of defects was observed that were distinct from those of *bp blr* lines. In these triple mutants xylem development was most strikingly affected, resulting in an almost complete lack of vessels and xylem parenchyma with secondary thickening.

**Conclusions:**

Our data support a role for *BLR* in ordering the shoot apex and, in conjunction with *BP* and *REV,* playing a part in determining the composition and organisation of the vascular system. Microarray analysis strongly indicates that the striking vascular phenotypes of *blr bp rev* triple mutants and plants over-expressing *BLR* result from the misregulation of a suite of genes, targets of BLR in wild type plants, that determine cell size and structure in the developing vasculature.

## Background

*BELLRINGER* (*BLR*), also known as *VAAMANA*, *PENNYWISE, LARSON* and *REPLUMLESS,* is a member of the *BELL* family of homeodomain transcription factors and functions in diverse processes in the development of *Arabidopsis*[1-5]. *BLR* was identified independently as a suppresser of the floral homeotic gene *AGAMOUS* (*AG*) [5], as a factor required in fruit development for specification of the replum [1] and as a gene necessary for normal phyllotactic patterning [2,4]. BELL transcription factors were shown to enhance DNA binding of maize KNOTTED1 [6], the founding member of the *KNOX1* gene family [7], and *BLR* was subsequently shown to act synergistically with the *KNOX1* homeodomain transcription factor *BREVIPEDICELLUS* (*BP*) [3]. BLR and KNOX1 proteins heterodimerize [3] and, in *Nicotiana* epidermal cell transient assays, BLR translocates to the nucleus on binding to KNOX1 proteins [2]. This event is likely to have evolved early in eukaryotic development as it is required for the haploid-diploid transition in the green algae *Chlamydomonas*[8].

Although the expression patterns of *BLR* and *KNOX1* overlap, they are not identical [3] and *blr* and *knox1* mutants show distinctive phenotypes, suggesting that BLR function is unlikely to be confined to acting with KNOX1 proteins alone. Nevertheless, similarities are apparent, such as the short stature of both *blr* and *bp* plants [2-4] and the ability of *blr* and *bp* to enhance weak alleles of *shoot meristemless* (*stm*) [2,4], a *KNOX1* gene required for shoot apical meristem maintenance [9,10], which points to a redundant role for *BLR* and *BP* in stem cell fate establishment [2-4]. Reports of genetic interactions between *blr* and *bp* mutants have, however, been conflicting, with the short stature phenotype of the *bp blr* double mutation being variously interpreted as additive [4], synergistic [3], or novel - because inflorescence elongation is observed in the single mutants but not in the double [2]. Phenotypic differences between *blr* and *bp* mutants are unlikely to result from redundancy between family members because multiple knockout lines within the *KNOX1* and *BELL* families exhibit diverse phenotypes. For example, vegetative to reproductive transition defects are observed when *blr* mutants are combined with mutations in *poundfoolish* (*pnf*), the gene with which *BLR* shares most similarity in *Arabidopsis*[11], which are not observed in multiple *knox1* mutants [12,13]. Similarly, multiple *knox1* mutants do not resemble *blr* plants as one role of *BP* is repression of *KNAT2* and *KNAT6*[12]. *BLR* is thus likely to have functions both dependent on and independent of *KNOX1*.

One *KNOX1* independent function of *BLR* may include control of phyllotaxy as similar defects are not present in *knox1* mutants. Normal phyllotaxy requires correct patterning of cell divisions [14,15] which depend in turn on strictly regulated cell-wall remodelling events [16] themselves driven by mechanical constraints [17]. Primordia outgrowth is accompanied by the de-methyl-esterification of pectic polysaccharides in the cell wall, and perturbation of the methyl-esterification status of pectins within the meristem by altering expression levels of *PECTIN METHYL-ESTERASE 5* has been shown to alter phyllotactic pattern [18]. *PME5* has been demonstrated to be negatively regulated by *BLR* in the meristem dome and this interaction is essential for maintenance of phyllotaxy, as incorrect positioning of organ initiation in *blr* mutants is suppressed in *blr pme5* double mutants. The relationship between *BLR* and *PME5* appears to be context specific as *BLR* promotes *PME5* expression in internodes [19].

The functions of *BP* and *BLR* do overlap in vascular development. Typically in *Arabidopsis* stems, vascular tissue is radially arranged in collateral bundles with xylem present towards the centre of the stem and phloem positioned towards the outside, such that the xylem, vascular meristem (procambium) and phloem are positioned along the mediolateral axis [20]. Xylem is characterised by the presence of large vessels for water transport and smaller xylary fibres, both of which have secondary cell walls with the adjacent procambium forming an arc of dividing cells. Within the stem, the vascular tissue represents a developmental series with most recently derived tissue at the top and the oldest at the base [20]. A continuous vascular ring has been described in *blr* lines, a phenotype enhanced in *bp blr* double mutants [3] by the appearance of undeveloped vascular bundles [3] containing cells previously only observed occurring in small, isolated strips overlying some vascular bundles of *bp* plants [21,22].

Here we have exploited transgenic plants over-expressing *BLR* as a tool to identify putative downstream targets of this transcription factor. In addition to the previously described target of *BLR*, *PME5*[19], our microarray analysis has identified misregulation of many other genes encoding cell-wall-associated proteins following *BLR* over-expression. Defects in cell wall metabolism can affect cell size [23] and we therefore examined *blr* mutant vascular tissue, where cell size defects have previously been described [3]. We uncovered a genetic interaction between *blr*, *bp* and *revoluta* (*rev*), a homeodomain transcription factor of the HD-ZipIII family that has previously been shown to specify adaxial identity, radial patterning in the stem [24] and xylem differentiation [25]. Our results add further support to the notion [19] that in addition to regulating genes at the shoot apex, *BLR* plays a wider role in development through its control of the extracellular matrix.

## Results and discussion

### Over-expression of *BELLRINGER* reduces organ size

**Figure 1:**
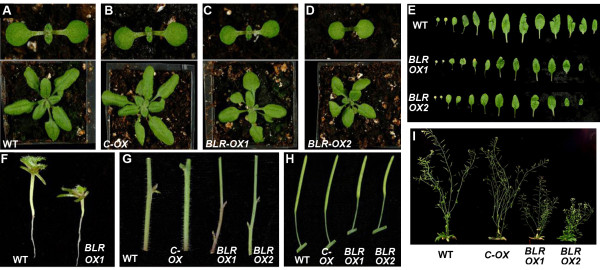
**Phenotype of *BLR-OX *lines. **7 day-old (top panel) and 21 day-old (lower panel) wild type (**A**), transformed control (**B**), *BLR-OX1* (**C**) and *BLR-OX2* (**D**). (**E**) Dissected rosette leaves of 4 week-old WT, *BLR-OX1* and *BLR-OX2* plants. (**F**) WT and *BLR-OX1* hypocotyls at 2 weeks-old. (**G**-**I**) Comparison of 5 week-old *BLR-OX1*, *BLR-OX2* and control plant inflorescence stems (**G**), mature siliques (**H**) and stature (**I**).

**Figure 2:**
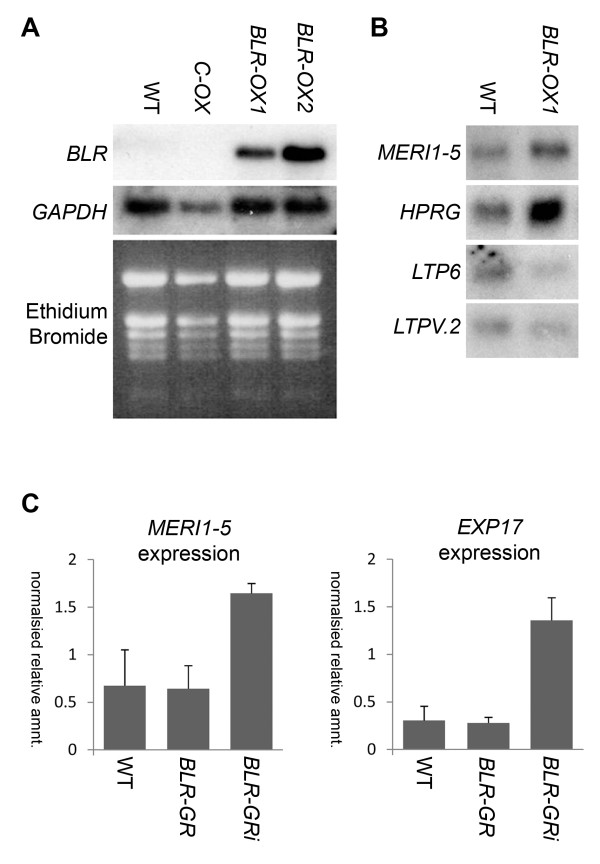
**Gene expression changes in plants over expressing *BLR. ***(**A**) Comparison of *BLR* and *GAPDH* expression in wild type (WT), transformed control (C-OX), *BLR-OX1* and *BLR-OX2* lines by Northern blot. (**B**) Comparison of At4g30270/*MERI1-5*, At5g65660/*HPRG*, At3g08770/*LTP6* and At3g53980/*LTPV.2* expression by Northern blot. (**C**) Expression of *MERI-5*, and *EXP17* in wild type, *BLR-GR* and *BLR-GR*^*i*^ (72 hour induction) determined by qRT-PCR and shown as relative amount normalised to expression of *ACT2.*

Over-expression studies have been informative in functional analysis of *KNOX1*[26-28]. Using a similar strategy, *BLR* cDNA was used to generate *35S::BLR* constructs from which *BLR* over-expression (*BLR-OX*) transgenic lines were generated. Plants were observed with short hypocotyls, small rosette leaves, narrow cauline leaves, and short siliques (Figure 1A1F, 1H). *BLR-OX* plants also had shorter, thinner stems than wild type with more axiliary branches (Figure 1G, 1I). Northern blot analysis showed eight lines to exhibit increased *BLR* levels; *BLR* transcript levels in two of these lines, *BLR-OX1* and *BLR-OX2,* are shown in Figure 2A.

**Figure 3:**
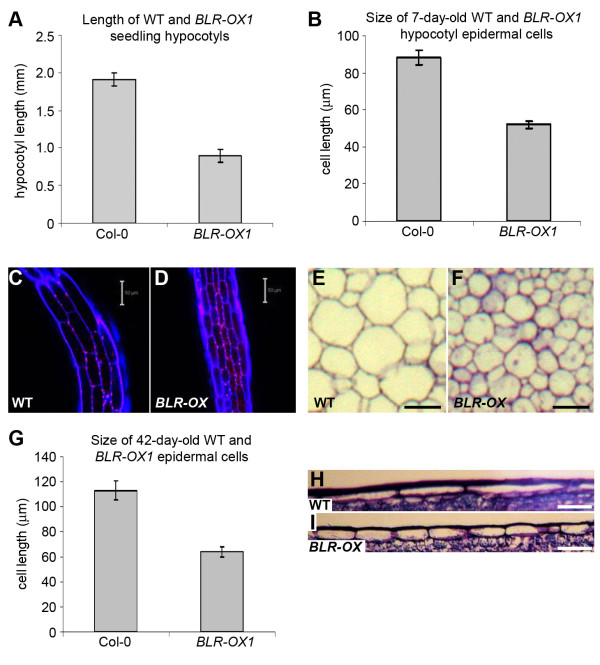
**Cell size defects of *BLR-OX *seedlings. **(**A**) Average hypocotyl length of 7 day-old wild type and *BLR-OX1* lines (*n* = 14). (**B**) Mean epidermal cell size in hypocotyls of 7 day-old plants (*n* = 33). 7 day-old wild type (**C**) and *BLR-OX* (**D**) hypocotyl epidermis stained with propidium iodide. 200 μM^2^ cross-section from 42 day-old wild type (**E**) and *BLR-OX1* (**F**) stems showing parenchyma cells. (**G**) Mean epidermal cell size of 42 day-old plants (*n* = 41). 100 μM row of epidermal cells from 42 day-old wild type (**H**) and *BLR-OX* (**I**) stems. Error bars show standard error. Scales are 50 μm (E-F), or 10 μm (H-I).

The organs of *BLR-OX* lines were smaller than wild type, so differences in cell size and number were investigated in 7 day epidermal hypocotyl cells from *BLR-OX* and wild type plants (Figure 3A-D). *BLR-OX* epidermal cells were significantly shorter than controls (Figure 3B); similar reductions were observed in longitudinal sections of inflorescence stem epidermis (Figure 3G-I) and transverse sections of pith parenchyma (Figure 3E-F). This reduction in cell size is thus likely to contribute to the smaller organ size of *BLR-OX* plants. Other histological features, including those in vascular tissue were indistinguishable from wild type.

### Expression changes in *BLR-OX* plants

To explore the changes in transcription underlying the *BLR-OX* phenotype, transcripts from hypocotyls of *BLR-OX* and control plants were subject to microarray analysis at stage 1.01 [29] of development. Using the ‘Significance Analysis of Microarrays’ strategy [30], 3109 genes demonstrated consistent expression changes and acceptable spot confidence of which 2957 had appropriate q-values (<0.1%). 927 genes demonstrated >1.5 fold expression change (Additional file 1: Table S1). Three lines of evidence point to the microarray data reflecting gene expression changes *in planta*; first, *BLR* was up-regulated 3.6 fold in *BLR-OX* compared to Col-0 (Table 1). Second, the expression of *PME5*, previously shown to be negatively regulated by *BLR* is reduced 2.3 fold (Table 1) and third, the promoters of 70 of the 100 genes showing the greatest fold change carried predicted BLR consensus DNA binding sites (Table 1). In contrast, the DNA binding sequence for ATHB2, which in common with BLR has a 9 base pair consensus sequence, occurs in only 9 of the 100 promoter regions. The array data was validated by testing expression of *MERI-5* (At4g30270; 4.3 fold increase in *BLR-OX*), a xyloglucan endotransglucosylase which acts to hydrolyze glycosyl bonds, *HPRG* (At5g65660; 1.6 fold increase), a hydroxyproline-rich plasma membrane localised glycoprotein, and *LPT6* (At3g08770; 1.9 fold decrease) and *LTPV.2* (At3g53980; 6.3 fold decrease), both of which encode endomembrane localised lipid binding/transport proteins, by northern blot (Figure 2B). In all instances expression reflected the array data.

**Table 1:** **Genes prominently mis-regulated in *BLR-OX***

**AGI number**	**Common name**	**Expression**		
		***BLR-OX***	**WT**	**Fold Change**
**UPREGULATED IN BLR-OX**				
At1g10070 ^1,4^	Branched-chain amino acid aminotransferase 2 (BCAT-2)	469 ± 320	47 ± 12	**10.0**
At4g30270 ^1,2,4^	Xyloglucan endotransglucosylase/hydrolase 24 (MERI-5)	2269 ± 601	522 ± 38	**4.3**
At3g47340 ^1,2,4^	Glutamine dependent aparagine synthetase 1	1,231 ± 470	281 ± 41	**4.4**
At1g75750 ^1,2,4^	GA-responsive GAST1 protein homolog (GASA1)	10890 ± 4769	2641 ± 390	**4.1**
At5g02030 ^1,4^	BELLRINGER	2507 ± 452	706 ± 1,23	**3.6**
At2g19800 ^1,4^	Myo-inositol oxygenase 2 (MIOX2)	2104 ± 759	700 ± 357	**3.0**
At5g56870 ^1,4^	Beta-galactosidase 4 (BGAL4)	842 ± 140	265 ± 57	**3.2**
At2g30600 ^2,4^	BTB/POZ domain protein involved in cell adhesion	2624 ± 839	1110 ± 353	**2.4**
At5g49360 ^1,4^	β-D-xylosidase/α-L-arabinofuranosidase (BXL1)	2105 ± 591	983 ± 484	**2.1**
At4g35770 ^2^	Senescence-associated/Dark inducible protein (SEN1/DIN1)	2393 ± 413	1,206 ± 374	**2.0**
At2g45170 ^2,4^	Autophagy 8e (APG8e)	3685 ± 133	2007 ± 193	**1.8**
At4g36850 ^2,4^	PQ-loop repeat family/transmembrane family protein	503 ± 88	270 ± 45	**1.9**
At5g07440 ^2,4^	Glutamate dehydrogenase 2 (GDH2)	4403 ± 685	2364 ± 237	**1.9**
At4g01630 ^1,4^	Expansin, putative (EXP17)	162 ± 24	88 ± 17	**1.8**
At5g11720 ^1^	Glycoside hydrolase family 31	1054 ± 113	578 ± 85	**1.8**
At1g53910 ^1,4^	AP2 domain-containing protein RAP2.12	9167 ± 47	5043 ± 441	**1.8**
At1g12780 ^1,2^	UDP-D-glucose/UDP-D-galactose 4-epimerase 1 (UGE1)	239 ± 43	136 ± 35	**1.8**
At4g14130 ^1^	xyloglucan endotransglycosylase-related protein (XTR7)	41,2 ± 156	272 ± 105	**1.5**
At1g35250 ^1,4^	Thioesterase superfamily protein	1164 ± 132	769 ± 91	**1.5**
**DOWNREGULATED IN BLR-OX**				
At3g53980 ^1,4^	Protease inhibitor/seed storage/lipid transfer protein family (LTPV.2)	282 ± 35	1776 ± 616	**6.3**
At1g73620 ^1^	Thaumatin-like protein, putative	268 ± 23	741 ± 23	**2.8**
At1g21890 ^1,4^	Nodulin MtN21/EamA-like transporter family protein	165 ± 51	403 ± 81	**2.4**
At5g06390 ^1,4^	FASCICLIN-like arabinogalactan protein 17 (FLA17)	65 ± 11	161 ± 6	**2.5**
At5g67400 ^1,4^	Root hair specific 19/Peroxidase 73 (RHS19/PER73)	153 ± 8	377 ± 37	**2.5**
At5g47500 ^1,4^	Pectin lyase-like superfamily protein (PME5)	302 ± 93	687 ± 136	**2.3**
At3g05600 ^1,4^	α/β-Hydrolase superfamily protein	58 ± 1,2	116 ± 13	**2.0**
At1g62560 ^1,3^	Flavin-monooxygenase glucosinylate S-oxygenase 3 (FMO GS-OX3)	406 ± 102	775 ± 194	**1.9**
At3g08770 ^1,4^	Lipid transfer protein 6 (LTP6)	1475 ± 1,25	2758 ± 98	**1.9**
At3g05470 ^1,4^	Actin-binding FH2 (formin homology 2) family protein	275 ± 160	516 ± 311	**1.9**
At5g01870 ^1^	Lipid transfer protein 10 (LTP10)	360 ± 24	638 ± 58	**1.8**
At5g61020 ^1,4^	evolutionarily conserved C-terminal region 3 (ECT3)	421 ± 98	736 ± 160	**1.7**
At1g20930 ^1,4^	Cyclin-dependent kinase B2;2 (CDKB2;2)	359 ± 61	598 ± 66	**1.7**

Genes with putative roles in extracellular matrix regulation (GO analysis), altered expression in *bp* mutants [36] or having a BLR consensus binding site in the promoter ( http://arabidopsis.med.ohio-state.edu/AtcisDB/) are shown.

^1^Role, or putative role in modification of the extracellular matrix.

^2^Reduced expression in *bp* compared to wild-type plants (Mele *et al*., 2003).

^3^Increased expression in *bp* compared to wild-type plants (Mele *et al*., 2003).

^4^BLR DNA binding consensus sequence present in promoter.

Expression values are presented as the mean ± standard error for three biological replicates.

To understand better the significance of transcriptional differences between *BLR-OX* and control plants, a gene ontogeny (GO) analysis was carried out. Strikingly, the only significant change proved to be in cell wall associated gene expression, which was enriched from 1.3% in controls to 6.8% in *BLR-OX* lines (Table 1) pointing to a role for BLR in regulating cell wall metabolism or organisation. Changes in cell size are regularly accompanied by expression changes in cell wall associated genes and the GO analysis thus draws together the gain-of-function phenotypes (Figures 1, 3) and the array data (Table 1). Lipid transfer proteins (LTPs) [31] and peroxidases [32] loosen cell walls and induce cell expansion; thus a 6 fold decrease in expression of *LTP* and a 2.5 fold reduction in peroxidise expression in *BLR-OX* plants (Table 1) is consistent with the reduction in cell size observed in *BLR-OX* lines. *PME5,* which is negatively regulated by *BLR* (2.3 fold down-regulated in *BLR-OX*), alters the gelling properties of cell walls [33], while other genes with altered expression are implicated by homology to have roles in cell expansion. For example members of the thaumatin family (2.8 fold down-regulated) have potential endo-β-1,3 glucanase activity [34].

### Extreme *BLR* gain-of-function plants lack organ initiation

Organised phyllotaxy, lacking in *blr* mutants [2-4] requires accurate cell expansion and division during organogenesis [15], processes reliant on close regulation of cell-wall formation and expansion [16,18]. We therefore anticipated that phenotypes observed in *BLR-OX* lines could be strengthened to include disruption of organ initiation due to altered cell wall metabolism, particularly as *BLR-OX* lines analysed above exhibited only a relatively small expression increase (3.6 fold). Furthermore, in some contexts, BLR is localised in the cytoplasm, and its nuclear localisation is dependent on an interacting partner [2,35]. Plants were therefore generated expressing a steroid-inducible version of *BLR* in the form of a *BLR-GR* fusion regulated by the *35S CaMV* promoter which would result in nuclear localisation of BLR in a steroid inducible manner, showing independence of an interacting partner. To confirm that expression changes of cell wall modifying enzymes following *BLR-GR* induction were consistent with those observed in *BLR-OX* lines, plants were subjected to dexamethasone (dex) induction for 72 h, as maximum activity of direct transcriptional targets in this system has been demonstrated as occurring between 64 and 128 hours [36]. Following dex induction, expression of both *MERI-5* and *EXP17* was elevated (Figure 2C), as in *BLR-OX* lines (Table 1) providing further evidence for *BLR* regulation of the extracellular matrix.

**Figure 4:**
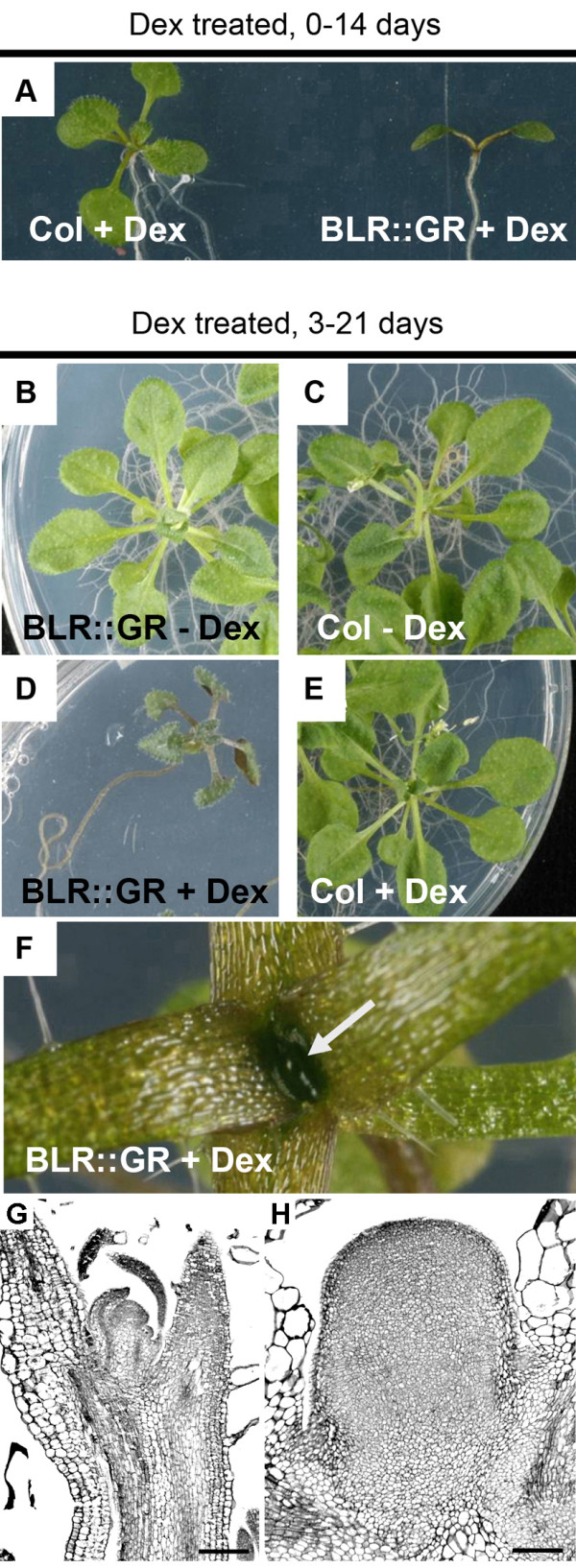
**Phenotypes of *35S::BLR-GR *Plants. **(**A**) Plants expressing *BLR-GR* did not generate post-embryonic structures when germinated on plates containing Dex. (**D**-**H**) Plants treated with dex, 3 days post-germination. *BLR-GR*^*i*^ (**D**, **F**) did not initiate new lateral organs but developed a bulbous structure (arrow in **F**) at the apex, whereas dex treated wild type (**E**) and untreated *BLR-GR* (**B**) or wild type (**C**) developed normally. Longitudinal sections through apices (**G-H**) shows that in wild type plants at 3 weeks-old, the programme of inflorescence development had initiated (**G**), but induced *35S::BLR::GR* plants lateral organ initiation had ceased (**H**), Scales are 100 μm.

When *BLR-GR* seeds were germinated on 10 μm dex, only cotyledons were visible after 14 days (Figure 4A), suggesting that high levels of BLR in the nucleus lead to extremely limited growth. This observation may explain the relatively low increases in *BLR* expression in *BLR-OX* as plants with higher expression levels may not have been recovered in the T1 generation. Seedlings germinated for 3 days in the absence of steroid and subsequently transferred to 10 μm dex (*BLR*–*GR*^*i*^), produced leaves with severely reduced expansion (Figure 4B-E). Visible leaves no longer emerged after 21 days and the apex became enlarged (Figure 4F), suggesting that growth continued in the absence of organ initiation. Sections cut through these enlarging *BLR*–*GR*^*i*^ apices revealed an absence of lateral organ primordia or any other clearly differentiated cell types (Figure 4G-H), suggesting that high levels of nuclear BLR results in anisotropic growth.

Normal organ initiation and phyllotaxy in *Arabidopsis* requires interplay between *BLR* repression of *PME5* in the shoot apical meristem [19], and loss of this repression during formation of organ primordia [4]. Loss of *PME5* repression has been proposed to allow other cell wall modifying components to access the wall. Our results support this hypothesis for, in our *BLR-GR*^*i*^ plants, *BLR* would continue to negatively regulate *PME5* with the consequent inhibition of organ initiation we observed.

Organ initiation requires both the correct patterning of cell divisions [14,15] and strictly regulated cell wall remodelling [16,18]. The organisation of cell divisions in the apex has been shown to depend on mechanical pressures [17], which are likely to be influenced by the cell wall [37]. The current model for cell-wall remodelling during organ initiation at the apex holds that the breakage and annealing of cell-wall components is controlled by connections with microtubules, possibly through regulation of cellulose-microfibril length [38]. Treatment of *Arabidopsis* apices with oryzalin, which depolymerises microtubules and prevents ordered division, results in a bulbous apex and loss of lateral-organ initiation when combined with disrupted auxin signalling [39]. The cell wall plays a central part in the transmission of chemical and mechanical signals between cells, and the positioning of developmental cues at the apex of sunflowers is reported to be sensitive to mechanical interventions [17]. Our data suggest that *BLR* modulates the expression of genes encoding cell wall associated proteins, and our observation that plants strongly over-expressing *BLR-GR*^*i*^ fusions generate bulbous apices incapable of forming lateral organs is consistent with the view that closely-regulated cell wall development is essential for ordered initiation of lateral organs at the shoot apex.

### *rev* enhances the *bp blr* phenotype

The array experiments identified genes involved in cell expansion to be misregulated in *BLR-OX* plants. Expression changes in genes involved in cell expansion are also a feature of ectopic *knox1* (*STM*) expression in poplar stem vascular tissue [40], and several putative *BLR* targets identified in *BLR-OX* arrays are reciprocally regulated in *bp* mutants [41] (Table 1). *BP* is required for patterning the inflorescence stem as sectors of differentiation-defective epidermal, cortical and vascular cells are present in *bp* mutant stems [22,42,43]. Furthermore, *BP* negatively regulates biosynthesis of lignin [41], a cell wall polymer, that is present in cells that have secondary walls including those of the vascular tissue. Despite exhaustive analysis, *blr* and wild type hypocotyls proved indistinguishable (not shown); however we saw clear differences in the vascular tissue of the inflorescence stem where *BLR* is expressed [4]. Loss of function *blr* phenotypes have been described in this region [3] in the relatively newly formed vascular tissue at the top of the stem (between the third and fourth siliques). Here *blr* mutants were found to form a continuous ring of vascular tissue, while *bp blr* double mutants demonstrated an enhanced *blr* phenotype as cells in the continuous vascular ring failed to differentiate and contained small, densely stained cells where xylem cells with secondary cell walls formed in wild type plants [3].

**Figure 5:**
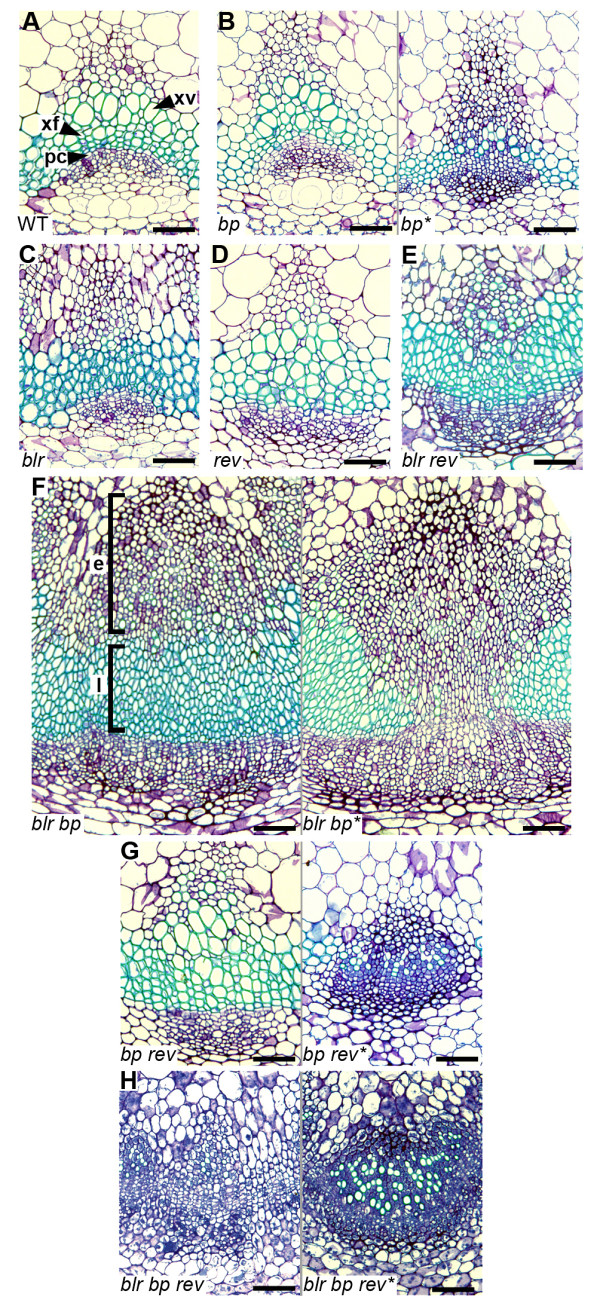
**Vascular tissue in *blr*, *bp *and *rev *mutant combinations.** Toluidine blue stained sections from inflorescence stem vascular tissue adjacent to the first cauline leaf. (**A**) Wild type vascular bundle with arrowheads pointing to a xylem vessel (xv), xylary fibres (xf) and vascular meristem (procambium; pc). (**B**) *bp* mutants are indistinguishable from wild type, except in differentiation defective stripes (bp*) where the arc of procambium cells is flattened, xylem vessels are absent or reduced in size and many cells are small and darkly stained suggesting they lack secondary walls (green in wild type xylem; **A**). *blr* (**C**) and *blr rev* (**E**) vascular tissue in which the arc of procambium is flattened and xylem vessels appear absent. (**D**) *rev*. (**F**) Large vascular expansion in *blr bp*. In vascular tissue derived early in development (square bracket; e) all cells are small and many lack secondary walls. In contrast, later derived tissue (square bracket; l) contains differentiated xyary fibres but no vessels, except in differentiation defective regions of the stem (blr bp*) where very few cells with secondary wall are present. (**G**) *bp rev* double mutants are indistinguishable from *rev* in most regions of the stem, however, in differentiation defective regions (bp rev*) cells were smaller than those observed in bp*. (**H**) Cells in *blr bp rev* inflorescence stems were extremely small and in the vast majority of cases secondary walls appeared absent. Scales are 50 μm.

**Figure 6:**
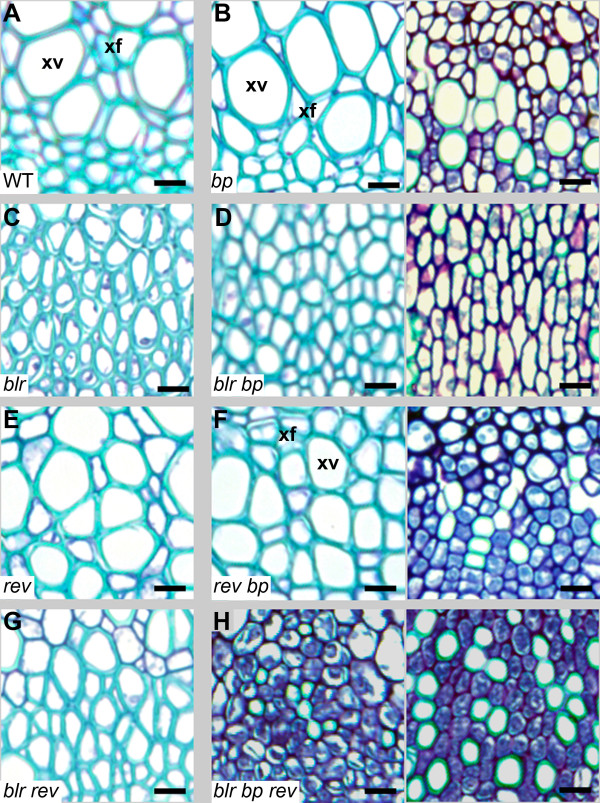
***blr*, *bp *and *rev *mutant xylem.** Xylem from vascular bundle in inflorescence stems. (**A**) Wild type xylem with vessels (xv) and xylary fibres (xf) marked. (**B**) *bp* mutant xylem is indistinguishable from wild type (left hand side; LHS), except in differentiation defective tissue (right hand side; RHS) where vessels are absent or reduced in size and many cells lack secondary walls (green in wild type xylem; **A**). (**C**) Extreme *blr* phenotype where xylem vessels appear absent. (**D**) *blr bp* xylem in later derived tissue (see Figure 5F) with xyary fibres but no vessels (LHS). In differentiation defective regions of the stem (RHS) Few cells with secondary wall are present. (**E**) *rev* xylem, similar to wild type. (**F**) *bp rev* xylem is like that of *rev* (LHS) except in differentiation defective regions (RHS) where cells were smaller than those observed in *bp*. (**G**) *rev blr* xylem. (**H**) Most *blr bp rev* xylem lacked secondary walls. Scales are 10 μm.

We analysed vascular tissue at a later stage in development, adjacent to the first cauline leaf on the primary inflorescence stem. *blr* vascular bundles were patterned differently from those of wild type, as the arc of procambial cells present in wild type appeared shallower in *blr* (Figure 5A, C). *blr* mutants with the strongest phenotypes also had vascular bundles lacking large xylem vessels and containing apparently only xylary fibres, suggesting that there was either a failure of vessel specification, or a defect in vessel cell size (Figure 6A, C). ‘Stripes’ of differentiation-defective cells overlying some vascular bundles in *bp* mutants gave rise to two distinct vascular morphologies (Figures 5B, 6B); Within the stripes xylem differentiation was altered because as with *blr* mutants, xylem lacked large vessels, however, in contrast to *blr* mutants, many of the smaller cells had not generated secondary cell walls [22,42] (Figure 6B). In areas outside the differentiation defective stripe, vascular patterning was indistinguishable from wild type (Figure 5B).

*bp blr* vascular tissue has been described as reminiscent of that present in differentiation defective areas of *bp* mutants [3]. In *bp blr* bundles outside the differentiation-defective stripe, small darkly staining cells as described by Smith *et al.*[3], were observed towards the centre of the stem (Figure 5F) suggesting that these cells were derived early in vascular development. However, xylem cells generated later in development (closest to the procambium) underwent significant differentiation in *bp blr* double mutants because secondary cell walls were present, but as with extreme *blr* mutants, all xylem cells were reminiscent of xylary fibres (Figure 6D). In contrast, we observed very few xylem cells with secondary walls in *bp blr* vascular bundles within the differentiation-defective tissue associated with the *bp* mutation, even at this late stage of development (Figures 5F, 6D). Consequently, and consistent with other reports [3], *blr* enhances *bp* in the context of the vascular tissue and this interaction is particularly evident in mature *bp blr* vascular tissue within differentiation-defective regions of the stem (Figures 5A-C, 5F, 6A-D). We reasoned that analysis of *bp* and *blr* mutant combinations in a background of altered xylem identity may reveal redundant functions of *bp* and *blr*. *REV* has been associated with radial patterning in the stem [24] and has previously been shown to be required for development of interfascicular fibres [25,44], for xylem differentiation and maintenance of xylem cell number [25]. The role of *REV* in the xylem is likely to be partially redundant as phenotypes appear enhanced in *rev phb*/+ or *rev phv* mutants [45]. Furthermore in the shoot apex, as with *bp*[13] and *blr*[2], *rev* enhances the phenotype of weak alleles of *stm*[46] suggesting that *REV* may have overlapping functions with *BLR*. We therefore examined inflorescence stem vascular tissue in *bp*, *blr* and *rev* combinatorial mutants. Within *rev* vascular bundles xylem tissue resembled wild type in that both vessels and parenchyma were present, however the arc of procambium was shallower as in *blr* plants (Figure 5D), a phenotype also occurring in *bp rev* mutant vascular tissue, outside of differentiation defective tissue (Figure 5G). Within differentiation defective tissue, the xylem cells were generally similar to those of *bp* single mutants except that the relatively few cells that developed secondary walls were extremely small compared with those of *bp* (Figure 6F). *blr rev* and *blr* vascular bundles were similar but, strikingly, the xylem in vascular bundles of *blr bp rev* triple mutants comprised small and darkly stained xylem cells, whether or not the bundles lay within the stripe of differentiation-defective cells (Figures 5H, 6G). In extreme cases, no cells with secondary walls were seen in vascular bundles (Figure 5H).

Genetic interactions between *BLR*, *REV* and *BP* in the stem vary depending on tissue type. Vascular cell division between vascular bundles is one of the hallmarks of secondary growth (radial expansion). *BP* and *BLR* repress this process as *bp blr* mutants have a continuous ring of vascular tissue [3]. However, *REV* appears to be required for the large increase in vascular tissue present in *bp blr* because *bp blr rev* lines demonstrate a reduction in vascular tissue compared to *bp blr* (Figure 5F, H). This observation supports a role for *REV* (in contrast to *BP* and *BLR*) in promoting secondary growth, a function that has previously been suggested for its poplar orthologue [47]. *BLR*, *REV* and *BP* do not have contrasting roles in regulating xylem differentiation. The presence of xylem cells with secondary walls was reduced in double and triple mutant combinations (Figure 6) suggesting that *BLR*, *REV* and *BP* act together in this process. Consequently, *BLR*, *REV* and *BP* interactions are context dependent.

Although the primary defect observed in *BLR-OX* was one of reduction in cell size, similar to that observed in vascular tissue of *blr* mutant combinations, the relationship between cell size and expression of particular cell wall modifying genes is not always straightforward [48-53]. For example, over-expression of an E2Ff transcription factor causes a reduction in cell size, accompanied by a decrease in expression of expansins (*EXP3, 7* and *9*), a UDP-glucose-glucosyl transferase (*UGT*) and *MERI-5*[52] (up-regulated 4.6 fold in *BLR-OX*). However, over-expression of *MERI-5* is reported elsewhere as promoting precocious elongation [54]. *BLR* control of the extracellular matrix is thus likely to be similarly complex. *BLR-OX* plants have smaller cells than those of wild-type, but *blr* mutants, when combined with *bp* and *rev* also resulted in vascular tissue with smaller cells.

## Conclusions

Control of cell expansion is essential for normal plant development. Our results demonstrate that *BLR* controls cell expansion and differentiation in vascular development in a process also involving *BP* and *REV* transcription factors. Microarray analysis of plants over-expressing *BLR* indicates that *BLR* targets many genes involved in cell wall regulation. As dynamic control of the cell wall is essential for cell expansion, we suggest that the striking phenotypes of plants over- and under-expressing *BLR* may result from the disruption of this process.

Closely regulated cell wall dynamics are also required for a normal pattern of organ initiation at the shoot apex and this process has previously been shown to be regulated by *BLR* repression of *PME5*[19]. Our experiments with *BLR-GR* suggest that further transcriptional targets of *BLR* may also play important parts in this process.

## Methods

### *BLR* overexpression

Full-length *BLR* cDNA was cloned into pGEM-T Easy (Promega), subcloned as an *Eco*RI fragment into pART7 [55] and sequenced to identify plasmids with *BLR* in the sense orientation. The resultant *CaMV35S*::*BLR*::*OCS*-terminator cassette was subsequently excised from pART7 using *Not*I and subcloned into pMLBART. The resultant *BLR-OX* binary vector was introduced into *Arabidopsis* (Col-0) using standard methods [56]. For steroid-inducible *BLR* lines, two PCR products were amplified, one containing *BLR* cDNA and a second containing the glutocoticoid receptor (GR). The GR forward primer (AAAAAGCAGGCTTAATGACCACACTCAACATG) contained a partial attB1 site, while the reverse primer (GCTCCACCTCCACCTCCATACTCATGG) contained an Ala–Gly linker and part of the 5′ *BLR* sequence. Similarly, the forward *BLR* primer (GGAGGTGGAGGTGGAGCATGGCTGATGCAT) contained the Ala–Gly linker while the reverse primer (AGAAAGCTGGGTTCAACCTACAAAATCATG) contained an attB2 site. The two PCR products were mixed in equimolar concentrations and used as templates for overlap PCR with attB1 and attB2 primers resulting in a *BLR-GR* in-frame fusion, which was cloned into pDONR Zeo, sequenced, and subcloned into pEarleyGate100 using Gateway technology. BASTA-resistant Col-0 transgenic lines were generated as described above, and *Arabidopsis* transformants that had a wild-type phenotype when uninduced but responded to 10-μM dexamethasone were used for subsequent analysis.

### Gene expression analysis

For Microarray experiments total RNA was extracted from Col-0 and *BLR-OX* plants using an RNeasy Plant Mini Kit (Qiagen). RNA integrity was checked on a 2100 BioAnalyzer (Agilent, CA, USA) prior to cDNA synthesis from 2 μg total RNA using a 3DNA Array 900^TM^ kit (Genisphere Inc., Hatfield, PA, USA) with RT-primers that contained a dendrimer [57] capture sequence. Labelled cDNAs were hybridised for 16 hours to the *Arabidopsis* Atv3.3.2.z Array printed with the Operon *Arabidopsis thaliana* version 3 Array-Ready Oligo Set (Galbraith, USA) using a Slidebooster (Advalytix, Brunnthal, Germany) with a power setting of 27 and a pulse setting of 7:3. Array slides were washed in 2× SSC, 0.2% SDS (10 min, 55°C), 2× SSC (10 min, Room temp.) and 0.2× SSC (10 min, room temp.). Fluorescent-labelled Genisphere 3DNA dendrimers containing approximately 850 Cy3 or Cy5 dyes were hybridized for 4 hours to the arrays. Following a set of washes as above, arrays were scanned using a ScanArray Express HT Microarray Scanner (Perkin Elmer).

After calibration to obtain scans within the optimum dynamic range, image analysis and spot feature quantification were performed using BlueFuse version 3.1 (BlueGnome, Cambridge, UK). Data from each slide were confirmed to be essentially linear and globally median-normalized before further analysis. Spots were excluded when the combined confidence values of biological replicates was <0.005. Microarray data were median-normalized in Cy5 and Cy3 channels. Spots that showed expression changes in the same direction in all three replicates were analysed further.

Normalized Cy5/Cy3 log_2_ ratios (three values for each gene for *BLR-OX* lines and three for Col-0) were used to perform a significance analysis of microarray (SAM) [30] using the TIGR Multi Experiment Viewer (TMEV V3.0.3). The imputation engine was set as 10-nearest neighbour imputer and the number of permutations was 100. The delta value in the Significance Analysis was adjusted so that estimated false discovery rate (FDR) was 0% for significant genes. The expression-fold change was set as 1.5 as a threshold for changes of potential biological importance. The microarray data described here have been submitted to MIAMExpress ( http://www.ebi.ac.uk/arrayexpress/experiments/E-MEXP-852). GO annotation analysis was performed using http://www.arabidopsis.org/tools/bulk/go/index.jsp.

For northern blot analysis of primary transformants, two micrograms of total RNA extracted from leaf tissue was subjected to electrophoresis on a 1.2% formaldehyde agarose gel, which was subsequently blotted onto Hybond-NX membrane and hybridised with a full-length ^32^PdCTP-labelled cDNA probe. RNA was also visualised by ethidium bromide staining and the filter hybridised with *GAPDH* as loading controls. Validation of microarray targets was carried out on RNA from 6–7 day-old seedlings. Primers were used to amplify the 3^′^ UTR and adjoining exons for specific probes, which were ^32^PdCTP-labelled.

qRT-PCR analysis was carried out using SYBR Green JumpStart Taq ReadyMix (Sigma) and an ABI Prism 7000 machine (Applied Biosystems) with the standard sybr green detection programme. A melting curve was produced at the end of every experiment to ensure that only single products were formed. Gene expression was determined using a version of the comparative threshold cycle (Ct) method. The average amplification efficiency of each target was determined using LinReg [58] and samples were normalised to *18S rRNA* (not shown) and *ACT2* (shown). Results were similar independent of control used. Samples were measured in technical triplicates on biological triplicates.

### Plant materials, growth conditions and imaging

Plants were grown in long-day conditions at 20°C. Mutant lines, *blr* (*van-1*) [2], *rev-6*[46], and *bp-1*[22,42], have been previously described. Digital images of the plants were analysed in *Scion Image* (Scion Corporation, Maryland, USA) for hypocotyl measurements. For histological analysis of *BLR-OX* and *BLR-GR*, plants were fixed, wax-embedded and sectioned using standard methods. Sections between 6 and 10 micrometers were stained with toluidine blue for light microscopy. For visualisation of hypocotyl epidermal cells, seedlings were stained with 10 μg ml^−1^ propidium iodide (Molecular Probes Europe BV, Netherlands) for 10 minutes in the dark before transfer to water. Seedlings were viewed by confocal microscopy on a Zeiss (Welwyn, UK) CLSM 510.

Toluidine blue stained resin sections for visualisation of transverse vascular bundle sections in inflorescence stems were taken from 5 week old plants. Sections were taken above the first cauline leaf and prepared as described in [59].

## Authors’ contributions

AMB and HGD designed the experiments. JPE, LM, WZJ, HP, NC, NS and AMB carried out the experiments. JPE, AMB and HGD wrote the manuscript. All authors read and approved the final manuscript.

## Authors’ information

Anuj M Bhatt and Hugh G Dickinson Joint last authors.

## Supplementary Material

Additional file 1 Genes changed in expression in BLR-OX in the same direction in all 3 replicates with an average of >1.5-fold change, arranged in order of spot confidence. Click here for file
