# Supplementary material for: A role for BELLRINGER in cell wall development is supported by loss-of-function phenotypes
Source: BMC Plant Biol. 2012 Nov 13;12:212. doi: 10.1186/1471-2229-12-212 (PMC3538058; doi:10.1186/1471-2229-12-212)
Supplement: Additional file 1 — Genes changed in expression in BLR-OX in the same direction in all 3 replicates with an average of >1.5-fold change, arranged in order of spot confidence. [file 1471-2229-12-212-S1.pdf]

Genes changed in expression in BLR-OX in the same direction in all 3 replicates with an average of >1.5-fold change, arranged in order of spot confidence

Upregulated genes

| SPOTNUMBER | BLOCK | NAME | AG ID | BLR-OX (1) | Col-0 (1) | BLR-OX (2) | Col-0 (2) | BLR-OX (3) | Col-0 (3) | Mean Ratio (BLR-OX:Col-0) | Spot Confidence |
|---|---|---|---|---|---|---|---|---|---|---|---|
| 3912 | 7 | expressed protein similar to hypothetical protein GB:CAB80917 GI:7267605 from [Arabidopsis thaliana] | At1g01430 | 1787.80183 | 1147.219 | 1552.76854 | 1034.998 | 2896.35392 | 1655.476 | 1.602733601 | 0.803712 |
| 261 | 1 | trypsin inhibitor, putative similar to SP\|P26780 Trypsin inhibitor 2 precursor (MTI-2) {Sinapis alba} | At2g43520 | 2623.64804 | 1356.269 | 2447.05036 | 1903.594 | 1752.37643 | 1232.123 | 1.547397015 | 0.778414 |
| 13777 | 22 | expressed protein weak similarity to SP\|P53809 Phosphatidylcholine transfer protein (PC-TP) {Rattus norvegicus} | At1g64720 | 5778.65146 | 4729.678 | 3573.64263 | 2901.194 | 5432.67396 | 2605.493 | 1.512884545 | 0.76076 |
| 143 | 1 | band 7 family protein strong similarity to hypersensitive-induced response protein [Zea mays] GI:7716466; contains Pfam profile PF01145: SPFH domain / Band 7 family | At1g69840 | 934.878854 | 463.826 | 433.947088 | 365.051 | 1183.55169 | 682.818 | 1.645881681 | 0.743728 |
| 5793 | 9 | expressed protein similar to unknown protein (pir\|\|S76207) | At5g51020 | 789.769087 | 559.846 | 560.420118 | 483.709 | 890.795804 | 460.651 | 1.501018393 | 0.73692 |
| 27 | 1 | thioredoxin family protein contains Pfam profile: PF00085 Thioredoxin; similar to ESTs gb\|T46281, gb\|R83933, gb\|N65879, emb\|F14466, gb\|N96726, gb\|AA042340, and emb\|Z18150 | At1g08570 | 1353.88436 | 662.757 | 1128.70825 | 799.401 | 936.390775 | 737.109 | 1.575035001 | 0.735816 |
| 11720 | 19 | UDP-glucose transferase (UGT75B2) similar to UDP-glucose:indole-3-acetate beta-D-glucosyltransferase GI:2149127 from (Arabidopsis thaliana); identical to cDNA UDP-glucosyltransferase (UGT75B2) GI:13661274 | At1g05560 | 985.290944 | 765.769 | 852.459289 | 673.083 | 2681.85157 | 861.069 | 1.889242905 | 0.72891 |
| 23208 | 36 | MERI-5 protein (MERI-5) (MERI5B) / endo-xyloglucan transferase / xyloglucan endo-1,4-beta-D-glucanase (SEN4) identical to endo-xyloglucan transferase gi:944810, SP\|P24806 MERI-5 protein precursor (Endo-xyloglucan transferase) (Xyloglucan endo-1,4-beta-D-glucanase) {Arabidopsis thaliana} | At4g30270 | 1472.93189 | 555.304 | 1890.00339 | 564.614 | 3446.82975 | 447.573 | 4.567020224 | 0.72864 |
| 29856 | 46 | expressed protein | At5g56550 | 953.785661 | 467.624 | 805.732384 | 579.965 | 2182.90695 | 1098.222 | 1.805531167 | 0.728376 |
| 13384 | 21 | alkaline alpha galactosidase, putative similar to alkaline alpha galactosidase II [Cucumis melo] GI:29838631; contains Pfam profile PF05691: Raffinose synthase or seed imbibition protein Sip1 | At3g57520 | 1275.32972 | 747.599 | 1160.01461 | 514.271 | 1283.00576 | 1123.01 | 1.701339916 | 0.7209 |
| 23377 | 36 | ripening-responsive protein, putative similar to ripening regulated protein DDTFR18 [Lycopersicon esculentum] GI:12231296; contains Pfam profile PF01554: Uncharacterized membrane protein family | At5g65380 | 892.259659 | 638.703 | 891.633662 | 729.42 | 626.72106 | 308.31 | 1.55071222 | 0.72036 |
| 251 | 1 | yippee family protein similar to mdgl-1 [Mus musculus] GI:10441648, Yippee protein [Drosophila melanogaster] GI:5713279; contains Pfam profile PF03226: Yippee putative zinc-binding protein | At2g40110 | 769.61837 | 374.016 | 603.829645 | 533.64 | 937.675344 | 601.399 | 1.582800619 | 0.7128 |
| 3038 | 5 | 26S proteasome regulatory subunit, putative (RPN9) similar to 26S proteasome subunit p40.5 [Homo sapiens] gi\|3618343\|dbj\|BAA33214 | At4g19006 | 1072.05668 | 623.653 | 803.869745 | 572.546 | 1417.85042 | 565.897 | 1.876171324 | 0.71253 |
| 11064 | 18 | methylcrotonyl-CoA carboxylase alpha chain, mitochondrial / 3-methylcrotonyl-CoA carboxylase 1 (MCCA) nearly identical to SP\|Q42523 Methylcrotonyl-CoA carboxylase alpha chain, mitochondrial precursor (EC 6.4.1.4) (3-Methylcrotonyl-CoA carboxylase 1) (MCCase alpha subunit) (3-methylcrotonyl-CoA:carbon dioxi | At1g03090 | 910.623123 | 429.567 | 700.803189 | 352 | 3579.37332 | 1203.481 | 2.361654827 | 0.712356 |
| 18415 | 29 | sinapoylglucose:malate sinapoyltransferase (SNG1) similar to serine carboxypeptidase I precursor (SP:P37890) [Oryza sativa]; contains Pfam profile PF00450: Serine carboxypeptidase; identical to cDNA sinapoylglucose:malate sinapoyltransferase (SNG1) GI:8699618 | At2g22990 | 4085.189 | 2704 | 2941.75071 | 1695.299 | 4667.97208 | 1862.184 | 1.917584609 | 0.711822 |
| 767 | 2 | expressed protein | At1g60010 | 9712.49188 | 4649.268 | 7498.23099 | 6174.497 | 9932.44589 | 5086.816 | 1.752003388 | 0.700245 |
| 2815 | 5 | sinapoylglucose:malate sinapoyltransferase (SNG1) similar to serine carboxypeptidase I precursor (SP:P37890) [Oryza sativa]; contains Pfam profile PF00450: Serine carboxypeptidase; identical to cDNA sinapoylglucose:malate sinapoyltransferase (SNG1) GI:8699618 | At2g22990 | 5398.73235 | 3458.197 | 3257.97392 | 2537.054 | 3825.25791 | 2175.012 | 1.534675553 | 0.693966 |
| 4203 | 7 | serine carboxypeptidase S10 family protein contains Pfam profile: PF00450 serine carboxypeptidase; similar to serine carboxypeptidase I precursor (SP:P37890) [Oryza sativa] | At3g12230 | 2776.2309 | 1719.291 | 1840.89122 | 1297.522 | 2964.02162 | 1506.091 | 1.667183616 | 0.68904 |
| 13379 | 21 | strictosidine synthase family protein similar to strictosidine synthase [Rauvolfia serpentina][SP\|P15324] | At3g57020 | 1033.37886 | 687.06 | 607.430641 | 503.934 | 1121.54599 | 422.16 | 1.788707045 | 0.68904 |
| 18180 | 28 | expressed protein similar to hypothetical protein GB:CAB80917 GI:7267605 from [Arabidopsis thaliana] | At1g01431 | 3364.08697 | 1830.95 | 2369.03522 | 1743.529 | 3672.4817 | 1682.677 | 1.903980553 | 0.688344 |
| 14032 | 22 | trypsin inhibitor, putative similar to SP\|P26780 Trypsin inhibitor 2 precursor (MTI-2) {Sinapis alba} | At2g43521 | 3465.72434 | 1873.31 | 2429.35368 | 1790.431 | 3767.31092 | 1721.287 | 1.908170305 | 0.68714 |
| 24315 | 38 | expressed protein weak similarity to SP\|P53809 Phosphatidylcholine transfer protein (PC-TP) {Rattus norvegicus} | At1g74960 | 3567.36171 | 1915.67 | 2489.67215 | 1837.332 | 3862.14015 | 1759.898 | 1.912360057 | 0.681384 |
| 19018 | 30 | zinc finger (B-box type) family protein similar to zinc finger protein GI:3618316 from [Oryza sativa] | At1g78600 | 1324.91289 | 1124.333 | 1727.28459 | 1456.649 | 1702.24939 | 675.64 | 1.627884801 | 0.68034 |
| 14659 | 23 | asparagine synthetase 1 [glutamine-hydrolyzing] / glutamine-dependent asparagine synthetase 1 (ASN1) identical to SP\|P49078 Asparagine synthetase [glutamine-hydrolyzing] (EC 6.3.5.4) (Glutamine- dependent asparagine synthetase) {Arabidopsis thaliana} | At3g47340 | 1266.23944 | 777.959 | 1643.81796 | 1443.662 | 3379.26308 | 799.412 | 2.331157781 | 0.67983 |
| 11918 | 19 | ABC transporter family protein similar to PDR5-like ABC transporter GI:1514643 from [Spirodela polyrhiza] | At2g26910 | 1151.06161 | 957.544 | 1331.99403 | 960.898 | 1786.05976 | 913.899 | 1.514208158 | 0.679272 |
| 2796 | 5 | UDP-glucoronosyl/UDP-glucosyl transferase family protein contains Pfam profile: PF00201 UDP-glucoronosyl and UDP-glucosyl transferase | At2g15490 | 656.863125 | 339.012 | 416.066391 | 367.222 | 767.000909 | 243.693 | 2.072665894 | 0.673552 |
| 29144 | 45 | polyubiquitin (UBQ4) identical to GI:17677 | At5g20620 | 10704.5532 | 7731.081 | 9411.38114 | 7622.13 | 10895.6971 | 5629.167 | 1.518311976 | 0.66928 |
| 27282 | 42 | xyloglucan:xyloglucosyl transferase, putative / xyloglucan endotransglycosylase, putative / endo-xyloglucan transferase, putative similar to endo-xyloglucan transferase GI:2244732 from [Gossypium hirsutum] | At5g65730 | 1070.29724 | 769.445 | 1416.30991 | 780.267 | 1646.2892 | 1065.555 | 1.583721976 | 0.665364 |
| 22655 | 35 | bZIP transcription factor family protein similar to seed storage protein opaque-2(bZIP family)GI:168428 from Zea mays; contains Pfam profile PF00170: bZIP transcription factor; identical to cDNA bZIP protein BZO2H3 GI:10954098 | At5g28770 | 763.301483 | 376.854 | 781.707687 | 273.982 | 742.346675 | 668.329 | 1.996447438 | 0.66521 |
| 28563 | 44 | copper transporter 1 (COPT1) nearly identical to SP\|Q39065 Copper transporter 1 (COPT1) {Arabidopsis thaliana} | At5g59030 | 826.422216 | 702.949 | 812.083521 | 663.591 | 763.877119 | 281.286 | 1.705027132 | 0.663872 |
| 15283 | 24 | expressed protein | At3g27770 | 395.281354 | 304.093 | 973.10221 | 696.513 | 1153.14679 | 618.696 | 1.520269932 | 0.66339 |
| 25923 | 40 | chaperone protein dnaJ-related similar to Chaperone protein dnaJ (SP:Q9WZV3) [Thermotoga maritima] | At5g43260 | 528.72638 | 449.406 | 748.030409 | 561.464 | 754.241823 | 325.448 | 1.608778507 | 0.658416 |
| 22572 | 35 | pectinesterase family protein contains Pfam profile: PF01095 pectinesterase | At4g33220 | 955.217817 | 691.288 | 721.054807 | 589.872 | 685.08749 | 340.709 | 1.538318815 | 0.657888 |
| 18068 | 28 | alpha-glucosidase 1 (AGLU1) identical to alpha-glucosidase 1 [Arabidopsis thaliana] GI:2323344 | At5g11720 | 1126.01333 | 633.312 | 1204.73069 | 690.89 | 832.977841 | 410.758 | 1.84987238 | 0.657888 |
| 8444 | 13 | importin alpha-1 subunit, putative similar to importin alpha-1 subunit (Karyopherin alpha-1 subunit, KAP alpha) [Arabidopsis thaliana] SWISS-PROT:Q96321 | At1g32880 | 1117.83218 | 660.835 | 1114.45882 | 683.742 | 727.509185 | 453.906 | 1.641420208 | 0.65772 |
| 17151 | 27 | zinc finger (CCCH-type) family protein contains Pfam domain, PF00642: Zinc finger C-x8-C-x5-C-x3-H type (and similar) and Pfam domain, PF00023: Ankyrin repeat | At2g40140 | 3845.94522 | 1852.075 | 4059.06325 | 2057.915 | 3265.53442 | 1333.214 | 2.166115211 | 0.657443 |
| 21322 | 33 | nodulin family protein similar to nodulin-like protein [Arabidopsis thaliana] GI:3329368, nodule-specific protein Nlj70 [Lotus japonicus] GI:3329366 | At5g14120 | 2639.33543 | 1674.732 | 2729.70304 | 1558.574 | 3226.09631 | 1527.239 | 1.813252343 | 0.65736 |
| 9639 | 15 | raffinose synthase family protein / seed imbibition protein, putative (din10) similar to seed imbibition protein GB:AAA32975 GI:167100 from [Hordeum vulgare]; contains nonconsensus AT donor splice site at intron 1; contains Pfam profile PF05691: Raffinose synthase or seed imbibition protein Sip1; identical to cDNA see | At5g20250 | 3425.05727 | 1433.546 | 3229.69654 | 735.179 | 7815.1468 | 5532.069 | 2.731664712 | 0.65736 |
| 13322 | 21 | glycolipid transfer protein-related contains weak similarity to Glycolipid transfer protein (GLTP) (Swiss-Prot:P17403) [Sus scrofa] | At3g21260 | 877.663223 | 512.159 | 897.580085 | 682.184 | 858.984687 | 396.788 | 1.731414697 | 0.6561 |
| 6014 | 10 | wound-responsive protein, putative similar to wound-responsive protein 14.05 (GI:16506638) [Castanea sativa] | At1g77310 | 4025.0866 | 2583.169 | 3413.43337 | 2548.594 | 3432.41495 | 1992.317 | 1.540120888 | 0.653883 |
| 7854 | 13 | 12-oxophytodienoate reductase, putative similar to OPR1 [GI:3882355] and OPR2 [GI:3882356] | At1g18020 | 2821.39995 | 1819.391 | 1602.53398 | 1018.547 | 2975.52294 | 1339.871 | 1.781614984 | 0.64989 |
| 5043 | 8 | homeodomain protein (BELLRINGER) several homeodomain proteins; | At5g02030 | 1997.31154 | 633.566 | 2116.11884 | 540.4 | 3408.33392 | 946.186 | 3.556837127 | 0.649194 |
| 5461 | 9 | trypsin inhibitor, putative similar to SP\|P26780 Trypsin inhibitor 2 precursor (MTI-2) {Sinapis alba} | At2g43510 | 616.755282 | 425.664 | 658.437313 | 505.848 | 1171.86273 | 200.919 | 2.861029648 | 0.648 |
| 26220 | 41 | CBL-interacting protein kinase 3 (CIPK3) identical to CBL-interacting protein kinase 3 [Arabidopsis thaliana] gi\|9280638\|gb\|AAF86507 | At2g26980 | 802.937645 | 449.692 | 560.38347 | 322.618 | 1524.53354 | 814.292 | 1.798245101 | 0.64792 |
| 3836 | 6 | glycosyl hydrolase family 3 protein | At5g49360 | 2157.03913 | 629.007 | 1057.0803 | 381.654 | 3101.16737 | 1940.799 | 2.598964415 | 0.645606 |
| 1931 | 3 | asparagine synthetase 2 (ASN2) identical to asparagine synthetase (ASN2) [Arabidopsis thaliana] GI:3859536 | At5g65010 | 2723.47645 | 1608.764 | 2191.69992 | 1841.95 | 7634.68655 | 2981.252 | 1.814559866 | 0.64328 |
| 17040 | 27 | UbiE/COQ5 methyltransferase family protein low similarity to SP\|Q05197 Phosphatidylethanolamine N-methyltransferase (EC 2.1.1.17) [Rhodopseudomonas sphaeroides] {Rhodobacter sphaeroides}; contains Pfam profile PF01209: methlytransferase, UbiE/COQ5 family | At1g69526 | 1608.70751 | 708.456 | 1481.00356 | 1057.31 | 917.274826 | 647.485 | 1.696041525 | 0.642936 |
| 28816 | 45 | serine carboxypeptidase S10 family protein similar to serine carboxypeptidase I precursor (SP:P37890) [Oryza sativa] | At2g23000 | 3089.55744 | 2281.431 | 2883.53886 | 2045.557 | 2347.01627 | 1084.925 | 1.642392258 | 0.642816 |
| 29215 | 45 | xyloglucan:xyloglucosyl transferase / xyloglucan endotransglycosylase / endo-xyloglucan transferase (TCH4) identical to xyloglucan endotransglycosylase TCH4 protein GI:886116 | At5g57560 | 486.388482 | 186.933 | 705.078176 | 250.232 | 1814.01923 | 1263.888 | 2.284969009 | 0.642005 |
| 9042 | 14 | pyridoxamine 5'-phosphate oxidase-related contains weak similarity to Pyridoxamine 5'-phosphate oxidase (EC 1.4.3.5) (PNP/PMP oxidase) (PNPOx). (Swiss-Prot:P28225) [Shigella flexneri] | At5g49970 | 868.588983 | 764.514 | 677.917745 | 596.381 | 848.37617 | 254.988 | 1.866657792 | 0.639821 |
| 27738 | 43 | glutathione S-transferase-related contains weak hit to Pfam profile PF00043: Glutathione S-transferase, C-terminal domain | At4g19880 | 1322.62508 | 1030.956 | 1816.56857 | 1602.899 | 1337.73086 | 601.011 | 1.547338063 | 0.638664 |
| 25005 | 39 | cytochrome P450, putative similar to GB:Q05047 from [Catharanthus roseus] | At3g14690 | 3411.44163 | 2466.579 | 3114.7228 | 2416.425 | 3480.86979 | 1371.652 | 1.736588841 | 0.63648 |
| 23654 | 37 | ACT domain-containing protein contains Pfam ACT domain PF01842 | At2g39570 | 547.189315 | 311.386 | 586.720134 | 266.541 | 557.954367 | 317.346 | 1.90556578 | 0.63597 |
| 25128 | 39 | monooxygenase, putative (MO1) similar to GI:3426062 | At4g15760 | 1479.21241 | 864.126 | 2302.10869 | 1345.038 | 1117.17474 | 743.609 | 1.641908964 | 0.63495 |
| 7793 | 12 | thioesterase family protein contains Pfam profile PF03061: thioesterase family protein | At1g35250 | 1424.72633 | 950.977 | 1076.10663 | 658.437 | 993.025202 | 699.994 | 1.517041917 | 0.63495 |
| 5550 | 9 | nitrilase 2 (NIT2) identical to SP\|P32962 Nitrilase 2 (EC 3.5.5.1) {Arabidopsis thaliana} | At3g44300 | 6358.29049 | 5358.119 | 5582.12357 | 4930.994 | 5720.70807 | 2614.229 | 1.502336489 | 0.63468 |
| 13062 | 21 | expressed protein similar to TolB protein precursor (SP:Q9ZDM5) {Rickettsia prowazekii}; ESTs gb\|N96028, gb\|F14286, gb\|T20680, gb\|F14443, gb\|AA657300 and gb\|N65244 come from this gene | At1g21680 | 2892.84943 | 1900.774 | 1354.90178 | 956.867 | 2643.44028 | 1226.645 | 1.697642008 | 0.63427 |
| 29968 | 47 | expressed protein | At1g25275 | 5657.46693 | 3620.371 | 6573.76134 | 4679.333 | 5921.55853 | 3568.766 | 1.542266502 | 0.63168 |
| 246 | 1 | expressed protein contains Pfam profile PF04749: Protein of unknown function, DUF614 | At2g37110 | 2673.23977 | 1647.646 | 1384.2961 | 1248.984 | 2677.62053 | 1054.476 | 1.756695975 | 0.628488 |
| 15886 | 25 | expressed protein identical to unknown protein GB:AAF30301 from [Arabidopsis thaliana] | At3g06080 | 652.093908 | 427.992 | 701.649263 | 402.293 | 1036.22836 | 487.223 | 1.798180812 | 0.628227 |
| 25059 | 39 | asparagine synthetase 1 [glutamine-hydrolyzing] / glutamine-dependent asparagine synthetase 1 (ASN1) identical to SP\|P49078 Asparagine synthetase [glutamine-hydrolyzing] (EC 6.3.5.4) (Glutamine- dependent asparagine synthetase) {Arabidopsis thaliana} | At3g47340 | 1628.7962 | 1342.263 | 1730.76289 | 1449.36 | 2885.29178 | 1270.729 | 1.559402279 | 0.627792 |
| 5430 | 9 | BTB/POZ domain-containing protein contains Pfam PF00651: BTB/POZ domain; contains Interpro IPR000210/ PS50097: BTBB/POZ domain; similar to MigA (GI:1841872) [Dictyostelium discoideum] | At2g30600 | 2238.72762 | 1095.847 | 1404.06015 | 506.57 | 4232.00637 | 1728.942 | 2.420787815 | 0.627264 |
| 11863 | 19 | auxin efflux carrier family protein contains auxin efflux carrier domain, Pfam:PF03547 | At1g76530 | 686.151188 | 587.824 | 729.69401 | 559.437 | 1050.10706 | 489.085 | 1.539564837 | 0.62656 |
| 13360 | 21 | expressed protein | At3g47295 | 2500.44736 | 2025.918 | 2629.8726 | 1903.631 | 3391.93145 | 1649.575 | 1.557326107 | 0.624624 |
| 22354 | 35 | expressed protein similar to GI:7572912 (At3g56140)[Arabidopsis thaliana] | At2g40400 | 712.019832 | 539.166 | 598.714956 | 463.586 | 627.558195 | 315.125 | 1.534513027 | 0.620928 |
| 2689 | 5 | expressed protein | At1g42550 | 459.282344 | 288.533 | 323.004549 | 262.13 | 423.468479 | 233.077 | 1.546958565 | 0.620928 |
| 30909 | 48 | LOB domain protein 38 / lateral organ boundaries domain protein 38 (LBD38) identical to SP\|Q9SN23 LOB domain protein 38 {Arabidopsis thaliana} | At3g49940 | 1131.1323 | 579.119 | 586.868317 | 383.556 | 1580.78445 | 1221.565 | 1.59244398 | 0.620508 |
| 19258 | 30 | expressed protein | At4g08555 | 2331.85623 | 1282.003 | 3549.66574 | 1989.898 | 4761.426 | 929.918 | 2.907674595 | 0.619875 |
| 29310 | 46 | glycosyl transferase family 20 protein / trehalose-phosphatase family protein contains Pfam profile: PF02358 trehalose-phosphatase | At1g23870 | 530.708295 | 232.296 | 609.951338 | 214.063 | 885.509072 | 694.22 | 2.136522422 | 0.6188 |
| 25908 | 40 | jacalin lectin family protein similar to myrosinase-binding protein homolog [Arabidopsis thaliana] GI:2997767; contains Pfam profile PF01419 jacalin-like lectin domain | At5g35940 | 764.796744 | 523.723 | 1371.68784 | 704.988 | 555.249143 | 268.065 | 1.82577338 | 0.61659 |
| 13435 | 21 | expressed protein | At4g18740 | 798.067889 | 616.031 | 892.212052 | 602.189 | 1128.38328 | 458.989 | 1.745174834 | 0.613524 |
| 30816 | 48 | F-box family protein contains Pfam:PF00646 F-box domain | At2g16365 | 689.173796 | 576.787 | 558.286607 | 385.442 | 875.750061 | 361.51 | 1.688586685 | 0.613008 |
| 22036 | 34 | protease inhibitor/seed storage/lipid transfer protein (LTP) family protein contains Pfam protease inhibitor/seed storage/LTP family domain PF00234 | At5g48485 | 1041.74825 | 928.492 | 1409.61142 | 932.325 | 1861.36064 | 700.336 | 1.763906988 | 0.612765 |
| 15503 | 24 | expressed protein similar to PGPS/D12 [Petunia x hybrida] GI:4105794; contains Pfam profile PF04749: Protein of unknown function, DUF614 | At5g35525 | 970.484873 | 519.39 | 891.281529 | 596.649 | 905.596179 | 496.564 | 1.72868204 | 0.61236 |
| 16016 | 25 | major intrinsic family protein / MIP family protein contains Pfam profile: MIP PF00230 | At4g10380 | 561.530123 | 332.347 | 551.787288 | 267.291 | 832.227307 | 635.403 | 1.687907313 | 0.61232 |
| 2091 | 4 | glutamate:glyoxylate aminotransferase 2 (GGT2) identical to glutamate:glyoxylate aminotransferase 2 [Arabidopsis thaliana] GI:24461829; similar to alanine aminotransferase from Panicum miliaceum [SP\|P34106], GI:4730884 from Oryza sativa; contains Pfam profile PF00155: aminotransferase, classes I and II | At1g70580 | 1605.87743 | 1219.415 | 1435.76963 | 1288.218 | 3091.5197 | 1461.668 | 1.515508881 | 0.61194 |
| 27784 | 43 | DNAJ heat shock N-terminal domain-containing protein (J11) identical to dnaJ heat shock protein J11 [Arabidopsis thaliana] GI:9843641; contains Pfam profile PF00226 DnaJ domain | At4g36040 | 6067.5704 | 2534.588 | 5921.10156 | 3749.741 | 6056.10834 | 2940.255 | 2.010899862 | 0.611064 |
| 10690 | 17 | glutathione S-transferase, putative similar to glutathione transferase GB:CAA71784 [Glycine max] | At3g09270 | 2258.40451 | 1211.734 | 898.477148 | 728.693 | 766.04006 | 380.263 | 1.703759315 | 0.60588 |
| 11218 | 18 | nitrate reductase 1 (NR1) identical to SP\|P11832 Nitrate reductase 1 (formerly EC 1.6.6.1) (NR1){Arabidopsis thaliana} | At1g77760 | 892.354851 | 558.081 | 702.104964 | 506.615 | 2472.81121 | 860.714 | 1.952607191 | 0.605734 |
| 16695 | 26 | RWP-RK domain-containing protein similar to nodule inception protein [Lotus japonicus] GI:6448579; contains Pfam profile: PF02042 RWP-RK domain | At4g24020 | 502.406377 | 315.936 | 322.330557 | 240.203 | 662.688984 | 301.09 | 1.711030352 | 0.60544 |
| 2513 | 4 | proline oxidase, putative / osmotic stress-responsive proline dehydrogenase, putative similar to proline oxidase, mitochondrial precursor (Osmotic stress- induced proline dehydrogenase) [Arabidopsis thaliana] SWISS-PROT:P92983 | At5g38710 | 744.11231 | 447.057 | 912.673994 | 303.915 | 1992.80109 | 1199.205 | 2.109764596 | 0.60544 |
| 201 | 1 | expressed protein similar to myo-inositol oxygenase [Sus scrofa] gi\|17432544\|gb\|AAL39076 | At2g19800 | 1100.58535 | 323.853 | 1620.5055 | 364.496 | 3592.97779 | 1413.754 | 3.461911706 | 0.60291 |
| 820 | 2 | phosphoribulokinase/uridine kinase-related | At1g80380 | 1104.01974 | 540.526 | 664.823504 | 559.588 | 1859.54204 | 844.766 | 1.81060047 | 0.601996 |
| 19351 | 30 | monooxygenase, putative (MO3) similar to GI:3426064; identical to cDNA monooxygenase 3, partial GI:3426065 | At5g05320 | 1097.7296 | 735.625 | 1046.35858 | 895.174 | 1208.16262 | 429.485 | 1.824726284 | 0.598347 |
| 23175 | 36 | disease resistance protein (TIR-NBS class), putative domain signature TIR-NBS exists, suggestive of a disease resistance protein. | At4g16990 | 926.48807 | 704.352 | 1186.26045 | 810.076 | 433.654305 | 243.966 | 1.519092536 | 0.596687 |
| 7233 | 12 | thioesterase family protein contains Pfam profile PF03061: thioesterase family protein | At1g35250 | 2695.23656 | 1734.294 | 2208.22786 | 1380.021 | 2557.77625 | 1644.243 | 1.569939551 | 0.591948 |
| 3690 | 6 | 26.5 kDa class P-related heat shock protein (HSP26.5-P) contains Pfam profile: PF00011 Hsp20/alpha crystallin family: identified in Scharf, K-D., et al,Cell Stress & Chaperones (2001) 6: 225-237. | At4g21870 | 411.455407 | 302.512 | 435.143702 | 267.889 | 1679.07973 | 666.477 | 1.83460295 | 0.59168 |
| 17266 | 27 | nonspecific lipid transfer protein 5 (LTP5) identical to SP\|Q9XFS7 Nonspecific lipid-transfer protein 5 (LTP 5) {Arabidopsis thaliana} | At3g51600 | 5398.66818 | 3712.602 | 5251.07732 | 2785.961 | 8282.53804 | 3761.998 | 1.84687157 | 0.5916 |
| 4067 | 7 | multi-copper oxidase type I family protein similar to pollen-specific BP10 protein [SP\|Q00624][Brassica napus]; contains Pfam profile: PF00394 Multicopper oxidase | At1g76160 | 1004.03945 | 662.054 | 694.085579 | 434.87 | 1614.59355 | 1059.801 | 1.545371902 | 0.59136 |
| 25231 | 39 | NADP-dependent oxidoreductase, putative (P1) identical to probable NADP-dependent oxidoreductase P1, zeta-crystallin homolog [SP\|Q39172][gi:886428], Arabidopsis thaliana; similar to allyl alcohol dehydrogenase [Nicotiana tabacum] GI:6692816; contains Pfam profile PF00107: oxidoreductase, zinc-binding dehydro | At5g16970 | 1258.61233 | 1026.281 | 1327.2729 | 1195.558 | 1170.273 | 444.763 | 1.655926778 | 0.590132 |
| 852 | 2 | expressed protein contains Pfam profile PF04720: Protein of unknown function (DUF506) | At2g20670 | 7021.44911 | 2262.284 | 4264.07304 | 2138.008 | 4283.63037 | 3673.593 | 2.088057782 | 0.588588 |
| 19682 | 31 | PAC motif-containing protein similar to nonphototropic hypocotyl 1 [Zea mays] GI:2687358; contains Pfam profile PF00785: PAC motif | At2g02710 | 465.756457 | 269.216 | 890.092882 | 459.015 | 585.394238 | 430.888 | 1.6759202 | 0.58548 |
| 14916 | 23 | heat shock protein-related contains similarity to 101 kDa heat shock protein; HSP101 [Triticum aestivum] gi\|11561808\|gb\|AAC83689 | At5g57710 | 494.176028 | 206.402 | 949.611289 | 606.69 | 694.172257 | 477.264 | 1.804652129 | 0.583596 |
| 27705 | 43 | methionine sulfoxide reductase domain-containing protein / SeIR domain-containing protein low similarity to pilin-like transcription factor [Homo sapiens] GI:5059062; contains Pfam profile PF01641: SelR domain | At4g04840 | 2827.81417 | 1731.896 | 3598.1294 | 2170.1 | 1472.39407 | 999.544 | 1.587966245 | 0.582505 |
| 2310 | 4 | peroxidase, putative identical to peroxidase [Arabidopsis thaliana] gi\|405611\|emb\|CAA50677 | At3g49120 | 2553.5783 | 1525.549 | 1342.45763 | 970.142 | 3740.34854 | 1999.258 | 1.642839235 | 0.580041 |
| 1084 | 2 | DREPP plasma membrane polypeptide family protein contains Pfam profile: PF05558 DREPP plasma membrane polypeptide | At4g20260 | 5287.99753 | 2748.755 | 3717.97883 | 3141.819 | 7308.2927 | 3419.365 | 1.74816261 | 0.578676 |
| 14464 | 23 | gibberellin-regulated protein 1 (GASA1) / gibberellin-responsive protein 1 identical to SP\|P46689 Gibberellin-regulated protein 1 precursor {Arabidopsis thaliana}; supporting cDNA gi\|887938\|gb\|U11766.1\|ATU11766 | At1g75750 | 5861.81702 | 3176.735 | 6384.73137 | 1882.801 | 20423.8565 | 2863.992 | 4.122523109 | 0.578676 |
| 27218 | 42 | transferase family protein similar to anthocyanin 5-aromatic acyltransferase from Gentiana triflora GI:4185599, malonyl CoA:anthocyanin 5-O-glucoside-6'''-O-malonyltransferase from Perilla frutescens GI:17980232, Salvia splendens GI:17980234; contains Pfam profile PF02458 transferase family | At5g39080 | 1258.34066 | 1078.81 | 1292.97102 | 1042.073 | 1155.63758 | 514.806 | 1.550661904 | 0.578 |
| 6207 | 10 | transferase family protein similar to hypersensitivity-related hsr201 protein - Nicotiana tabacum,PIR2:T03274; contains Pfam transferase family domain PF00248 | At3g48720 | 761.248977 | 448.328 | 575.080632 | 325.308 | 475.657433 | 387.899 | 1.564005754 | 0.57749 |
| 21674 | 34 | superoxide dismutase [Cu-Zn], chloroplast (SODCP) / copper/zinc superoxide dismutase (CSD2) identical to GP:3273753:AF061519 | At2g28190 | 313.944758 | 267.414 | 359.487735 | 264.551 | 818.942434 | 226.895 | 2.047402668 | 0.57749 |
| 63 | 1 | gibberellin-responsive protein, putative similar to SP\|P46688 Gibberellin-regulated protein 2 precursor {Arabidopsis thaliana}; contains Pfam profile PF02704: Gibberellin regulated protein | At1g22690 | 5772.83085 | 2874.644 | 5339.29694 | 3178.644 | 15275.8227 | 2733.033 | 3.092420026 | 0.577125 |
| 26376 | 41 | expressed protein | At3g54880 | 1286.65006 | 997.735 | 1616.9523 | 969.385 | 691.337133 | 425.018 | 1.52806545 | 0.57596 |
| 12653 | 20 | beta-galactosidase, putative / lactase, putative similar to beta-galactosidase precursor SP:P48980 from [Lycopersicon esculentum] | At3g13750 | 857.001251 | 486.281 | 951.026193 | 378.085 | 1687.11292 | 1428 | 1.819728797 | 0.5742 |
| 9601 | 15 | myb family transcription factor contains PFAM profile: PF00249 myb-like DNA binding domain | At5g02840 | 744.669556 | 565.468 | 529.04684 | 418.472 | 2038.59194 | 673.221 | 1.869753373 | 0.57239 |
| 1826 | 3 | expressed protein | At5g14730 | 492.986665 | 328.463 | 494.859686 | 411.892 | 607.691711 | 331.521 | 1.511787184 | 0.57154 |
| 14606 | 23 | expressed protein | At3g14760 | 1544.33431 | 1337.916 | 1612.01129 | 1289.866 | 1983.62148 | 899.683 | 1.536278294 | 0.57104 |
| 12657 | 20 | UDP-glucoronosyl/UDP-glucosyl transferase family protein contains Pfam profile: PF00201 UDP-glucoronosyl and UDP-glucosyl transferase | At3g16520 | 581.170655 | 521.563 | 1253.30271 | 742.82 | 912.17779 | 528.493 | 1.509169025 | 0.570459 |
| 21744 | 34 | no apical meristem (NAM) family protein similar to to NAC2 (GI:645671) [Arabidopsis thaliana]; contains Pfam PF02365: No apical meristem (NAM) protein | At3g10500 | 445.316955 | 291.904 | 258.444748 | 217.953 | 820.77547 | 285.681 | 1.861463497 | 0.570459 |
| 14831 | 23 | NADP-dependent oxidoreductase, putative strong similarity to probable NADP-dependent oxidoreductase (zeta-crystallin homolog) P1 [SP\|Q39172][gi:886428] and P2 [SP\|Q39173][gi:886430], Arabidopsis thaliana | At5g16990 | 1482.49065 | 1121.556 | 1702.94281 | 1283.197 | 2004.54572 | 673.611 | 1.874915444 | 0.565812 |
| 7999 | 13 | expressed protein | At2g15960 | 3528.16819 | 2192.825 | 4258.06766 | 2915.838 | 6097.55269 | 4166.397 | 1.510930464 | 0.56416 |
| 29896 | 46 | nitrate reductase 2 (NR2) identical to SP\|P11035 Nitrate reductase 2 (formerly EC 1.6.6.1) (NR2) {Arabidopsis thaliana} | At1g37130 | 405.684002 | 238.987 | 847.123123 | 461.811 | 1291.66783 | 628.869 | 1.861939641 | 0.56406 |
| 19332 | 30 | cytochrome P450, putative similar to cytochrome p450 SP:O65790 from [Arabidopsis thaliana] | At4g37410 | 4505.88764 | 2571.39 | 1162.9209 | 720.054 | 2973.00947 | 1622.195 | 1.733356846 | 0.56232 |
| 14542 | 23 | mitochondrial transcription termination factor-related / mTERF-related contains Pfam profile PF02536: mTERF | At2g36000 | 1518.72451 | 1077.916 | 1799.00176 | 1203.792 | 2296.61086 | 847.979 | 1.870575141 | 0.561946 |
| 12379 | 20 | Toll-Interleukin-Resistance (TIR) domain-containing protein domain signature TIR exists, suggestive of a disease resistance protein. | At1g09665 | 1178.21695 | 819.22 | 1466.46573 | 790.368 | 1562.95101 | 1084.63 | 1.578212894 | 0.5607 |
| 24710 | 39 | CBL-interacting protein kinase 9 (CIPK9) identical to CBL-interacting protein kinase 9 [Arabidopsis thaliana] gi\|13249117\|gb\|AAK16684; contains Pfam profiles PF00069: Protein kinase domain and PF03822: NAF domain; identical to cDNA CBL-interacting protein kinase 9 (CIPK9) GI:13249116 | At1g01140 | 366.797609 | 190.738 | 843.810525 | 634.072 | 537.240439 | 322.433 | 1.640010835 | 0.558012 |
| 3517 | 6 | expressed protein | At3g01060 | 365.107687 | 244.047 | 265.425262 | 229.759 | 712.572724 | 296.043 | 1.686092929 | 0.557345 |
| 29336 | 46 | nitrate reductase 2 (NR2) identical to SP\|P11035 Nitrate reductase 2 (formerly EC 1.6.6.1) (NR2) {Arabidopsis thaliana} | At1g37130 | 1854.17192 | 1181.382 | 2816.58743 | 1785.798 | 3742.2558 | 1800.756 | 1.741622386 | 0.55695 |
| 3151 | 5 | expressed protein | At5g24570 | 1356.1818 | 1112.696 | 1278.52561 | 927.392 | 3056.17447 | 961.797 | 1.925005653 | 0.554648 |
| 1616 | 3 | expressed protein contains similarity to phosphoserine aminotransferase GB:P19689 from [Yersinia enterocolitica] | At3g19030 | 9302.09642 | 4095.432 | 7341.59404 | 4669.33 | 10661.4232 | 8966.762 | 1.677543217 | 0.554625 |
| 9036 | 14 | expressed protein similar to unknown protein (emb\|CAB82953.1) | At5g49340 | 382.345963 | 300.529 | 580.035985 | 525.189 | 1502.93711 | 662.291 | 1.548658742 | 0.552464 |
| 22001 | 34 | meprin and TRAF homology domain-containing protein / MATH domain-containing protein low similarity to ubiquitin-specific protease 12 [Arabidopsis thaliana] GI:11993471; contains Pfam profile PF00917: MATH domain | At5g26280 | 8915.87968 | 5256.638 | 8054.98956 | 5988.278 | 6168.55655 | 3543.909 | 1.593950834 | 0.552188 |
| 13189 | 21 | hypothetical protein | At2g07190 | 391.964615 | 318.068 | 438.306524 | 270.641 | 497.798201 | 281.419 | 1.54024284 | 0.551286 |
| 24740 | 39 | thioredoxin family protein similar to thioredoxin H-type from Arabidopsis thaliana SP\|P29448, Nicotiana tabacum SP\|Q07090; contains Pfam profile: PF00085 Thioredoxin | At1g11530 | 1670.44957 | 1041.457 | 1192.56219 | 1029.736 | 2247.91684 | 737.502 | 1.936697739 | 0.55112 |
| 17891 | 28 | proline oxidase, mitochondrial / osmotic stress-responsive proline dehydrogenase (POX) (PRO1) (ERD5) nearly identical to SP\|P92983 Proline oxidase, mitochondrial precursor (EC 1.5.3.-) (Osmotic stress- induced proline dehydrogenase) [Arabidopsis thaliana]; identical to cDNA proline oxidase precursor GI:1817543 | At3g30775 | 480.0855 | 339.703 | 333.84259 | 161.152 | 686.283397 | 581.604 | 1.554945108 | 0.549753 |
| 24478 | 38 | inositol polyphosphate 5-phosphatase II (IP5PII) nearly identical to inositol polyphosphate 5-phosphatase II [Arabidopsis thaliana] GI:10444263 isoform contains an AT-acceptor splice site at intron 6 | At4g18010 | 397.649385 | 295.372 | 345.692416 | 259.001 | 994.738648 | 192.747 | 2.613944157 | 0.549626 |
| 7929 | 13 | cytochrome P450, putative similar to cytochrome P450 89A2 (CYPLXIX) (SP:Q42602) [Arabidopsis thaliana];similar to cytochrome P450 (GI:438242) [Solanum melongena] | At1g64950 | 2162.92819 | 1937.22 | 1307.91675 | 1120.768 | 2172.53349 | 766.966 | 1.705375786 | 0.548049 |
| 6292 | 10 | major latex protein-related / MLP-related low similarity to major latex protein {Papaver somniferum}[GI:294060] ; contains Pfam profile PF00407: Pathogenesis-related protein Bet v I family | At4g23680 | 1587.32465 | 1149.128 | 2177.954 | 1582.808 | 2074.41966 | 878.866 | 1.705890944 | 0.54735 |

| | | | | | | | | | | |
|---|---|---|---|---|---|---|---|---|---|---|
| 23616 | 37 | serine carboxypeptidase S10 family protein similar to serine carboxypeptidase I precursor (SP:P37890) [Oryza sativa] | At2g23010 | 2219.22613 | 1714.013 | 1558.14294 | 1089.417 | 1655.6482 | 776.794 | 1.618798308 | 0.54675 |
| 17720 | 28 | kelch repeat-containing F-box family protein similar to SP\|Q9ER30 Kelch-related protein 1 (Sarcosin) {Rattus norvegicus}; contains Pfam profiles PF01344: Kelch motif, PF00646: F-box domain | At1g80440 | 1633.70874 | 799.598 | 1404.23224 | 695.271 | 1358.92933 | 930.799 | 1.840937714 | 0.543576 |
| 20757 | 32 | beta-galactosidase, putative / lactase, putative similar to beta-galactosidase precursor GI:3869280 from [Carica papaya] | At5g56870 | 1105.20055 | 282.977 | 625.728798 | 158.689 | 797.939839 | 353.94 | 3.367727681 | 0.54264 |
| 9464 | 15 | broad-spectrum mildew resistance RPW8 family protein contains Pfam PF05659: Arabidopsis broad-spectrum mildew resistance protein RPW8 | At3g50480 | 360.167339 | 287.909 | 339.943569 | 213.493 | 1154.47672 | 619.095 | 1.569350423 | 0.539616 |
| 7603 | 12 | no apical meristem (NAM) family protein (RD26) contains Pfam PF02365: No apical meristem (NAM) domain; Arabidopsis thaliana nap gene,PID:e1234813; identical to cDNA RD26 mRNA for NAM-like protein GI:15375403 | At4g27410 | 481.018166 | 226.394 | 403.5362 | 241.324 | 1187.5456 | 787.157 | 1.768507455 | 0.537516 |
| 19112 | 30 | autophagy 8e (APG8e) identical to autophagy 8e [Arabidopsis thaliana] GI:19912159; contains Pfam profile PF02991: Microtubule associated protein 1A/1B, light chain 3 | At2g45170 | 3948.08497 | 1914.459 | 3520.42119 | 1728.483 | 3589.19418 | 2378.754 | 1.869270657 | 0.536844 |
| 7090 | 11 | acetyl-CoA C-acyltransferase, putative / 3-ketoacyl-CoA thiolase, putative strong similarity to Acetoacetyl-coenzyme A thiolase (E.C. 2.3.1.9) [Raphanus sativus] GI:1542941; contains InterPro accession IPR002155: Thiolase | At5g48230 | 9105.54671 | 6615.708 | 9926.00169 | 6688.256 | 9330.33164 | 5592.62 | 1.509592001 | 0.536256 |
| 5078 | 8 | DNAJ heat shock N-terminal domain-containing protein similar to SP\|P30725 Chaperone protein dnaJ Clostridium acetobutylicum; contains Pfam profile PF00226: DnaJ domain | At5g16650 | 920.516655 | 549.73 | 841.125712 | 496.199 | 2114.64954 | 1656.558 | 1.548719559 | 0.535952 |
| 9826 | 16 | xyloglucan:xyloglucosyl transferase, putative / xyloglucan endotransglycosylase, putative / endo-xyloglucan transferase, putative (XTR4) identical to N-terminal partial sequence of xyloglucan endotransglycosylase-related protein XTR4 GI:1244754 from [Arabidopsis thaliana]; similar to endoxyloglucan transferase [Arabid | At1g32170 | 690.767456 | 326.462 | 464.565113 | 339.227 | 1196.5149 | 741.13 | 1.699949402 | 0.530868 |
| 16210 | 25 | CER1 protein, putative (WAX2) similar to maize glossy1 homolog GI:2213643 from [Oryza sativa]; contains Pfam profile PF01598: Sterol desaturase | At5g57800 | 589.737919 | 436.21 | 476.88498 | 312.251 | 395.676021 | 206.007 | 1.599966567 | 0.525888 |
| 16458 | 26 | auxin-responsive protein, putative similar to small auxin-up regulated protein SAUR (GI:3043536) [Raphanus sativus] | At2g21210 | 3059.90893 | 2400.28 | 2294.50995 | 1361.066 | 5561.07103 | 2381.446 | 1.765265773 | 0.524477 |
| 19045 | 30 | hypothetical protein | At2g18200 | 485.523199 | 332.983 | 503.782508 | 449.31 | 399.989533 | 206.195 | 1.506399487 | 0.522144 |
| 10575 | 17 | CBS domain-containing protein low similarity to SP\|Q9MYP4 5'-AMP-activated protein kinase, gamma-3 subunit (AMPK gamma-3 chain) (AMPK gamma3) {Sus scrofa}; contains Pfam profile PF00571: CBS domain | At1g80090 | 608.623334 | 410.415 | 485.739288 | 310.224 | 716.525401 | 428.664 | 1.573415737 | 0.52164 |
| 1856 | 3 | NAD-dependent epimerase/dehydratase family protein similar to sugar epimerase BlmG from Streptomyces verticillus GI:9937230; contains Pfam profile PF01370 NAD dependent epimerase/dehydratase family | At5g28840 | 1705.07158 | 1129.764 | 1832.47509 | 1384.063 | 3840.62325 | 1753.779 | 1.674374477 | 0.5214 |
| 30422 | 47 | auxin-responsive GH3 family protein similar to auxin-responsive GH3 product [Glycine max] GI:18591; contains Pfam profile PF03321: GH3 auxin-responsive promoter | At5g13370 | 703.037575 | 459.113 | 309.642623 | 223.267 | 870.360234 | 224.832 | 2.263108296 | 0.520828 |
| 26958 | 42 | jacalin lectin family protein similar to SP\|Q9SAV1 Myrosinase binding protein-like At1g52030 {Arabidopsis thaliana}; contains Pfam profile: PF01419 jacalin-like lectin domain | At3g16450 | 5265.29268 | 3223.773 | 3956.07158 | 2886.038 | 4635.42692 | 2485.196 | 1.62308275 | 0.518868 |
| 19859 | 31 | asparagine synthetase 1 [glutamine-hydrolyzing] / glutamine-dependent asparagine synthetase 1 (ASN1) identical to SP\|P49078 Asparagine synthetase [glutamine-hydrolyzing] (EC 6.3.5.4) (Glutamine- dependent asparagine synthetase) {Arabidopsis thaliana} | At3g47340 | 467.39397 | 229.677 | 1138.86274 | 363.042 | 2087.82618 | 251.607 | 4.48999031 | 0.516516 |
| 28616 | 45 | no apical meristem (NAM) family protein contains Pfam PF02365: No apical meristem (NAM) domain; similar to NAC domain protein NAM GB:AAD17313 GI:4325282 from [Arabidopsis thaliana] | At1g01720 | 1770.37531 | 1473.838 | 2360.17554 | 1886.714 | 1994.4135 | 736.52 | 1.720011192 | 0.515592 |
| 26965 | 42 | expressed protein | At3g19660 | 625.628443 | 403.134 | 630.426027 | 542.109 | 447.485584 | 213.821 | 1.602543437 | 0.514796 |
| 8630 | 14 | glycine-rich protein (GRP3S) identical to cDNA glycine-rich protein 3 short isoform (GRP3S) GI:4206766 | At2g05380 | 16050.1899 | 11608.78 | 19018.8094 | 14729.65 | 22993.2763 | 11367.69 | 1.565489883 | 0.511758 |
| 8647 | 14 | gibberellin-responsive protein, putative similar to SP\|P46689 Gibberellin-regulated protein 1 precursor {Arabidopsis thaliana}; contains Pfam profile PF02704: Gibberellin regulated protein | At2g18420 | 1057.91695 | 633.314 | 793.422077 | 330.877 | 7259.93477 | 753.934 | 4.5659288 | 0.50949 |
| 18034 | 28 | PQ-loop repeat family protein / transmembrane family protein similar to SP\|Q10482 Seven transmembrane protein 1 {Schizosaccharomyces pombe}; contains Pfam profile PF04193: PQ loop repeat | At4g36850 | 501.480129 | 235.461 | 352.387719 | 216.48 | 657.870305 | 358.637 | 1.863983296 | 0.508956 |
| 30661 | 48 | cytochrome P450, putative similar to SP:Q42569 from [Arabidopsis thaliana] | At1g55940 | 407.874483 | 294.335 | 411.931619 | 214.958 | 589.757236 | 198.995 | 2.088587738 | 0.5031 |
| 28560 | 44 | expressed protein | At5g58650 | 357.609994 | 308.912 | 440.540416 | 281.895 | 558.471906 | 281.798 | 1.567413985 | 0.502824 |
| 16701 | 26 | expressed protein | At4g27040 | 526.340167 | 458.305 | 412.291719 | 358.861 | 1756.02601 | 548.857 | 1.832254275 | 0.500976 |
| 29704 | 46 | expressed protein | At4g28080 | 2882.50989 | 2039.285 | 1681.74155 | 1171.269 | 2740.74791 | 1469.623 | 1.57141724 | 0.50049 |
| 26951 | 42 | 2-oxoisovalerate dehydrogenase / 3-methyl-2-oxobutanoate dehydrogenase / branched-chain alpha-keto acid dehydrogenase E1 beta subunit (DIN4) identical to branched chain alpha-keto acid dehydrogenase E1 beta subunit [Arabidopsis thaliana] GI:7021286 | At3g13450 | 852.767889 | 542.894 | 1126.64007 | 851.199 | 942.310513 | 487.554 | 1.609034662 | 0.49662 |
| 13215 | 21 | sinapoylglucose:malate sinapoyltransferase (SNG1) similar to serine carboxypeptidase I precursor (SP:P37890) [Oryza sativa]; contains Pfam profile PF00450: Serine carboxypeptidase; identical to cDNA sinapoylglucose:malate sinapoyltransferase (SNG1) GI:8699618 | At2g22990 | 4727.50061 | 3288.406 | 3052.85896 | 2231.705 | 5543.61904 | 2696.614 | 1.620448761 | 0.494586 |
| 19842 | 31 | cytochrome P450 family protein contains Pfam profile: PF00067 cytochrome P450 | At3g28740 | 1948.39578 | 1355.407 | 429.514358 | 290.052 | 532.469184 | 176.831 | 1.976497567 | 0.492328 |
| 23827 | 37 | integral membrane family protein contains TIGRFAM TIGR01569 : plant integral membrane protein TIGR01569; contains Pfam PF04535 : Domain of unknown function (DUF588) | At4g15610 | 1586.89575 | 916.54 | 2537.11031 | 1541.72 | 4948.5091 | 753.343 | 3.315255864 | 0.489288 |
| 16063 | 25 | expressed protein | At4g29905 | 4001.74107 | 1703.996 | 3897.2192 | 1590.37 | 4709.83912 | 3853.931 | 2.007014386 | 0.489216 |
| 23835 | 37 | expressed protein | At4g18740 | 311.151039 | 235.378 | 297.515553 | 198.571 | 441.761727 | 156.103 | 1.883380368 | 0.489168 |
| 13750 | 22 | expressed protein similar to PGPS/D12 [Petunia x hybrida] GI:4105794; contains Pfam profile PF04749: Protein of unknown function, DUF614 | At1g52200 | 640.665542 | 290.882 | 256.532714 | 157.974 | 522.615325 | 426.776 | 1.68365026 | 0.485112 |
| 29083 | 45 | catalase 2 identical to catalase 2 SP:P25819, GI:17865693 from [Arabidopsis thaliana] | At4g35090 | 3341.16332 | 2392.986 | 2266.62135 | 1832.177 | 3628.38487 | 1650.253 | 1.610678359 | 0.483807 |
| 8916 | 14 | UDP-glucoronosyl/UDP-glucosyl transferase family protein contains Pfam profile: PF00201 UDP-glucoronosyl and UDP-glucosyl transferase | At4g34135 | 1117.30488 | 803.638 | 977.613015 | 816.577 | 3149.57684 | 1020.532 | 1.891242683 | 0.482856 |
| 1525 | 3 | WRKY family transcription factor | At2g30250 | 447.30101 | 289.577 | 546.162723 | 274.967 | 576.682677 | 316.847 | 1.783673898 | 0.47712 |
| 13977 | 22 | hydrolase, alpha/beta fold family protein low similarity to 3-oxoadipate enol-lactone hydrolase [Pseudomonas sp. B13] GI:17736948, B-ketoadipate enol-lactone hydrolase [Bradyrhizobium japonicum] GI:2239060; contains Pfam profile:PF00561 abhydrolase:alpha/beta hydrolase fold | At3g24420 | 302.286434 | 267.16 | 392.623271 | 335.09 | 607.63604 | 187.542 | 1.847725218 | 0.472685 |
| 12855 | 20 | glutamate dehydrogenase 2 (GDH2) identical to glutamate dehydrogenase 2 (GDH 2) [Arabidopsis thaliana] SWISS-PROT:Q38946 | At5g07440 | 3403.08079 | 1891.486 | 4092.93332 | 2626.374 | 5715.37392 | 2574.548 | 1.859168873 | 0.471077 |
| 17967 | 28 | major latex protein-related / MLP-related low similarity to major latex protein {Papaver somniferum}[GI:294060] ; contains Pfam profile PF00407: Pathogenesis-related protein Bet v I family | At4g14060 | 1882.7776 | 827.188 | 326.956084 | 242.282 | 1673.64866 | 1007.612 | 1.762202956 | 0.47073 |
| 20031 | 31 | NADP-dependent oxidoreductase, putative strong similarity to probable NADP-dependent oxidoreductase (zeta-crystallin homolog) P1 [SP\|Q39172][gi:886428] and P2 [SP\|Q39173][gi:886430], Arabidopsis thaliana | At5g16980 | 1698.63063 | 1260.975 | 1798.39469 | 1346.834 | 2344.18527 | 754.096 | 1.930318696 | 0.46215 |
| 9509 | 15 | arginase, putative similar to Swiss-Prot:P46637 arginase (EC 3.5.3.1) [Arabidopsis thaliana] | At4g08870 | 351.727354 | 217.176 | 480.012748 | 193.88 | 629.201536 | 169.979 | 2.599005516 | 0.46136 |
| 24028 | 37 | no apical meristem (NAM) family protein contains Pfam PF02365: No apical meristem (NAM) domain; contains similarity to NAC-domain protein | At5g63790 | 2459.29669 | 1768.805 | 2794.274 | 2441.158 | 3595.37166 | 1332.859 | 1.744170418 | 0.459648 |
| 1107 | 2 | villin, putative similar to villin 2 (VLN2) [Arabidopsis thaliana] GI:3415115, villin 3 (VLN3) [Arabidopsis thaliana] GI:3415117; contains Pfam profiles PF00626: Gelsolin repeat, PF02209: Villin headpiece domain | At4g30160 | 2998.03746 | 1838.975 | 2274.20734 | 2042.05 | 2388.26886 | 1256.566 | 1.548198727 | 0.45864 |
| 4701 | 8 | integral membrane protein, putative strong similarity to integral membrane protein GI:1209756 from [Beta vulgaris]; contains Pfam profile PF00083: major facilitator superfamily protein | At1g75220 | 371.320825 | 280.851 | 237.052283 | 157.326 | 970.818453 | 494.454 | 1.597433685 | 0.458575 |
| 6345 | 10 | expressed protein | At5g01280 | 275.14607 | 222.294 | 340.233561 | 267.169 | 281.825709 | 102.195 | 1.75631983 | 0.457884 |
| 6431 | 10 | 26S proteasome regulatory subunit, putative (RPN9) contains similarity to 26S proteasome subunit p40.5 GI:3618343 from [Homo sapiens] | At5g45620 | 3773.09139 | 2160.17 | 3908.15443 | 2364.345 | 5128.658 | 1324.07 | 2.424340819 | 0.454674 |
| 23977 | 37 | serine protease inhibitor, potato inhibitor I-type family protein similar to SP\|P24076 Glu S.griseus protease inhibitor (BGIA) {Momordica charantia}; contains Pfam profile PF00280: Potato inhibitor I family | At5g43570 | 337.278519 | 178.679 | 515.343935 | 245.868 | 1205.27389 | 118.804 | 4.709567618 | 0.452709 |
| 15199 | 24 | calcium-transporting ATPase 4, plasma membrane-type / Ca2+-ATPase, isoform 4 (ACA4) identical to SP\|O22218 Calcium-transporting ATPase 4, plasma membrane-type (EC 3.6.3.8) (Ca(2+)-ATPase isoform 4) {Arabidopsis thaliana} | At2g41560 | 192.19977 | 160.194 | 634.176798 | 385.475 | 840.213077 | 450.361 | 1.570206671 | 0.451704 |
| 427 | 1 | expressed protein contains Pfam PF05421: Protein of unknown function (DUF751) | At4g16410 | 3138.19404 | 2127.783 | 2157.76133 | 1849.897 | 4777.5563 | 2437.021 | 1.533898802 | 0.451269 |
| 16397 | 26 | expressed protein contains Pfam profile: PF00561 alpha/beta hydrolase fold | At1g73750 | 266.902886 | 221.55 | 453.82586 | 306.23 | 344.47266 | 149.997 | 1.661071567 | 0.44982 |
| 23615 | 37 | sinapoylglucose:malate sinapoyltransferase (SNG1) similar to serine carboxypeptidase I precursor (SP:P37890) [Oryza sativa]; contains Pfam profile PF00450: Serine carboxypeptidase; identical to cDNA sinapoylglucose:malate sinapoyltransferase (SNG1) GI:8699618 | At2g22990 | 4646.76191 | 3162.665 | 2679.82447 | 1874.187 | 2844.85158 | 1174.999 | 1.773422406 | 0.449064 |
| 1223 | 2 | metalloendopeptidase identical to chloroplast processing enzyme metalloendopeptidase [Arabidopsis thaliana] gi\|2827039\|gb\|AAC39482 | At5g42390 | 566.642672 | 381.22 | 474.633561 | 330.218 | 777.102197 | 466.981 | 1.529274978 | 0.44772 |
| 10371 | 16 | MA3 domain-containing protein low similarity to programmed cell death 4 protein [Gallus gallus] GI:12958564; contains Pfam profile PF02847: MA3 domain | At5g63190 | 282.490814 | 222.888 | 425.366839 | 308.637 | 830.606647 | 439.573 | 1.511732801 | 0.443111 |
| 17007 | 27 | AP2 domain-containing protein RAP2.12 (RAP2.12) identical to AP2 domain containing protein GI:2281649 from [Arabidopsis thaliana] | At1g53910 | 9939.20029 | 5701.513 | 9052.98485 | 5221.677 | 8509.22008 | 4206.881 | 1.833226287 | 0.43282 |
| 16082 | 25 | senescence-associated protein (SEN1) identical to senescence-associated protein GI:1046270 from [Arabidopsis thaliana] | At4g35770 | 1619.12941 | 532.436 | 2529.72031 | 1262.767 | 3030.33876 | 1823.453 | 2.235389202 | 0.42768 |
| 2147 | 4 | zinc finger (C3HC4-type RING finger) family protein contains Pfam PF00097: Zinc finger, C3HC4 type (RING finger) domain; similar to RING-H2 finger protein RHA3a (GI:3790573) [Arabidopsis thaliana]; similar to ReMembR-H2 protein JR700 (GI:6942147) [Arabidopsis thaliana] | At2g17730 | 308.399033 | 220.022 | 360.196781 | 253.047 | 889.758664 | 455.032 | 1.593495967 | 0.42435 |
| 24134 | 38 | expressed protein | At1g36640 | 525.304823 | 314.184 | 290.499984 | 237.34 | 304.733507 | 148.833 | 1.647811369 | 0.420036 |
| 10014 | 16 | cytochrome P450 family protein | At2g45560 | 830.47482 | 674.413 | 866.672069 | 687.117 | 1274.76019 | 605.184 | 1.533040509 | 0.41888 |
| 13860 | 22 | protein phosphatase 2C, putative / PP2C, putative | At2g25070 | 926.889159 | 796.283 | 926.928202 | 765.436 | 4095.28538 | 1062.742 | 2.076169664 | 0.418656 |
| 4730 | 8 | glycine-rich protein | At2g05540 | 1613.71952 | 1076.313 | 1011.26639 | 816.128 | 3507.71377 | 1439.974 | 1.7247874 | 0.41624 |
| 2368 | 4 | xyloglucan:xyloglucosyl transferase, putative / xyloglucan endotransglycosylase, putative / endo-xyloglucan transferase, putative (XTR7) almost identical to xyloglucan endotransglycosylase-related protein XTR7 GI:1244760 from [Arabidopsis thaliana], one amino acid difference | At4g14130 | 281.026572 | 187.022 | 233.985063 | 149.863 | 722.432768 | 481.665 | 1.521277049 | 0.413859 |
| 14592 | 23 | kelch repeat-containing F-box family protein contains F-box domain Pfam:PF00646 and Kelch motif Pfam:PF01344 | At3g08810 | 461.047136 | 413.146 | 303.399835 | 245.941 | 916.167582 | 405.024 | 1.537193019 | 0.411514 |
| 11761 | 19 | proton-dependent oligopeptide transport (POT) family protein contains Pfam profile: PF00854 POT family | At1g22570 | 460.559412 | 278.783 | 343.691154 | 156.559 | 758.808949 | 429.133 | 1.871851576 | 0.411348 |
| 22790 | 36 | expressed protein | At1g13245 | 2381.87472 | 1554.505 | 3464.5213 | 1934.383 | 1832.80528 | 863.746 | 1.815062525 | 0.4004 |
| 30114 | 47 | serine carboxypeptidase S10 family protein similar to serine carboxypeptidase I precursor (SP:P37890) [Oryza sativa] | At2g22920 | 3879.00245 | 2734.365 | 3100.55463 | 2327.008 | 3346.39008 | 1877.564 | 1.511112355 | 0.396198 |
| 19164 | 30 | expressed protein | At3g20340 | 420.77351 | 192.322 | 372.258524 | 200.668 | 674.580007 | 158.394 | 2.767276519 | 0.394548 |
| 13306 | 21 | cytochrome P450, putative similar to GB:Q05047 from [Catharanthus roseus] | At3g14650 | 3853.598 | 2869.309 | 3350.94016 | 2435.775 | 4273.35175 | 2061.368 | 1.597274873 | 0.386384 |
| 2664 | 5 | AP2 domain-containing transcription factor family protein similar to TINY GB:CAA64359 GI:1246403 from [Arabidopsis thaliana] | At1g21910 | 282.811686 | 147.737 | 339.299852 | 159.379 | 530.056751 | 444.412 | 1.745297696 | 0.368456 |
| 14355 | 23 | myb family transcription factor contains Pfam profile: PF00249 myb-like DNA-binding domain | At1g18330 | 454.650032 | 375.744 | 309.62669 | 209.463 | 1202.42433 | 225.533 | 2.673223554 | 0.366244 |
| 25069 | 39 | ABC transporter family protein PDR5-like ABC transporter, Spirodela polyrrhiza, EMBL:Z70524 | At3g53480 | 636.548763 | 355.69 | 585.404018 | 460.738 | 578.895104 | 357.851 | 1.559298132 | 0.365625 |
| 6650 | 11 | serine carboxypeptidase S10 family protein similar to serine carboxypeptidase I precursor (SP:P07519) [Hordeum vulgare]; glucose acyltransferase GB:AAD01263 [Solanum berthaultii]; contains Pfam profile: PF00450 Serine carboxypeptidase | At1g73310 | 2326.95118 | 1283.12 | 2132.77265 | 1358.711 | 5151.48744 | 2317.878 | 1.868571556 | 0.35945 |
| 19303 | 30 | nodulin MtN21 family protein similar to MtN21 GI:2598575 (root nodule development) from [Medicago truncatula] | At4g28040 | 593.187285 | 496.581 | 247.275287 | 192.144 | 358.141048 | 154.41 | 1.600295311 | 0.346566 |
| 5954 | 10 | leucine-rich repeat protein kinase, putative smilar to light repressible receptor protein kinase [Arabidopsis thaliana] gi\|1321686\|emb\|CAA66376; contains leucine rich repeat (LRR) domains, Pfam:PF00560; contains protein kinase domain, Pfam:PF00069 | At1g51790 | 379.877393 | 214.591 | 159.72488 | 116.154 | 311.624404 | 168.475 | 1.665009876 | 0.337041 |
| 20824 | 33 | Ras-related protein (ARA-1) (ARA) / small GTP-binding protein, putative nearly identical to SP:P19892 Ras-related protein ARA-1 [Arabidopsis thaliana] (Gene 76:313-319(1989)) | At1g05810 | 479.503653 | 341.611 | 364.677312 | 262.713 | 512.231557 | 284.621 | 1.530490517 | 0.33264 |
| 27380 | 43 | FAD-binding domain-containing protein similar to SP\|P30986 reticuline oxidase precursor (Berberine-bridge-forming enzyme) (BBE) (Tetrahydroprotoberberine synthase) [Eschscholzia californica]; contains PF01565 FAD binding domain | At1g30700 | 320.699099 | 161.485 | 245.450889 | 222.655 | 244.880442 | 162.622 | 1.531381845 | 0.330398 |
| 23912 | 37 | lysine decarboxylase family protein contains Pfam profile PF03641: lysine decarboxylase family | At5g06300 | 268.537191 | 174.359 | 298.095536 | 195.356 | 195.868889 | 116.307 | 1.583372256 | 0.32718 |
| 27732 | 43 | expressed protein | At4g16840 | 231.253015 | 194.71 | 254.94254 | 214.939 | 281.555599 | 126.651 | 1.532292432 | 0.32376 |
| 6855 | 11 | ABC transporter family protein AbcA, Dictyostelium discoideum, DDU66526 | At3g47730 | 154.589375 | 107.213 | 162.683751 | 125.007 | 353.530622 | 175.89 | 1.584413426 | 0.3192 |
| 1605 | 3 | expressed protein | At3g15440 | 435.322884 | 271.467 | 429.566939 | 376.145 | 285.605187 | 156.448 | 1.523726272 | 0.313235 |
| 12847 | 20 | basic helix-loop-helix (bHLH) family protein contains Pfam profile: PF00010 helix-loop-helix DNA-binding domain; PMID: 12679534 | At5g04150 | 1127.06044 | 383.614 | 442.31064 | 379.482 | 214.980714 | 182.967 | 1.759513546 | 0.311904 |
| 20736 | 32 | bZIP family transcription factor similar to bZIP transcription factor GI:1769891 from [Arabidopsis thaliana] | At5g49450 | 519.021093 | 164.954 | 472.903171 | 154.894 | 373.155862 | 305.427 | 2.47376236 | 0.3042 |
| 17727 | 28 | MATE efflux family protein contains Pfam profile: PF01554 uncharacterized membrane protein family | At2g04040 | 362.53216 | 310.934 | 464.550773 | 208.78 | 267.678958 | 149.194 | 1.7283953 | 0.303408 |
| 7680 | 12 | expressed protein | At5g18490 | 199.263216 | 148.491 | 162.361893 | 98.205 | 484.131875 | 265.623 | 1.605948197 | 0.292077 |
| 5365 | 9 | expressed protein | At1g76660 | 528.718893 | 287.58 | 243.428913 | 134.603 | 563.519456 | 206.492 | 2.12533987 | 0.2805 |
| 9888 | 16 | myb family transcription factor similar to MybHv5 GI:19055 from [Hordeum vulgare] | At1g71030 | 1632.94934 | 1304.886 | 2115.07519 | 878.746 | 2151.91235 | 1258.082 | 1.789602186 | 0.27805 |
| 4722 | 8 | major latex protein-related / MLP-related low similarity to major latex protein {Papaver somniferum}[GI:169000] contains Pfam profile PF00407: Pathogenesis-related protein Bet v I family | At2g01520 | 1710.02584 | 806.227 | 168.781544 | 75.301 | 2199.02488 | 1518.703 | 1.936803404 | 0.274512 |
| 8885 | 14 | protein kinase family protein contains Pfam domain PF00069: Protein kinase domain | At4g21400 | 241.240669 | 198.283 | 239.55386 | 197.95 | 555.065634 | 240.868 | 1.577086978 | 0.27376 |
| 19538 | 31 | S-locus lectin protein kinase family protein contains Serine/Threonine protein kinases active-site signature, PROSITE:PS00108 | At1g11350 | 278.248896 | 218.101 | 319.61069 | 188.756 | 250.989875 | 146.863 | 1.559344905 | 0.273296 |
| 4895 | 8 | expressed protein | At3g45730 | 195.581753 | 98.185 | 305.879105 | 92.474 | 453.034178 | 269.74 | 2.326408123 | 0.25704 |
| 27966 | 44 | transferase family protein similar to anthranilate N-hydroxycinnamoyl/benzoyltransferase from Dianthus caryophyllus [gi:2239091]; contains Pfam transferase family domain PF002458 | At1g03390 | 504.571189 | 456.776 | 450.632765 | 368.443 | 369.452675 | 134.606 | 1.690802032 | 0.25675 |
| 14578 | 23 | TCP family transcription factor, putative similar to transcription factor PCF6 [Oryza sativa (japonica cultivar-group)] GI:20975255; contains Pfam profile PF03634: TCP family transcription factor | At3g02150 | 222.931753 | 153.754 | 180.183635 | 122.827 | 371.438293 | 156.826 | 1.761789912 | 0.253232 |
| 6759 | 11 | electron transfer flavoprotein-ubiquinone oxidoreductase family protein contains Pfam profile: PF05187 Electron transfer flavoprotein-ubiquinone oxidoreductase | At2g43400 | 154.70061 | 114.185 | 125.53454 | 85.073 | 500.2828 | 221.002 | 1.698045507 | 0.24424 |
| 16388 | 26 | expressed protein | At1g70100 | 266.592711 | 146.222 | 304.529528 | 217.766 | 366.122694 | 218.501 | 1.632413924 | 0.2431 |
| 1985 | 4 | UDP-glucose 4-epimerase / UDP-galactose 4-epimerase / Galactowaldenase identical to SP\|Q42605 [GB:CAA90941] from [Arabidopsis thaliana] (Arch. Biochem. Biophys. 327 (1), 27-34 (1996)) | At1g12780 | 193.341002 | 89.166 | 200.448001 | 115.056 | 325.503106 | 205.264 | 1.832094033 | 0.23408 |
| 21290 | 33 | zinc finger (B-box type) family protein salt-tolerance protein - Arabidopsis thaliana, PID:e224078 | At4g39070 | 318.620923 | 200.383 | 354.242391 | 177.46 | 1723.97159 | 567.156 | 2.208639741 | 0.230912 |
| 707 | 2 | expressed protein | At1g22890 | 396.402265 | 127.147 | 185.728532 | 82.632 | 760.070837 | 281.064 | 2.689863405 | 0.221312 |
| 3019 | 5 | protease inhibitor/seed storage/lipid transfer protein (LTP) family protein contains protease inhibitor/seed storage/LTP family domain, Pfam:PF00234 | At4g12545 | 1342.94693 | 659.652 | 81.3745396 | 57.294 | 778.172327 | 573.613 | 1.604251615 | 0.218592 |
| 15023 | 24 | expressed protein | At1g31835 | 222.811961 | 196.172 | 298.731287 | 201.572 | 265.427355 | 132.29 | 1.541404045 | 0.21105 |
| 19331 | 30 | cytochrome P450 family protein Similar to Cytochrome P450 91A1 (SP:Q9FG65) [Arabidopsis thaliana]; | At4g37340 | 298.921564 | 214.81 | 345.094905 | 257.478 | 174.660103 | 85.095 | 1.594794081 | 0.185334 |
| 7092 | 11 | expressed protein low similarity to extracellular dermal glycoprotein EDGP precursor [Daucus carota] GI:285741, SP\|P13917 Basic 7S globulin precursor {Glycine max}; expression supported by MPSS | At5g48430 | 125.391156 | 79.284 | 153.807137 | 128.296 | 174.09514 | 94.504 | 1.540862947 | 0.177444 |
| 30678 | 48 | RNA polymerase sigma subunit SigA (sigA) / sigma factor 1 (SIG1) identical to sigma factor SigA [Arabidopsis thaliana] GI:5478439, sigma factor 1 [Arabidopsis thaliana] GI:2353171, plastid RNA polymerase sigma-subunit [Arabidopsis thaliana] GI:2398851; contains Pfam profiles PF04545: Sigma-70, region 4, PF0453 | At1g64860 | 444.002453 | 398.77 | 528.632566 | 471.259 | 2450.04362 | 657.832 | 1.986532231 | 0.172773 |
| 19954 | 31 | xyloglucan:xyloglucosyl transferase, putative / xyloglucan endotransglycosylase, putative / endo-xyloglucan transferase, putative (XTR6) identical to xyloglucan endotransglycosylase-related protein GI:1244758 from [Arabidopsis thaliana] | At4g25810 | 187.630562 | 130.358 | 279.254042 | 135.973 | 222.797407 | 133.686 | 1.719889006 | 0.171564 |
| 14434 | 23 | expressed protein | At1g65490 | 180.55535 | 119.296 | 222.597312 | 110.992 | 238.422546 | 189.514 | 1.592368804 | 0.167586 |
| 123 | 1 | beta-fructosidase (BFRUCT3) / beta-fructofuranosidase / invertase, vacuolar identical to beta-fructosidase GB:CAA67560 GI:1429209 [Arabidopsis thaliana]; supported by full-length cDNA GI:14517549; identical to cDNA Beta-fructosidase GI:3115854 | At1g62660 | 414.706903 | 192.382 | 87.8419919 | 79.833 | 207.394954 | 137.509 | 1.588064332 | 0.14742 |
| 10377 | 16 | glycosyl hydrolase family 38 protein similar to lysosomal alpha-mannosidase SP:O09159 from [Mus musculus] | At5g66150 | 123.856322 | 102.031 | 96.0366476 | 79.121 | 363.110246 | 161.006 | 1.560987498 | 0.14616 |
| 10686 | 17 | expressed protein identical to unknown protein GB:AAF30301 from [Arabidopsis thaliana] | At3g06080 | 296.527864 | 206.547 | 170.926208 | 71.515 | 152.931716 | 103.876 | 1.765990367 | 0.145299 |
| 24447 | 38 | F-box family protein contains Pfam PF00646: F-box domain; similar to F-box protein FBL2 (GI:6063090) [Homo sapiens] | At4g02740 | 90.913535 | 73.114 | 158.085312 | 126.713 | 558.177053 | 108.442 | 2.546091711 | 0.1431 |
| 12700 | 20 | isovaleryl-CoA-dehydrogenase (IVD) identical to isovaleryl-CoA-dehydrogenase precursor [Arabidopsis thaliana] GI:5596622 | At3g45300 | 113.734971 | 83.831 | 210.060747 | 132.539 | 308.877942 | 126.223 | 1.79623209 | 0.142662 |
| 24474 | 38 | UDP-glucoronosyl/UDP-glucosyl transferase family protein contains Pfam profile: PF00201 UDP-glucoronosyl and UDP-glucosyl transferase | At4g15260 | 215.860819 | 120.235 | 157.564283 | 97.867 | 190.50999 | 70.418 | 2.036908059 | 0.125097 |
| 23460 | 37 | 2-oxoisovalerate dehydrogenase, putative / 3-methyl-2-oxobutanoate dehydrogenase, putative / branched-chain alpha-keto acid dehydrogenase E1 alpha subunit, putative similar to branched-chain alpha-keto acid dehydrogenase E1-alpha subunit [Gallus gallus] GI:12964598; contains Pfam profile PF00676: Dehydroge | At1g21400 | 191.387965 | 160.939 | 158.963254 | 88.597 | 148.576967 | 32.607 | 2.513340448 | 0.12432 |
| 7632 | 12 | mannitol transporter, putative similar to mannitol transporter [Apium graveolens var. dulce] GI:12004316; contains Pfam profile PF00083: major facilitator superfamily protein | At4g36670 | 112.887871 | 98.146 | 108.195585 | 59.694 | 290.174375 | 164.606 | 1.575183012 | 0.12096 |
| 16385 | 26 | zinc finger (MYND type) family protein / F-box family protein | At1g67340 | 199.491035 | 167.752 | 224.560333 | 199.557 | 1523.11494 | 472.638 | 1.845692936 | 0.102524 |
| 11260 | 18 | expressed protein | At2g24100 | 148.071409 | 91.743 | 140.089892 | 81.536 | 354.648176 | 82.893 | 2.536833671 | 0.100674 |
| 29006 | 45 | expressed protein | At4g03565 | 304.951805 | 227.613 | 126.568631 | 70.46 | 129.768242 | 74.544 | 1.625642799 | 0.091008 |
| 27809 | 43 | expressed protein | At5g06980 | 145.872371 | 107.082 | 188.966241 | 126.804 | 179.74683 | 48.624 | 2.18304706 | 0.090801 |
| 5902 | 10 | plant defensin-fusion protein, putative (PDF1.4) plant defensin protein family member, personal communication, Bart Thomma (Bart.Thomma@agr.kuleuven.ac.be); similar to SWISS-PROT:P30224, Cysteine-rich antifungal protein 1 precursor (AFP1)[Arabidopsis thaliana] | At1g19610 | 127.988075 | 87.367 | 105.445444 | 61.466 | 237.60603 | 58.843 | 2.406140667 | 0.088972 |
| 11444 | 18 | expansin, putative (EXP17) similar to alpha-expansin precursor GI:4027891 from [Nicotiana tabacum]; alpha-expansin gene family, PMID:11641069 | At4g01630 | 154.683497 | 78.963 | 124.715552 | 65.441 | 208.434156 | 121.889 | 1.858246615 | 0.0861 |
| 3832 | 6 | disease resistance protein (TIR-NBS-LRR class), putative domain signature TIR-NBS-LRR exists, suggestive of a disease resistance protein. | At5g46450 | 133.379782 | 80.603 | 92.110606 | 69.425 | 116.553474 | 74.746 | 1.513621956 | 0.074777 |
| 4751 | 8 | dehydrin family protein contains Pfam domain, PF00257: Dehydrin | At2g21490 | 299.250992 | 271.422 | 173.591901 | 151.61 | 792.620116 | 308.184 | 1.606475201 | 0.072427 |
| 1342 | 3 | MATE efflux family protein similar to ripening regulated protein DDTFR18 [Lycopersicon esculentum] GI:12231296; contains Pfam profile PF01554: Uncharacterized membrane protein family | At1g15170 | 241.007503 | 149.997 | 242.603552 | 199.854 | 404.459749 | 198.136 | 1.620658873 | 0.065934 |
| 28034 | 44 | thioesterase family protein contains Pfam profile PF03061: thioesterase family protein | At1g35290 | 255.519442 | 156.529 | 340.381744 | 204.181 | 216.158063 | 113.586 | 1.734167612 | 0.063375 |
| 24694 | 38 | expressed protein | At1g36640 | 196.970056 | 87.243 | 91.2501911 | 72.034 | 121.776286 | 91.646 | 1.617750441 | 0.06048 |
| 1896 | 3 | expressed protein similar to unknown protein (gb\|AAB68039.1) | At5g51390 | 353.015117 | 158.742 | 217.624432 | 160.463 | 507.408754 | 385.188 | 1.632453006 | 0.060291 |
| 25229 | 39 | myb family transcription factor (MYB9) contains Pfam profile: PF00249 Myb-like DNA-binding domain | At5g16770 | 195.125046 | 154.436 | 220.057494 | 178.994 | 832.864437 | 378.07 | 1.565272822 | 0.058464 |
| 3426 | 6 | pentatricopeptide (PPR) repeat-containing protein contains Pfam profile PF01535: PPR repeat | At2g01860 | 113.957442 | 73.512 | 83.3216268 | 41.731 | 230.556367 | 110.876 | 1.875410621 | 0.0539 |
| 25123 | 39 | xyloglucan:xyloglucosyl transferase, putative / xyloglucan endotransglycosylase, putative / endo-xyloglucan transferase, putative similar to endoxyloglucan transferase EXGT-A1 GI:5533309 from [Arabidopsis thaliana] | At4g13090 | 136.112536 | 86.106 | 193.11376 | 83.214 | 234.746164 | 45.624 | 3.01555935 | 0.04884 |
| 11537 | 18 | esterase, putative similar to ethylene-induced esterase [Citrus sinensis] GI:14279437, polyneuridine aldehyde esterase [Rauvolfia serpentina] GI:6651393; contains Pfam profile PF00561: hydrolase, alpha/beta fold family | At4g37150 | 230.99311 | 124.195 | 170.811486 | 109.248 | 136.964301 | 48.912 | 2.074554014 | 0.048384 |
| 733 | 2 | phosphate-responsive protein, putative similar to phi-1 (phosphate-induced gene) [Nicotiana tabacum] GI:3759184; contains Pfam profile PF04674: Phosphate-induced protein 1 conserved region | At1g35140 | 328.59895 | 160.937 | 267.107851 | 166.861 | 402.206084 | 240.503 | 1.771640179 | 0.04424 |
| 1233 | 2 | CBL-interacting protein kinase 20 (CIPK20) identical to CBL-interacting protein kinase 20 [Arabidopsis thaliana] gi\|14486384\|gb\|AAK61493 | At5g45820 | 319.119343 | 218.521 | 262.24332 | 167.245 | 373.054829 | 175.6 | 1.717612329 | 0.038808 |
| 12203 | 19 | expressed protein | At5g03930 | 169.913119 | 100.831 | 206.545793 | 83.648 | 338.717875 | 214.241 | 1.911788938 | 0.03663 |
| 22091 | 34 | hypothetical protein | At1g34560 | 171.800911 | 142.419 | 219.941179 | 187.243 | 163.849519 | 68.487 | 1.591117863 | 0.033288 |

| | | | | | | | | | | | |
|---|---|---|---|---|---|---|---|---|---|---|---|
| 4042 | 7 | expressed protein | At1g68945 | 261.445935 | 157.519 | 307.13786 | 194.681 | 227.20989 | 99.16 | 1.84292233 | 0.0285 |
| 6180 | 10 | leucine-rich repeat family protein | At3g26500 | 154.578679 | 101.428 | 96.5337762 | 61.341 | 287.310385 | 139.566 | 1.71878199 | 0.027885 |
| 17618 | 28 | expressed protein | At1g27510 | 142.068977 | 124.584 | 135.950341 | 101.604 | 251.422876 | 122.879 | 1.508163122 | 0.027495 |
| 16921 | 27 | leucine-rich repeat protein kinase, putative similar to light repressible receptor protein kinase, gi\|2129635; contains leucine rich repeat (LRR) domains, Pfam:PF00560; contains protein kinase domain, Pfam:PF00069 | At1g05700 | 192.703537 | 130.074 | 199.621046 | 154.116 | 179.039595 | 102.65 | 1.506977269 | 0.025872 |
| 1133 | 2 | G-box binding factor 1 (GBF1) identical to G-box binding factor 1 SP:P42774 from [Arabidopsis thaliana]; contains Pfam profile: PF00170 bZIP transcription factor | At4g36730 | 200.358457 | 152.574 | 212.410955 | 189.838 | 1335.41121 | 607.551 | 1.543372789 | 0.02432 |
| 30386 | 47 | expressed protein | At4g35430 | 305.476965 | 263.299 | 201.799171 | 104.451 | 147.407865 | 81.415 | 1.634254204 | 0.0243 |
| 1227 | 2 | disease resistance protein (TIR-NBS-LRR class), putative (RPS4) domain signature TIR-NBS-LRR exists, suggestive of a disease resistance protein. Identical to RPS4 (GI:11357255). False intron created at intron 2 to escape a frameshift in the BAC sequence. | At5g45250 | 220.251408 | 113.827 | 171.179552 | 146.234 | 358.287444 | 171.268 | 1.732507467 | 0.02405 |
| 28208 | 44 | expressed protein | At2g44010 | 215.492887 | 119.051 | 180.546922 | 139.018 | 133.867315 | 95.045 | 1.505760614 | 0.023667 |
| 30325 | 47 | UDP-glucoronosyl/UDP-glucosyl transferase family protein contains Pfam profile: PF00201 UDP-glucoronosyl and UDP-glucosyl transferase ;simalr to UDP-glucose:sinapate glucosyltransferase GI:9794913 from [Brassica napus] | At4g15490 | 191.631827 | 169.39 | 118.230573 | 86.184 | 512.262485 | 133.271 | 2.11563668 | 0.023188 |
| 4437 | 7 | protein kinase family protein contains protein kinase domain, Pfam:PF00069 | At5g20050 | 238.968901 | 130.655 | 181.415303 | 139.823 | 325.610326 | 156.765 | 1.734510329 | 0.022914 |
| 1563 | 3 | UDP-glucoronosyl/UDP-glucosyl transferase family protein contains Pfam profile: PF00201 UDP-glucoronosyl and UDP-glucosyl transferase | At2g43840 | 204.050616 | 123.849 | 149.302706 | 130.509 | 288.320719 | 130.916 | 1.664637551 | 0.02128 |
| 28594 | 44 | thioesterase family protein contains Pfam profile PF03061: thioesterase family protein | At1g35290 | 307.70809 | 191.945 | 209.687901 | 132.043 | 119.522621 | 70.289 | 1.630526173 | 0.019684 |
| 27408 | 43 | GDSL-motif lipase/hydrolase family protein similar to lipase GI:1145627 from (Arabidopsis thaliana); contains Pfam profile PF00657: Lipase/Acylhydrolase with GDSL-like motif | At1g53940 | 280.260759 | 133.49 | 145.134473 | 108.856 | 164.837173 | 61.815 | 2.033126681 | 0.019536 |
| 21214 | 33 | zinc finger (C3HC4-type RING finger) family protein zinc finger protein, Arabidopsis thaliana, gb:L76926 | At4g10160 | 511.843202 | 400.304 | 317.657229 | 273.187 | 212.320852 | 64.378 | 1.913151218 | 0.01887 |
| 780 | 2 | MATE efflux family protein contains TIGRfam profile: TIGR00797: MATE efflux family protein, Pfam profile PF01554: Uncharacterized membrane protein family | At1g66760 | 281.051172 | 143.285 | 164.868249 | 141.866 | 236.370947 | 143.964 | 1.588499873 | 0.015708 |
| 11139 | 18 | expressed protein similar to putative non-LTR retroelement reverse transcriptase GB:AAD21515 GI:4510429 from [Arabidopsis thaliana]; expression supported by MPSS | At1g47320 | 129.178507 | 92.626 | 91.4907886 | 60.552 | 119.007144 | 59.386 | 1.63651002 | 0.014664 |
| 8446 | 13 | MATE efflux family protein similar to ripening regulated protein DDTFR18 [Lycopersicon esculentum] GI:12231296; contains Pfam profile PF01554: Uncharacterized membrane protein family | At1g33090 | 152.404241 | 130.642 | 199.527038 | 179.303 | 173.979673 | 60.843 | 1.712952395 | 0.01386 |
| 5486 | 9 | expressed protein identical to unknown protein GB:AAF30301 from [Arabidopsis thaliana] | At3g06080 | 274.671181 | 162.311 | 109.130888 | 53.519 | 316.193592 | 209.634 | 1.746556824 | 0.01326 |
| 26698 | 42 | myb family transcription factor (MYB58) contains PFAM profile: myb DNA binding domain PF00249 | At1g16490 | 88.8514021 | 71.223 | 106.635685 | 63.672 | 345.408766 | 192.774 | 1.571352196 | 0.012546 |
| 4922 | 8 | expressed protein | At3g56290 | 128.103588 | 106.125 | 149.549677 | 100.546 | 208.526942 | 100.038 | 1.592984654 | 0.010759 |
| 19234 | 30 | glycosyl hydrolase family 1 protein contains Pfam PF00232 : Glycosyl hydrolase family 1 domain; TIGRFAM TIGR01233: 6-phospho-beta-galactosidase; similar to Cyanogenic Beta-Glucosidase (GI:1311386)(pdb:1CBG) [Trifolium Repens]; identical beta-glucosidase GI:10834547 | At3g60140 | 163.883947 | 109.178 | 270.11293 | 182.171 | 375.652833 | 15.071 | 9.303118767 | 0.010248 |
| 5901 | 10 | expressed protein | At1g19530 | 132.099505 | 61.514 | 58.1736101 | 40.551 | 420.629232 | 72.298 | 3.133347384 | 0.01005 |
| 9540 | 15 | hypothetical protein | At4g19280 | 86.6159987 | 58.634 | 97.5152866 | 63.312 | 248.216609 | 95.777 | 1.869691751 | 0.009776 |
| 16714 | 26 | expressed protein | At4g30790 | 137.501909 | 84.735 | 152.744366 | 135.939 | 117.297822 | 43.269 | 1.819083461 | 0.008526 |
| 22152 | 35 | phytochrome kinase substrate-related contains weak similarity to Swiss-Prot:Q9SWI1 phytochrome kinase substrate 1 [Arabidopsis thaliana] | At1g18810 | 183.998299 | 142.503 | 1008.25175 | 56.505 | 78.2555943 | 64.938 | 6.779951779 | 0.008432 |
| 23935 | 37 | myb family transcription factor similar to CCA1 [Arabidopsis thaliana] GI:4090569; contains Pfam profile PF00249: Myb-like DNA-binding domain | At5g17300 | 96.4047125 | 65.593 | 198.014938 | 89.288 | 110.21723 | 54.052 | 1.908849215 | 0.008424 |
| 30576 | 48 | branched-chain amino acid aminotransferase 2 / branched-chain amino acid transaminase 2 (BCAT2) identical to SP\|Q9M439 Branched-chain amino acid aminotransferase 2, chloroplast precursor (EC 2.6.1.42) (Atbcat-2) {Arabidopsis thaliana}; contains Pfam profile: PF01063 aminotransferase class IV | At1g10070 | 151.817047 | 36.508 | 146.573278 | 33.886 | 1110.14776 | 71.997 | 7.967767654 | 0.008424 |
| 11820 | 19 | expressed protein | At1g59590 | 187.83485 | 94.422 | 135.980614 | 98.473 | 185.373777 | 158.312 | 1.513714745 | 0.00774 |
| 256 | 1 | nucleotidyltransferase family protein contains Pfam profile: PF01909 nucleotidyltransferase domain | At2g40520 | 100.034836 | 43.817 | 100.980847 | 91.272 | 89.3239165 | 41.571 | 1.846031459 | 0.00663 |
| 2792 | 5 | expressed protein | At2g07676 | 120.879706 | 69.933 | 69.9804151 | 57.436 | 329.165055 | 185.317 | 1.574380394 | 0.006432 |
| 10360 | 16 | haloacid dehalogenase-like hydrolase family protein low similarity to SP\|P53078 SSM1 protein {Saccharomyces cerevisiae}; contains InterPro accession IPR005834: Haloacid dehalogenase-like hydrolase | At5g59490 | 60.9783819 | 43.231 | 79.9978757 | 60.66 | 163.723743 | 73.397 | 1.653325178 | 0.00598 |
| 17478 | 27 | expressed protein | At5g44575 | 199.579809 | 70.997 | 138.093411 | 83.727 | 163.888695 | 31.875 | 3.200679215 | 0.005883 |
| 22503 | 35 | expressed protein contains Pfam profile PF04520: Protein of unknown function, DUF584 | At4g04630 | 69.4889583 | 51.703 | 76.4717678 | 62.846 | 845.693628 | 377.844 | 1.599674297 | 0.005625 |
| 17127 | 27 | hypothetical protein | At2g30430 | 98.0304604 | 81.705 | 109.309345 | 68.07 | 617.056898 | 257.673 | 1.733458562 | 0.00546 |
| 18014 | 28 | protease inhibitor/seed storage/lipid transfer protein (LTP) family protein contains Pfam protease inhibitor/seed storage/LTP family domain PF00234 | At4g30880 | 379.733001 | 254.416 | 410.695171 | 268.073 | 156.968932 | 44.475 | 2.184656396 | 0.005328 |
| 14449 | 23 | MADS-box protein (AGL60) contains Pfam profile: PF00319 SRF-type transcription factor (DNA-binding and dimerisation domain) | At1g72350 | 87.2716457 | 26.72 | 204.158938 | 142.159 | 1116.83041 | 429.967 | 2.433254901 | 0.004648 |
| 17030 | 27 | expressed protein contains Pfam profile PF03140: Plant protein of unknown function | At1g65985 | 110.050299 | 65.886 | 146.372515 | 75.835 | 334.416735 | 138.344 | 2.005914144 | 0.0043 |
| 28 | 1 | L-allo-threonine aldolase-related similar to L-allo-threonine aldolase (EC 4.1.2.-) (L-allo-TA) (L-allo-threonine acetaldehyde-lyase) (SP:O07051) {Aeromonas jandaei}; similar to ESTs gb\|R30517, gb\|T42772, gb\|R90493, and gb\|R90493 | At1g08630 | 99.8883053 | 30.275 | 112.623536 | 59.297 | 640.486358 | 41.41 | 6.888542509 | 0.00414 |
| 25885 | 40 | hydrolase, alpha/beta fold family protein low similarity to hydrolase [Terrabacter sp. DBF63] GI:14196240; contains Pfam profile PF00561: hydrolase, alpha/beta fold family | At5g19850 | 122.308653 | 107.091 | 251.972515 | 215.736 | 192.209002 | 46.647 | 2.143522653 | 0.00363 |
| 18271 | 29 | phytochrome and flowering time regulatory protein (PFT1) PMID: 12815435 | At1g25540 | 228.661445 | 165.151 | 102.955021 | 59.584 | 1495.5926 | 928.896 | 1.574177453 | 0.003621 |
| 18169 | 28 | peptidoglycan-binding LysM domain-containing protein contains Pfam profile PF01476: LysM domain | At5g62150 | 164.739604 | 121.688 | 110.201627 | 83.868 | 177.480793 | 94.295 | 1.516654138 | 0.003528 |
| 23501 | 37 | F-box family protein contains F-box domain Pfam:PF00646 | At1g49610 | 108.350751 | 90.053 | 183.023005 | 136.61 | 104.718122 | 9.024 | 4.715779561 | 0.002714 |
| 23064 | 36 | hypothetical protein | At3g19610 | 162.325582 | 137.48 | 168.856432 | 99.025 | 148.385209 | 90.139 | 1.51069782 | 0.002695 |
| 1004 | 2 | cytochrome P450 71A21, putative (CYP71A21) identical to Cytochrome P450 71A21 (SP:Q9STL2) [Arabidopsis thaliana] | At3g48320 | 150.635171 | 89.772 | 89.7110044 | 48.133 | 85.4166008 | 71.3 | 1.57992624 | 0.00198 |
| 9706 | 15 | expressed protein similar to unknown protein (pir\|\|T05871) | At5g54150 | 70.0365787 | 49.577 | 72.3991371 | 34.368 | 530.61759 | 301.602 | 1.759533013 | 0.00196 |
| 1293 | 2 | phosphate-responsive protein, putative similar to phi-1 (phosphate-induced gene) [Nicotiana tabacum] GI:3759184; contains Pfam profile PF04674: Phosphate-induced protein 1 conserved region | At1g35140 | 133.531661 | 52.713 | 83.4411288 | 48.618 | 135.613751 | 87.895 | 1.930783017 | 0.001848 |
| 15370 | 24 | expressed protein | At4g14990 | 144.702261 | 129.615 | 135.889793 | 115.168 | 2097.92953 | 744.454 | 1.704801785 | 0.00182 |
| 2184 | 4 | nodulin-related contains 14 transmembrane domains; supported by tandem duplication of nodulin -related protein (TIGR_Ath1:At2g34350) [Arabidopsis thaliana] | At2g34355 | 99.2112668 | 65.204 | 75.7611288 | 53.593 | 206.786691 | 94.714 | 1.706155061 | 0.00175 |
| 11003 | 17 | ankyrin repeat family protein contains Pfam domain, PF00023: Ankyrin repeat | At5g54620 | 101.544001 | 80.001 | 121.971785 | 38.877 | 88.4641008 | 77.814 | 1.847842245 | 0.001566 |
| 14867 | 23 | hypothetical protein | At5g37385 | 80.7108972 | 30.091 | 150.008565 | 113.445 | 178.159161 | 105.543 | 1.897517897 | 0.001558 |
| 16698 | 26 | RNA recognition motif (RRM)-containing protein low similarity to tumor-rejection antigen SART3 [Mus musculus] GI:7637845; contains INTERPRO:IPR000504 RNA-binding region RNP-1 (RNA recognition motif) domain | At4g24270 | 115.726513 | 102.82 | 112.092946 | 26.084 | 180.008692 | 124.476 | 2.289680116 | 0.001458 |
| 13939 | 22 | transcription elongation factor-related low similarity to transcription elongation factor TFIIS.h [Mus musculus] GI:3288547 | At3g10820 | 122.163191 | 101.036 | 155.478573 | 120.106 | 139.832415 | 64.49 | 1.55729915 | 0.001404 |
| 22649 | 35 | ACT domain-containing protein contains Pfam ACT domain PF01842 | At5g25320 | 81.2734916 | 45.574 | 71.2901579 | 47.316 | 224.675805 | 66.762 | 2.218445575 | 0.00126 |
| 551 | 1 | protein kinase family protein contains protein kinase domain, Pfam:PF00069 | At5g25440 | 99.5481817 | 33.989 | 72.9584068 | 38.421 | 141.704627 | 107.807 | 2.047394705 | 0.000936 |
| 19615 | 31 | pseudouridine synthase family protein low similarity to SP\|P23851 Ribosomal large subunit pseudouridine synthase C (EC 4.2.1.70) (Pseudouridylate synthase) (Uracil hydrolyase) {Escherichia coli}; contains Pfam profile PF00849: RNA pseudouridylate synthase | At1g56345 | 113.069698 | 81.624 | 179.350308 | 123.085 | 683.66065 | 334.492 | 1.628751232 | 0.000864 |
| 17870 | 28 | lipid-binding serum glycoprotein family protein similar to SP\|P17213 Bactericidal permeability-increasing protein precursor (BPI) {Homo sapiens}; contains Pfam profile PF02886: LBP / BPI / CETP family, C-terminal domain | At3g20270 | 135.442984 | 102.454 | 98.691187 | 89.521 | 87.2826275 | 40.865 | 1.520100583 | 0.00084 |
| 9014 | 14 | peroxidase, putative identical to peroxidase ATP24a [Arabidopsis thaliana] gi\|1890313\|emb\|CAA72484 | At5g39580 | 119.791952 | 76.441 | 122.966042 | 49.449 | 693.935137 | 74.349 | 4.462441389 | 0.000732 |
| 115 | 1 | carbonic anhydrase family protein / carbonate dehydratase family protein similar to SP\|P46512 Carbonic anhydrase 1 (EC 4.2.1.1) (Carbonate dehydratase 1) {Flaveria linearis}; contains Pfam profile PF00484: Carbonic anhydrase | At1g58180 | 110.404327 | 43.946 | 54.5439338 | 34.952 | 74.5462218 | 67.533 | 1.725553196 | 0.000696 |
| 20589 | 32 | MATE efflux family protein similar to ripening regulated protein DDTFR18 [Lycopersicon esculentum] GI:12231296; contains Pfam profile PF01554: MatE | At4g21900 | 81.3152048 | 60.092 | 110.396017 | 92.295 | 57.5416666 | 15.03 | 2.125918017 | 0.000666 |
| 20476 | 32 | glycosyl hydrolase family 17 protein similar to elicitor inducible chitinase Nt-SubE76 GI:11071974 from [Nicotiana tabacum] | At3g24330 | 74.7769173 | 65.827 | 76.2662243 | 29.178 | 3264.11583 | 1467.62 | 1.991291791 | 0.00049 |
| 13547 | 21 | squalene monooxygenase 1,2 / squalene epoxidase 1,2 (SQP1,2) identical to SP\|O65402 | At5g24160 | 85.3688789 | 68.612 | 143.652648 | 91.83 | 118.380324 | 35.848 | 2.036948134 | 0.000392 |
| 27058 | 42 | hypothetical protein | At4g07350 | 52.8464335 | 47.901 | 94.334938 | 74.259 | 117.114312 | 29.066 | 2.134282526 | 0.000344 |
| 21221 | 33 | late embryogenesis abundant domain-containing protein / LEA domain-containing protein similar to LEA protein [Cicer arietinum] GI:2909420; contains Pfam profile PF02987: Late embryogenesis abundant protein | At4g13560 | 141.277494 | 116.876 | 176.856698 | 100.908 | 186.464526 | 86.502 | 1.70568112 | 0.000324 |
| 17540 | 27 | miRNA gene Arabidopsis thaliana miR399f stem-loop | ath-MIR39 | 121.66691 | 85.14 | 59.9470209 | 36.624 | 1690.18929 | 705.068 | 1.821015181 | 0.00028 |
| 17141 | 27 | UDP-glucoronosyl/UDP-glucosyl transferase family protein contains Pfam profile: PF00201 UDP-glucoronosyl and UDP-glucosyl transferase | At2g36750 | 81.9687127 | 44.929 | 86.981577 | 47.228 | 55.6467731 | 32.2 | 1.798101192 | 0.00027 |
| 2054 | 4 | protein kinase family protein contains Serine/Threonine protein kinases active-site signature, PROSITE:PS00108 | At1g51910 | 95.9490753 | 40.183 | 88.4171953 | 27.86 | 102.532499 | 85.772 | 2.252278455 | 0.000232 |
| 1275 | 2 | ethylene insensitive 3 family protein contains Pfam profile: PF04873 ethylene insensitive 3 | At5g65100 | 80.7964629 | 37.869 | 70.6783072 | 48.114 | 129.500194 | 76.21 | 1.767269488 | 0.000225 |
| 10676 | 17 | acyl-[acyl-carrier-protein] desaturase, putative / stearoyl-ACP desaturase, putative similar to Acyl-[acyl-carrier protein] desaturase from Spinacia oleracea SP\|P28645, Cucumis sativus SP\|P32061, Ricinus communis SP\|P22337; contains Pfam profile PF03405 Fatty acid desaturase | At3g02610 | 70.1788316 | 59.547 | 68.5527637 | 54.506 | 434.017201 | 140.243 | 1.843668959 | 0.000224 |
| 7726 | 12 | hypothetical protein | At5g42785 | 127.440454 | 58.591 | 54.8880997 | 29.522 | 98.860241 | 81.544 | 1.748889138 | 0.000192 |
| 13987 | 22 | hypothetical protein contains Pfam profile PF03384: Drosophila protein of unknown function, DUF287 | At3g32080 | 65.4342146 | 37.186 | 94.1023073 | 65.911 | 2912.02854 | 1843.019 | 1.589132014 | 0.00018 |
| 8030 | 13 | UDP-glucoronosyl/UDP-glucosyl transferase family protein contains Pfam profile: PF00201 UDP-glucoronosyl and UDP-glucosyl transferase | At2g29750 | 106.729281 | 94.701 | 61.333245 | 42.613 | 864.017805 | 290.768 | 1.845941312 | 0.000161 |
| 20732 | 32 | ABC transporter family protein contains Pfam profile: PF00005 ABC transporter; similar to multidrug-resistant protein CjMDR1 GI:14715462 from [Coptis japonica] | At5g46540 | 136.913645 | 110.466 | 59.9996018 | 43.236 | 222.857202 | 107.103 | 1.569305569 | 0.000144 |
| 1286 | 2 | miRNA gene Arabidopsis thaliana miR395e stem-loop | ath-MIR39 | 76.3053343 | 67.54 | 41.2584897 | 27.133 | 820.95898 | 334.772 | 1.700891527 | 0.00014 |
| 2949 | 5 | hypothetical protein predicted protein, Arabidopsis thaliana | At3g43280 | 90.3637755 | 48.101 | 95.4263903 | 58.303 | 179.122072 | 50.89 | 2.345048993 | 0.000124 |
| 20365 | 32 | expressed protein | At2g25770 | 347.593462 | 132.349 | 55.1525977 | 11.59 | 217.052931 | 142.191 | 2.970488336 | 0.00012 |
| 29620 | 46 | yippee family protein similar to mdgl-1 [Mus musculus] GI:10441648, Yippee protein [Drosophila melanogaster] GI:5713279; contains Pfam profile PF03226: Yippee putative zinc-binding protein | At3g55890 | 48.3146612 | 25.743 | 94.9850293 | 74.616 | 133.644629 | 94.732 | 1.520185958 | 0.000117 |
| 1873 | 3 | hypothetical protein similar to unknown protein (emb\|CAB86921.1) | At5g41660 | 89.9616168 | 76.477 | 106.253278 | 89.41 | 111.660566 | 40.836 | 1.6996903 | 0.000108 |
| 2183 | 4 | expressed protein | At2g34310 | 53.907448 | 44.334 | 65.7404815 | 48.754 | 112.145114 | 55.649 | 1.526524566 | 0.000081 |
| 4772 | 8 | leucine-rich repeat protein kinase, putative similar to light repressible receptor protein kinase [Arabidopsis thaliana] gi\|1321686\|emb\|CAA66376; contains leucine rich repeat (LRR) domains, Pfam:PF00560; contains protein kinase domain, Pfam:PF00069 | At2g28970 | 77.3374703 | 50.637 | 66.1595354 | 36.059 | 136.591095 | 110.51 | 1.532685425 | 0.00008 |
| 14839 | 23 | floral homeotic protein PISTILLATA (PI) contains Pfam profiles PF01486: K-box region and PF00319: SRF-type transcription factor (DNA-binding and dimerisation domain) | At5g20240 | 72.5821576 | 55.676 | 84.8353197 | 30.103 | 226.306774 | 41.153 | 3.206992328 | 0.000074 |
| 20497 | 32 | receptor protein kinase-related similar to light repressible receptor protein kinase GI:1321686 from [Arabidopsis thaliana] | At3g45920 | 98.8465431 | 69.737 | 88.9461911 | 54.117 | 98.7055979 | 59.79 | 1.570626947 | 0.000065 |
| 16559 | 26 | F-box family protein contains F-box domain Pfam:PF00646 | At3g16580 | 62.4009113 | 28.433 | 51.1500748 | 12.322 | 2816.3622 | 1283.31 | 2.846796934 | 0.000042 |
| 29479 | 46 | expressed protein | At2g32160 | 57.0509171 | 44.185 | 192.640532 | 132.202 | 68.8512319 | 39.262 | 1.500662115 | 0.000028 |
| 11179 | 18 | S-adenosyl-L-methionine:carboxyl methyltransferase family protein similar to defense-related protein cjs1 [Brassica carinata][GI:14009292][Mol Plant Pathol (2001) 2(3):159-169] | At1g66690 | 69.8173166 | 46.627 | 42.2734607 | 26.566 | 2516.05767 | 578.303 | 2.479793363 | 0.000027 |
| 26303 | 41 | auxin-responsive protein-related similar to indole-3-acetic acid induced protein arg7 (SP:P32295) [Vigna radiata] | At3g12955 | 65.1518479 | 52.375 | 95.4040832 | 73.537 | 102.77168 | 52.147 | 1.504039386 | 0.000025 |
| 25920 | 40 | hypothetical protein | At5g42905 | 57.876626 | 46.017 | 128.628847 | 58.951 | 46.0650866 | 29.852 | 1.660933462 | 0.00002 |
| 14814 | 23 | expressed protein ; expression supported by MPSS | At5g09580 | 122.102225 | 79.596 | 80.1476517 | 61.745 | 129.63628 | 66.866 | 1.590271589 | 0.000012 |
| 11374 | 18 | expressed protein contains Pfam domain PF05003: protein of unknown function (DUF668); expression supported by MPSS | At3g23160 | 61.960248 | 48.348 | 55.2195188 | 23.902 | 3.36091004 | 1.611 | 1.892673349 | 0.000012 |
| 28142 | 44 | hypothetical protein | At2g13980 | 185.621908 | 154.873 | 166.944399 | 139.668 | 81.3711373 | 33.034 | 1.619030472 | 0.00001 |
| 20277 | 32 | MATE efflux family protein similar to ripening regulated protein DDTFR18 [Lycopersicon esculentum] GI:12231296; contains Pfam profile PF01554: Uncharacterized membrane protein family; contains 12 transmembrane domains, PMID: 11152613 | At1g64820 | 68.4439874 | 59.822 | 61.9976765 | 43.642 | 51.772448 | 8.882 | 2.79788059 | 0.000008 |
| 22551 | 35 | F-box family protein contains F-box domain Pfam:PF00646 | At4g26350 | 52.9533906 | 37.243 | 59.8307056 | 31.571 | 604.192654 | 257.838 | 1.886751346 | 0.000006 |
| 9856 | 16 | DC1 domain-containing protein contains Pfam profile PF03107: DC1 domain | At1g55430 | 31.5694576 | 19.216 | 47.5188051 | 39.071 | 56.5375174 | 34.283 | 1.502743922 | 0.000005 |
| 10600 | 17 | expressed protein | At2g16980 | 89.739146 | 58.407 | 36.8719669 | 7.457 | 38.1576694 | 18.015 | 2.866387282 | 0.000004 |
| 9016 | 14 | no apical meristem (NAM) family protein contains Pfam PF02365: No apical meristem (NAM) domain; NAC domain protein NAM, Arabidopsis thaliana, gb:AAD17313 | At5g39820 | 99.2433539 | 39.57 | 69.4673529 | 49.843 | 98.8416838 | 75.006 | 1.739850793 | 0.000003 |
| 16291 | 26 | expressed protein | At1g15800 | 65.183935 | 28.812 | 64.0148715 | 35.326 | 81.2969086 | 49.651 | 1.903957719 | 0.000002 |
| 23994 | 37 | ankyrin repeat family protein contains ankyrin repeat domains, Pfam:PF00023 | At5g50140 | 34.6872571 | 27.999 | 54.4674524 | 16.441 | 49.1600105 | 15.619 | 2.566409338 | 0.000002 |
| 16659 | 26 | subtilase family protein contains similarity to subtilase; SP1 GI:9957714 from [Oryza sativa] | At4g10540 | 59.3676079 | 20.535 | 46.8750873 | 19.15 | 205.931 | 66.868 | 2.806164862 | 0.000002 |
| 11156 | 18 | calcium-binding EF-hand family protein contains Pfam profile: PF00036 EF hand | At1g54450 | 48.6312542 | 44.04 | 51.0385396 | 42.671 | 32.9369184 | 12.569 | 1.640278193 | 0.000001 |
| 16364 | 26 | glutathione S-transferase, putative similar to glutathione S-transferase GB:AAF29773 GI:6856103 from [Gossypium hirsutum] | At1g59670 | 38.6607134 | 23.855 | 42.8152034 | 23.179 | 290.675418 | 74.934 | 2.448965084 | 0.000001 |
| 7458 | 12 | F-box family protein contains F-box domain Pfam:PF00646 | At3g16555 | 36.704468 | 7.255 | 40.5510375 | 31.783 | 27.3718287 | 14.059 | 2.760664596 | 0.000001 |

Downregulated genes

| SPOTNUMBER | BLOCK | NAME | AG ID | BLR-OX (1) | Col-0 (1) | BLR-OX (2) | Col-0 (2) | BLR-OX (3) | Col-0 (3) | Mean Ratio (BLR-OX:Col-0) | Spot Confidence |
|---|---|---|---|---|---|---|---|---|---|---|---|
| 24301 | 38 | expressed protein | At2g41650 | 1363.53617 | 2233.947 | 1010.22115 | 1466.435 | 480.42044 | 802.966 | 0.632524718 | 0.735816 |
| 24959 | 39 | cold-responsive protein / cold-regulated protein (cor15b) nearly identical to cold-regulated gene cor15b [Arabidopsis thaliana] GI:456016; contains Pfam profile PF02987: Late embryogenesis abundant protein | At2g42530 | 2061.47403 | 4424.512 | 4093.06398 | 10575.54 | 968.905003 | 1914.726 | 0.452993517 | 0.7209 |
| 10518 | 17 | disease resistance protein (CC-NBS-LRR class), putative domain signature CC-NBS-LRR exists, suggestive of a disease resistance protein. | At1g58400 | 2925.03817 | 3891.313 | 2128.00372 | 3204.954 | 1206.08628 | 2938.268 | 0.60871087 | 0.7209 |
| 5312 | 9 | histone H1/H5 family protein contains Pfam domain, PF00538: linker histone H1 and H5 family | At1g54260 | 1276.22923 | 1551.95 | 596.06679 | 1093.608 | 541.413741 | 892.766 | 0.657943517 | 0.720099 |
| 16389 | 26 | expressed protein | At1g70270 | 2132.43793 | 2698.786 | 1856.9061 | 3102.25 | 1132.4741 | 2351.006 | 0.623470762 | 0.711822 |
| 19576 | 31 | stress-responsive protein, putative similar to cold acclimation WCOR413-like protein gamma form [Hordeum vulgare] gi\|18449100\|gb\|AAL69988; similar to stress-regulated protein SAP1 [Xerophyta viscosa] gi\|21360378\|gb\|AAM47505 | At1g29390 | 1535.2836 | 2738.97 | 1428.4322 | 2345.119 | 948.735419 | 2095.259 | 0.54081428 | 0.703248 |
| 22111 | 35 | latex-abundant family protein (AMC1) / caspase family protein contains similarity to latex-abundant protein [Hevea brasiliensis] gb:AAD13216; contains Pfam profile PF00656: ICE-like protease (caspase) p20 domain | At1g02170 | 2066.03468 | 2770.291 | 1730.58125 | 2606.191 | 1013.52057 | 2285.376 | 0.617763485 | 0.701592 |
| 22271 | 35 | Pex2/Pex12 N-terminal domain-containing protein / zinc finger (C3HC4-type RING finger) family protein contains Pfam profiles PF00097: zinc finger C3HC4 type (RING finger), PF04757: Pex2/Pex12 amino terminal region | At1g79810 | 1165.21739 | 1725.365 | 1553.36605 | 2814.957 | 516.328568 | 710.648 | 0.651243838 | 0.6966 |
| 3133 | 5 | hypothetical protein | At5g17120 | 677.697299 | 904.233 | 395.794059 | 812.658 | 623.09004 | 1026.722 | 0.614460532 | 0.68904 |
| 21058 | 33 | aconitase C-terminal domain-containing protein contains Pfam profile PF00694: Aconitase C-terminal domain | At2g43100 | 2225.50665 | 2779.839 | 2610.70925 | 3969.016 | 819.315639 | 1589.865 | 0.657899118 | 0.68816 |
| 1668 | 3 | Em-like protein GEA1 (EM1) identical to SP\|Q07187 Em-like protein GEA1 (EM1) {Arabidopsis thaliana}; contains Pfam profile PF00477: Small hydrophilic plant seed protein | At3g51810 | 1657.25641 | 1847.908 | 998.133911 | 1799.548 | 695.122796 | 1631.467 | 0.625852926 | 0.679042 |
| 19285 | 30 | S-locus protein kinase, putative (ARK3) identical to PIR\|T05180\|T05180 S-receptor kinase ARK3 precursor - [Arabidopsis thaliana] | At4g21380 | 844.002754 | 1247.083 | 921.112434 | 1218.567 | 557.900757 | 1127.796 | 0.642453979 | 0.673552 |
| 27575 | 43 | expressed protein | At3g02640 | 1074.79051 | 1867.862 | 957.425131 | 1562.723 | 364.02161 | 1076.256 | 0.508768793 | 0.671416 |
| 28656 | 45 | thaumatin, putative identical to SP\|P50699 Thaumatin-like protein precursor {Arabidopsis thaliana}; strong similarity to pathogenesis-related group 5 protein GI:2749943 from [Brassica rapa] | At1g18250 | 510.016375 | 939.324 | 536.502176 | 1019.766 | 294.793049 | 822.453 | 0.475831928 | 0.667368 |
| 4191 | 7 | lipid transfer protein 6 (LTP6) identical to GI:8571927 | At3g08770 | 1498.4187 | 2576.997 | 1678.56279 | 2783.115 | 1248.55128 | 2914.676 | 0.537650029 | 0.666072 |
| 14559 | 23 | cold-responsive protein / cold-regulated protein (cor15a) identical to cold-regulated protein cor15a [Arabidopsis thaliana] GI:507149; contains Pfam profile PF02987: Late embryogenesis abundant protein | At2g42540 | 1005.2117 | 2953.547 | 2631.30663 | 8904.34 | 1403.39438 | 1746.545 | 0.479791626 | 0.663834 |
| 19759 | 31 | cold-responsive protein / cold-regulated protein (cor15a) identical to cold-regulated protein cor15a [Arabidopsis thaliana] GI:507149; contains Pfam profile PF02987: Late embryogenesis abundant protein | At2g42540 | 1394.15906 | 3544.164 | 3174.96778 | 9353.715 | 853.586612 | 1706.895 | 0.410960972 | 0.656656 |
| 14376 | 23 | stress-responsive protein, putative similar to cold acclimation WCOR413-like protein gamma form [Hordeum vulgare] gi\|18449100\|gb\|AAL69988; similar to stress-regulated protein SAP1 [Xerophyta viscosa] gi\|21360378\|gb\|AAM47505 | At1g29390 | 1216.7536 | 2028.488 | 1543.18766 | 2375.085 | 832.518036 | 1662.635 | 0.583431606 | 0.650848 |
| 29010 | 45 | peroxidase, putative similar to peroxidase isozyme [Armoracia rusticana] gi\|217932\|dbj\|BAA14143 | At4g08780 | 1457.84025 | 1878.123 | 864.326641 | 1416.724 | 712.826338 | 1620.493 | 0.60873085 | 0.65016 |
| 3976 | 7 | stress-responsive protein, putative similar to cold acclimation WCOR413-like protein gamma form [Hordeum vulgare] gi\|18449100\|gb\|AAL69988; similar to stress-regulated protein SAP1 [Xerophyta viscosa] gi\|21360378\|gb\|AAM47505 | At1g29395 | 1447.00442 | 2004.122 | 1753.26912 | 3030.243 | 1067.7405 | 2321.501 | 0.586846605 | 0.649152 |
| 25722 | 40 | copper homeostasis factor / copper chaperone (CCH) (ATX1) identical to gi:3168840 Pfam profile PF00403: Heavy-metal-associated domain | At3g56240 | 1524.86812 | 1967.272 | 1360.98683 | 2379.424 | 647.484474 | 1078.661 | 0.649122203 | 0.64881 |
| 9566 | 15 | plastocyanin-like domain-containing protein | At4g31840 | 1121.53504 | 1849.567 | 753.893977 | 1241.57 | 1138.38559 | 2488.893 | 0.5569912 | 0.64701 |
| 14762 | 23 | glycine-rich protein glycine-rich protein - Onobrychis viciifolia,PID:g2565429 | At4g29030 | 969.289093 | 1258.401 | 979.289231 | 1522.563 | 439.602909 | 917.88 | 0.630790709 | 0.64206 |
| 23685 | 37 | short-chain dehydrogenase/reductase (SDR) family protein contains INTERPRO family IPR002198 short-chain dehydrogenase/reductase (SDR) superfamily | At3g05260 | 511.351199 | 747.055 | 753.573712 | 1052.782 | 322.981393 | 573.094 | 0.654618999 | 0.640872 |

| | | | | | | | | | | | |
|---|---|---|---|---|---|---|---|---|---|---|---|
| 18395 | 29 | xyloglucan fucosyltransferase, putative (FUT4) identical to SP\|Q9SJP2 Probable fucosyltransferase 4 (EC 2.4.1.-) (AtFUT4) {Arabidopsis thaliana}; similar to SP\|Q9SWH5 Galactoside 2-alpha-L-fucosyltransferase (EC 2.4.1.69) (Xyloglucan alpha-(1,2)-fucosyltransferase) (AtFUT1) {Arabidopsis thaliana} | At2g15390 | 1133.21368 | 1555.81 | 1075.49637 | 1449.85 | 481.521499 | 1120.857 | 0.633258309 | 0.635008 |
| 17421 | 27 | 3-isopropylmalate dehydrogenase, chloroplast, putative strong similarity to SP\|P29102 3-isopropylmalate dehydrogenase, chloroplast precursor {Brassica napus} | At5g14200 | 1476.25291 | 1960.594 | 1084.7777 | 1594.785 | 505.536542 | 1272.971 | 0.610098799 | 0.63495 |
| 23354 | 36 | pectinesterase family protein contains Pfam profile: PF01095 pectinesterase | At5g55590 | 1619.49949 | 1921.911 | 364.020847 | 651.356 | 566.655618 | 1229.065 | 0.620854301 | 0.6336 |
| 18990 | 30 | AP2 domain-containing transcription factor, putative | At1g71130 | 1914.41979 | 2321.514 | 642.796881 | 1003.692 | 752.829416 | 1481.759 | 0.657713293 | 0.63063 |
| 29019 | 45 | protease inhibitor/seed storage/lipid transfer protein (LTP) family protein similar to pEARLI 1 (Accession No. L43080): an Arabidopsis member of a conserved gene family (PGF95-099), Plant Physiol. 109 (4), 1497 (1995); contains Pfam protease inhibitor/seed storage/LTP family domain PF00234 | At4g12500 | 1375.60842 | 2216.844 | 1760.79297 | 3688.234 | 1304.93827 | 1798.645 | 0.607815197 | 0.62118 |
| 18490 | 29 | expressed protein contains Pfam profile PF04862: Protein of unknown function, DUF642 | At3g08030 | 1116.28772 | 1547.955 | 857.604251 | 1180.465 | 421.532348 | 974.096 | 0.626792046 | 0.61776 |
| 6153 | 10 | seven in absentia (SINA) family protein low similarity to SP\|P21461 Developmental protein seven in absentia {Drosophila melanogaster}; contains Pfam profile PF03145: Seven in absentia protein family | At3g13672 | 465.840953 | 544.613 | 482.844151 | 910.893 | 250.61667 | 444.479 | 0.649760963 | 0.61776 |
| 6624 | 11 | aspartate aminotransferase, cytoplasmic isozyme 2 / transaminase A (ASP4) identical to aspartate aminotransferase, cytoplasmic isozyme 2 SP:P46646 [Arabidopsis thaliana] | At1g62800 | 478.476865 | 558.403 | 533.006342 | 1020.786 | 310.781083 | 644.018 | 0.620528426 | 0.613008 |
| 4419 | 7 | sulfate transporter identical to sulfate transporter [Arabidopsis thaliana] GI:2114104 | At5g10180 | 492.847621 | 695.116 | 385.776599 | 847.532 | 694.877429 | 1355.84 | 0.558899441 | 0.612 |
| 17251 | 27 | 60S acidic ribosomal protein P2 (RPP2D) acidic ribosomal protein P2, maize, PIR:S54179 | At3g44590 | 794.930837 | 1208.689 | 387.56435 | 617.719 | 710.500506 | 1235.704 | 0.620022855 | 0.604824 |
| 11400 | 18 | expressed protein | At3g45160 | 587.668299 | 787.865 | 177.310805 | 340.514 | 356.235845 | 989.931 | 0.54215806 | 0.601524 |
| 5042 | 8 | lipid transfer protein, putative similar to lipid transfer protein 6 from Arabidopsis thaliana [gi:8571927]; contains Pfam protease inhibitor/seed storage/LTP family domain PF00234 | At5g01870 | 313.114771 | 523.68 | 384.653279 | 689.26 | 383.778812 | 703.904 | 0.567064714 | 0.598752 |
| 26134 | 41 | glycosyl hydrolase family 1 protein contains Pfam PF00232 : Glycosyl hydrolase family 1 domain; TIGRFAM TIGR01233: 6-phospho-beta-galactosidase; similar to beta-glucosidase 1 (GI:12043529) [Arabidopsis thaliana] | At1g66280 | 697.145308 | 840.614 | 303.892183 | 494.949 | 321.599915 | 670.335 | 0.621405182 | 0.598347 |
| 11824 | 19 | aspartate aminotransferase, cytoplasmic isozyme 2 / transaminase A (ASP4) identical to aspartate aminotransferase, cytoplasmic isozyme 2 SP:P46646 [Arabidopsis thaliana] | At1g62800 | 596.458033 | 827.563 | 840.932915 | 1758.586 | 299.875652 | 752.502 | 0.532477366 | 0.595056 |
| 9572 | 15 | expressed protein contains Pfam profile PF04862: Protein of unknown function, DUF642 | At4g32460 | 342.853123 | 539.991 | 398.665296 | 651.109 | 513.540868 | 721.42 | 0.603619209 | 0.59211 |
| 23669 | 37 | expressed protein | At2g46000 | 1097.70286 | 1435.249 | 1008.94487 | 2120.533 | 407.092806 | 865.308 | 0.570358262 | 0.588468 |
| 25092 | 39 | expressed protein contains Pfam profile PF04396: Protein of unknown function, DUF537 | At3g61100 | 2283.83891 | 3001.513 | 1880.64399 | 2961.003 | 1120.5439 | 2054.866 | 0.647115257 | 0.58608 |
| 18763 | 29 | expressed protein | At3g36800 | 1323.03794 | 2477.41 | 1315.94729 | 2188.039 | 641.603912 | 1699.99 | 0.504294922 | 0.585316 |
| 13375 | 21 | protease inhibitor/seed storage/lipid transfer protein (LTP) family protein contains Pfam protease inhibitor/seed storage/LTP family domain PF00234 | At3g53980 | 371.93155 | 1747.509 | 483.706159 | 3619.41 | 380.624093 | 1311.665 | 0.212220486 | 0.585144 |
| 14853 | 23 | eukaryotic translation initiation factor 3 subunit 9 / eIF-3 eta / eIF3b (TIF3B1) nearly identical to SP\|Q9C5Z1 Eukaryotic translation initiation factor 3 subunit 9 (eIF-3 eta) (eIF3 p110) (eIF3b) {Arabidopsis thaliana} | At5g27640 | 3927.36952 | 5253.568 | 3066.07271 | 4971.49 | 1649.12845 | 3906.036 | 0.595497828 | 0.58473 |
| 9218 | 15 | nicotianamine synthase, putative similar to nicotianamine synthase [Lycopersicon esculentum][GI:4753801], nicotianamine synthase 2 [Hordeum vulgare][GI:4894912] | At1g56430 | 494.173889 | 915.084 | 503.299719 | 854.625 | 323.79997 | 736.022 | 0.522958859 | 0.582309 |
| 11507 | 18 | GDSL-motif lipase/hydrolase family protein low similarity to family II lipases EXL3 GI:15054386, EXL1 GI:15054382 from [Arabidopsis thaliana]; contains Pfam profile PF00657: GDSL-like Lipase/Acylhydrolase | At4g30140 | 1067.23399 | 1726.25 | 374.31874 | 972.999 | 180.91387 | 1015.806 | 0.393681134 | 0.581856 |
| 5022 | 8 | peptidyl-prolyl cis-trans isomerase / cyclophilin (CYP1) / rotamase identical to cyclophilin (CYP1) gi\|992643\|gb\|AAA75512; similar to peptidyl-prolyl cis-trans isomerase, PPIase (cyclophilin, cyclosporin A-binding protein) [Catharanthus roseus] SWISS-PROT:Q39613 | At4g34870 | 2204.44894 | 3128.739 | 2313.53946 | 3141.263 | 2847.46608 | 5394.925 | 0.656294993 | 0.578676 |
| 9277 | 15 | plant defensin-fusion protein, putative (PDF2.1) plant defensin protein family member, personal communication, Bart Thomma (Bart.Thomma@agr.kuleuven.ac.be); contains a gamma-thionin family signature (PDOC00725) | At2g02120 | 908.955662 | 1859.646 | 213.800366 | 672.713 | 657.994019 | 2998.762 | 0.342006284 | 0.5742 |
| 30329 | 47 | drought-responsive protein / drought-induced protein (Di21) identical to GI:469112; contains PF03242 Late embryogenesis abundant protein domain | At4g15910 | 1253.79177 | 2738.148 | 1572.84329 | 2381.044 | 290.64449 | 636.892 | 0.524938218 | 0.5733 |
| 4681 | 8 | expressed protein | At1g67865 | 351.031063 | 635.47 | 186.729162 | 325.996 | 413.053782 | 1402.153 | 0.473259073 | 0.56848 |
| 10260 | 16 | amino acid permease 2 (AAP2) identical to amine acid permease AAP2 [Arabidopsis thaliana] GI:510236 | At5g09220 | 418.864325 | 589.08 | 396.139818 | 905.74 | 463.378771 | 897.368 | 0.554929877 | 0.5568 |
| 27815 | 43 | expressed protein contains Pfam profile PF04654: Protein of unknown function, DUF599 | At5g10580 | 539.729057 | 864.711 | 654.339188 | 1521.279 | 232.473941 | 544.776 | 0.493676743 | 0.55593 |
| 13352 | 21 | GDSL-motif lipase, putative similar to family II lipases EXL3 GI:15054386, EXL1 GI:15054382, EXL2 GI:15054384 from [Arabidopsis thaliana]; contains Pfam profile PF00657: GDSL-like Lipase/Acylhydrolase | At3g43570 | 808.171056 | 1092.098 | 854.790376 | 1499.823 | 241.880366 | 484.504 | 0.603059145 | 0.55593 |
| 19372 | 30 | expressed protein | At5g15860 | 502.501569 | 742.333 | 688.623537 | 1030.968 | 312.246976 | 510.158 | 0.652304121 | 0.555632 |
| 18717 | 29 | nuclear matrix protein-related low similarity to nuclear matrix protein p84 [Homo sapiens] GI:550058 | At5g09860 | 2369.9351 | 3190.201 | 2178.3364 | 3152.468 | 1110.03642 | 2371.137 | 0.634006246 | 0.5544 |
| 11885 | 19 | expressed protein | At2g07724 | 587.185922 | 728.83 | 661.682989 | 1217.21 | 277.50395 | 603.244 | 0.603093739 | 0.5544 |
| 29679 | 46 | NLI interacting factor (NIF) family protein contains Pfam profile PF03031: NLI interacting factor | At4g18140 | 951.397309 | 1356.064 | 817.067554 | 1317.567 | 670.417015 | 1455.117 | 0.594150523 | 0.552188 |
| 24556 | 38 | serine carboxypeptidase S10 family protein similar to Serine carboxypeptidase II chains A and B (SP:P08819) (EC 3.4.16.6) [Triticum aestivum (Wheat)]; carboxypeptidase D - Triticum aestivum, PIR:A29639 | At5g08260 | 359.578005 | 574.992 | 483.094308 | 568.647 | 235.632784 | 483.467 | 0.654097837 | 0.544896 |
| 11452 | 18 | kinesin-like protein A, putative kinesin like protein A, Arabidopsis thaliana, gb:Q07970 | At4g05190 | 357.99397 | 555.141 | 205.734772 | 271.04 | 349.831559 | 637.589 | 0.650868666 | 0.543348 |
| 18582 | 29 | protease inhibitor/seed storage/lipid transfer protein (LTP) family protein contains Pfam protease inhibitor/seed storage/LTP family domain PF00234 | At3g57310 | 1808.29053 | 2433.132 | 1164.49036 | 2419.208 | 873.539696 | 1603.555 | 0.589766141 | 0.53856 |
| 11196 | 18 | thaumatin-like protein, putative / pathogenesis-related protein, putative strong similarity to SP\|P50699 Thaumatin-like protein precursor {Arabidopsis thaliana}, pathogenesis-related group 5 protein [Brassica rapa] GI:2749943; contains Pfam profile: PF00314 thaumatin family | At1g73620 | 314.990799 | 766.03 | 241.085079 | 696.227 | 250.864099 | 762.172 | 0.362205464 | 0.537342 |
| 3652 | 6 | hypothetical protein | At4g07740 | 743.145418 | 909.828 | 698.061014 | 1530.925 | 515.225447 | 867.483 | 0.622234174 | 0.5248 |
| 20951 | 33 | protease inhibitor, putative (DR4) identical to Dr4 GI:469114 from [Arabidopsis thaliana]; contains Pfam profile PF00197: Trypsin and protease inhibitor | At1g73330 | 927.28597 | 1533.271 | 346.785461 | 567.672 | 585.418981 | 926.99 | 0.615731202 | 0.521985 |
| 13871 | 22 | osmotin-like protein, putative similar to SP\|Q41350 Osmotin-like protein precursor {Lycopersicon esculentum}; contains Pfam profile PF00314: Thaumatin family | At2g28790 | 543.733531 | 1105.388 | 473.787487 | 780.278 | 314.010031 | 725.105 | 0.510717277 | 0.521848 |
| 12897 | 20 | glycosyl hydrolase family 1 protein contains Pfam PF00232 : Glycosyl hydrolase family 1 domain; TIGRFAM TIGR01233: 6-phospho-beta-galactosidase; identical to thioglucosidase (GI:871992) [Arabidopsis thaliana]; similar to myrosinase precursor (EC 3.2.3.1)(Sinigrinase) (Thioglucosidase) SP\|P37702 from [Arabidops | At2g15390 | 598.91056 | 1080.771 | 593.624168 | 825.061 | 293.256927 | 705.079 | 0.563187708 | 0.52065 |
| 3108 | 5 | protease inhibitor/seed storage/lipid transfer protein (LTP) family protein contains Pfam protease inhibitor/seed storage/LTP family domain PF00234 | At5g05960 | 1051.94982 | 1265.282 | 145.49298 | 648.869 | 444.458703 | 1246.374 | 0.470740814 | 0.51901 |
| 4177 | 7 | hydroxyproline-rich glycoprotein family protein | At3g02120 | 492.188765 | 701.509 | 392.898922 | 576.932 | 390.15217 | 681.096 | 0.651819506 | 0.5148 |
| 29306 | 46 | cell division control protein, putative cdc2MsF [Medicago sativa] gi\|1806146\|emb\|CAA65982 | At1g20930 | 455.830838 | 713.948 | 378.692516 | 600.455 | 243.746392 | 482.075 | 0.591586735 | 0.511839 |
| 23745 | 37 | phosphoenolpyruvate carboxylase-related / PEP carboxylase-related identical to phosphoenolpyruvate carboxylase [Arabidopsis thaliana] GP:26800701 over first 45 residues | At3g42628 | 978.856405 | 1397.72 | 544.480134 | 895.105 | 352.786273 | 904.453 | 0.566221615 | 0.51128 |
| 23771 | 37 | protease inhibitor/seed storage/lipid transfer protein (LTP) family protein contains Pfam protease inhibitor/seed storage/LTP family domain PF00234 | At3g53980 | 289.973533 | 1284.963 | 305.750042 | 1803.251 | 263.229361 | 703.665 | 0.256435028 | 0.51051 |
| 20273 | 32 | expressed protein | At1g64370 | 883.111618 | 1173.42 | 1276.21205 | 1971.714 | 627.853048 | 1280.71 | 0.630031617 | 0.510048 |
| 9619 | 15 | inositol-3-phosphate synthase, putative / myo-inositol-1-phosphate synthase, putative / MI-1-P synthase, putative very strong similarity to SP\|Q38862 Myo-inositol-1-phosphate synthase isozyme 2 (EC 5.5.1.4) (MI-1-P synthase 2) (IPS 2) {Arabidopsis thaliana}; identical to SP\|Q9LX12\| Probable inositol-3-phosphate synt | At5g10170 | 291.392854 | 425.977 | 282.275055 | 396.159 | 272.821357 | 507.596 | 0.644688259 | 0.503644 |
| 14054 | 22 | hypothetical protein weak similarity to SP\|Q13200 26S proteasome non-ATPase regulatory subunit 2 (26S proteasome regulatory subunit S2) (26S proteasome subunit p97) (Tumor necrosis factor type 1 receptor associated protein 2) {Homo sapiens} | At4g08140 | 361.339588 | 553.448 | 505.884151 | 750.272 | 247.552674 | 549.457 | 0.592565433 | 0.50184 |
| 8667 | 14 | expressed protein contains similarity to anther-specific protein GI:1448935 from [Brassica rapa] | At2g28355 | 375.209785 | 507.529 | 286.354059 | 477.929 | 351.926457 | 573.296 | 0.657695545 | 0.495936 |
| 23595 | 37 | xyloglucan fucosyltransferase, putative (FUT4) identical to SP\|Q9SJP2 Probable fucosyltransferase 4 (EC 2.4.1.-) (AtFUT4) {Arabidopsis thaliana}; similar to SP\|Q9SWH5 Galactoside 2-alpha-L-fucosyltransferase (EC 2.4.1.69) (Xyloglucan alpha-(1,2)-fucosyltransferase) (AtFUT1) {Arabidopsis thaliana} | At2g15390 | 586.769859 | 861.373 | 640.0324 | 932.297 | 343.761302 | 817.683 | 0.59604109 | 0.48708 |
| 21418 | 33 | YT521-B-like family protein contains Pfam profile PF04146: YT521-B-like family | At5g61020 | 470.345986 | 920.834 | 560.561927 | 870.642 | 233.106947 | 417.703 | 0.570900061 | 0.484352 |
| 22637 | 35 | chaperonin, putative similar to SWISS-PROT:P78371- T-complex protein 1, beta subunit (TCP-1-beta) [Homo sapiens]; contains Pfam profile: PF00118 domain, TCP-1/cpn60 chaperonin family | At5g20890 | 433.034002 | 647.133 | 484.149113 | 734.535 | 209.906358 | 513.743 | 0.57895445 | 0.48074 |
| 6762 | 11 | glycosyl hydrolase family protein 17 similar to glucan endo-1,3-beta-glucosidase precursor SP:P52409 from [Triticum aestivum]; C terminal homology only | At2g43660 | 616.220497 | 847.724 | 614.6884 | 910.958 | 363.55768 | 888.53 | 0.603616897 | 0.472857 |
| 13442 | 21 | pectate lyase family protein similar to pectate lyase 2 GP:6606534 from [Musa acuminata] | At4g22090 | 572.25899 | 763.364 | 483.608964 | 669.344 | 173.268315 | 446.826 | 0.6199806 | 0.4698 |
| 19305 | 30 | beta-expansin, putative (EXPB3) similar to soybean pollen allergen (cim1) protein - soybean, PIR2:S48032; beta-expansin gene family, PMID:11641069 | At4g28250 | 624.878674 | 788.971 | 642.208931 | 911.365 | 177.010678 | 455.978 | 0.62829481 | 0.463884 |
| 27422 | 43 | flavin-containing monooxygenase family protein / FMO family protein similar to flavin-containing monooxygenase GB:AAA21178 GI:349534 SP\|P32417 from [Oryctolagus cuniculus]; contains Pfam profile PF00743 Flavin-binding monooxygenase-like | At1g62560 | 511.252799 | 862.439 | 506.408366 | 1058.972 | 201.718522 | 405.229 | 0.522931755 | 0.446958 |
| 21199 | 33 | meprin and TRAF homology domain-containing protein / MATH domain-containing protein weak similarity to ubiquitin-specific protease 12 [Arabidopsis thaliana] GI:11993471; contains Pfam profile PF00917: MATH domain | At4g01390 | 516.512949 | 878.815 | 532.496466 | 906.883 | 216.556012 | 471.764 | 0.554624826 | 0.4437 |
| 26713 | 42 | ubiquitin extension protein, putative / 40S ribosomal protein S27A (RPS27aA) strong similarity to ubiquitin extension protein (UBQ5) GB:AAA32906 GI:166934 from (Arabidopsis thaliana) | At1g23410 | 377.971417 | 590.929 | 412.843022 | 686.467 | 155.824573 | 376.35 | 0.551688869 | 0.442953 |
| 18575 | 29 | protease inhibitor/seed storage/lipid transfer protein (LTP) family protein contains Pfam protease inhibitor/seed storage/LTP family domain PF00234 | At3g53980 | 331.807663 | 1741.057 | 301.812847 | 2860.952 | 214.056979 | 728.039 | 0.196696901 | 0.431172 |
| 20098 | 31 | expressed protein ; expression supported by MPSS | At5g50730 | 456.323911 | 656.154 | 454.201893 | 715.897 | 194.404934 | 353.037 | 0.626856128 | 0.430582 |
| 15183 | 24 | glycine cleavage system H protein, mitochondrial, putative similar to SP\|Q39732 Glycine cleavage system H protein, mitochondrial precursor {Flaveria anomala}; contains Pfam profile PF01597: Glycine cleavage H-protein | At2g35120 | 253.838077 | 406.273 | 833.162093 | 1133.874 | 311.855337 | 601.308 | 0.626072529 | 0.42994 |
| 27149 | 42 | phenylalanine ammonia-lyase 3 (PAL3) nearly identical to SP\|P45725 | At5g04230 | 339.548149 | 436.663 | 343.687968 | 538.039 | 151.993548 | 375.996 | 0.605691259 | 0.4284 |
| 29188 | 45 | senescence-associated protein-related similar to senescence-associated protein SAG102 (GI:22331931) [Arabidopsis thaliana]; | At5g47060 | 165.309685 | 315.566 | 215.850127 | 434.021 | 187.198565 | 229.833 | 0.611892701 | 0.42484 |
| 28135 | 44 | Ulp1 protease family protein contains Pfam profile PF02902: Ulp1 protease family, C-terminal catalytic domain; similar to At5g28270, At2g05450, At1g45090, At2g16180, At2g06750 | At2g12100 | 737.587927 | 997.279 | 230.927403 | 353.465 | 313.366716 | 679.171 | 0.618106982 | 0.4059 |
| 22190 | 35 | nodulin MtN21 family protein similar to MtN21 [Medicago truncatula] GI:2598575; contains Pfam profile PF00892: Integral membrane protein | At1g44800 | 284.702687 | 420.238 | 331.489196 | 1005.901 | 219.166387 | 451.208 | 0.497585561 | 0.4056 |
| 4159 | 7 | protein kinase family protein contains protein kinase domain, Pfam:PF00069 | At2g42550 | 410.620072 | 476.178 | 366.183038 | 621.753 | 181.775747 | 395.919 | 0.636800303 | 0.405232 |
| 14754 | 23 | xyloglucan:xyloglucosyl transferase / xyloglucan endotransglycosylase / endo-xyloglucan transferase (XTR9) identical to xyloglucan endotransglycosylase (XTR9) identical to xyloglucan endotransglycosylase GI:4218963 from [Arabidopsis thaliana] | At4g25820 | 194.046919 | 257.087 | 276.129461 | 551.261 | 259.693189 | 382.806 | 0.644696572 | 0.403172 |
| 24068 | 38 | glutaredoxin family protein contains INTERPRO Domain IPR002109, Glutaredoxin (thioltransferase) | At1g06830 | 367.734553 | 701.687 | 409.492184 | 556.394 | 154.348247 | 443.537 | 0.536013777 | 0.394912 |
| 12773 | 20 | expressed protein | At4g14590 | 1053.00335 | 1519.169 | 1052.31934 | 1422.566 | 482.719468 | 1102.321 | 0.623596474 | 0.394632 |
| 27847 | 43 | plastocyanin-like domain-containing protein | At5g25090 | 620.9234 | 1146.757 | 447.939985 | 854.859 | 218.739572 | 544.092 | 0.489159983 | 0.393624 |
| 20620 | 32 | expressed protein | At4g34600 | 317.756709 | 415.997 | 384.465262 | 785.524 | 171.245583 | 297.323 | 0.609746595 | 0.392445 |
| 24349 | 38 | CCAAT-binding transcription factor (CBF-B/NF-YA) family protein contains Pfam profile: PF02045 CCAAT-binding transcription factor (CBF-B/NF-YA) subunit B | At3g14020 | 451.556833 | 633.931 | 712.821911 | 986.723 | 185.703682 | 455.764 | 0.614060487 | 0.39216 |
| 21594 | 34 | endo-1,4-beta-glucanase (EGASE) / cellulase identical to endo-1,4-beta-glucanase GB:CAA67157 GI:2440035 from [Arabidopsis thaliana] | At1g70710 | 355.95109 | 513.735 | 304.462607 | 388.042 | 128.838321 | 369.688 | 0.608662376 | 0.39204 |
| 9335 | 15 | meprin and TRAF homology domain-containing protein / MATH domain-containing protein low similarity to ubiquitin-specific protease 12 [Arabidopsis thaliana] GI:11993471; contains Pfam profile PF00917: MATH domain | At2g32880 | 264.130558 | 373.146 | 312.391171 | 380.781 | 157.938029 | 423.64 | 0.633685181 | 0.390432 |
| 10943 | 17 | expressed protein | At5g22090 | 458.068381 | 737.156 | 421.219321 | 677.635 | 151.251261 | 285.908 | 0.590674109 | 0.38181 |
| 23954 | 37 | WD-40 repeat family protein contains 5 WD-40 repeats (PF00400); similar to fizzy1 (GI:3298595) {Xenopus laevis}; similar to "Will die slowly" protein, Drosophila; putative cdc20 protein - Arabidopsis thaliana, EMBL:AF029262 | At5g27570 | 402.367262 | 805.121 | 417.256632 | 600.076 | 115.151376 | 322.772 | 0.517285757 | 0.376488 |
| 15829 | 25 | glutaredoxin family protein contains INTERPRO Domain IPR002109, Glutaredoxin (thioltransferase) | At2g30540 | 456.15064 | 791.288 | 442.809362 | 658.191 | 180.847889 | 490.621 | 0.539281151 | 0.374 |
| 1809 | 3 | aspartyl protease family protein contains Pfam profile:PF00026 eukaryotic aspartyl protease | At5g07030 | 428.354629 | 641.316 | 275.820349 | 647.199 | 186.784122 | 616.604 | 0.465676706 | 0.368064 |
| 24351 | 38 | expressed protein | At3g14190 | 372.774372 | 631.416 | 209.81537 | 326.527 | 144.758313 | 344.681 | 0.550974242 | 0.36421 |
| 11515 | 18 | F-box family protein contains Pfam PF00646: F-box domain; contains TIGRFAM TIGR01640 : F-box protein interaction domain | At4g33290 | 460.425715 | 628.197 | 91.4987554 | 142.514 | 208.652718 | 427.651 | 0.620956567 | 0.363312 |
| 20091 | 31 | pectinesterase family protein contains Pfam profile: PF01095 pectinesterase | At5g47500 | 460.051366 | 957.361 | 308.802922 | 581.653 | 139.032395 | 522.187 | 0.425899037 | 0.3608 |
| 14535 | 23 | meprin and TRAF homology domain-containing protein / MATH domain-containing protein low similarity to ubiquitin-specific protease 12 [Arabidopsis thaliana] GI:11993471; contains Pfam profile PF00917: MATH domain | At2g32870 | 312.039852 | 393.195 | 201.698789 | 317.423 | 186.949075 | 555.56 | 0.588510762 | 0.355656 |
| 30922 | 48 | speckle-type POZ protein-related contains Pfam profile PF00651 : BTB/POZ domain; similar to Speckle-type POZ protein (SP:O43791) [Homo sapiens] | At3g56230 | 519.755888 | 616.353 | 659.637114 | 1187.418 | 130.184747 | 294.94 | 0.613397522 | 0.352976 |
| 15294 | 24 | hypothetical protein | At3g42600 | 408.265946 | 517.148 | 414.261113 | 633.582 | 213.12912 | 480.016 | 0.629100221 | 0.3285 |
| 22852 | 36 | 4-coumarate--CoA ligase 1 / 4-coumaroyl-CoA synthase 1 (4CL1) identical to SP\|Q42524 4-coumarate--CoA ligase 1 (EC 6.2.1.12) (4CL 1) (4-coumaroyl-CoA synthase 1) {Arabidopsis thaliana} | At1g51680 | 387.672426 | 482.786 | 438.800466 | 598.281 | 120.466975 | 327.053 | 0.634922145 | 0.317856 |
| 30629 | 48 | proton-dependent oligopeptide transport (POT) family protein contains Pfam profile: PF00854 POT family | At1g32450 | 536.550292 | 596.608 | 120.662042 | 284.602 | 187.730538 | 386.8 | 0.602881677 | 0.313728 |
| 24496 | 38 | argininosuccinate synthase family protein contains Pfam profile: PF00764 arginosuccinate synthase | At4g24830 | 423.768457 | 629.11 | 648.056566 | 755.203 | 107.08313 | 287.829 | 0.634628391 | 0.310068 |
| 21825 | 34 | expressed protein contains Pfam profile PF03759: Domain of unknown function (DUF315) | At3g55660 | 295.811251 | 488.086 | 324.293578 | 446.502 | 141.302555 | 334.728 | 0.584834456 | 0.30217 |
| 24602 | 38 | expressed protein | At5g27330 | 265.760585 | 446.226 | 223.680797 | 326.146 | 109.866706 | 199.156 | 0.611021921 | 0.29546 |
| 29665 | 46 | bile acid:sodium symporter family protein low similarity to SP\|Q12908 Ileal sodium/bile acid cotransporter {Homo sapiens}; contains Pfam profile PF01758: Sodium Bile acid symporter family | At4g12030 | 258.413701 | 322.805 | 236.120167 | 345.043 | 105.107822 | 310.879 | 0.607648505 | 0.284746 |
| 27420 | 43 | mitogen-activated protein kinase, putative / MAPK, putative (MPK2) identical to mitogen-activated protein kinase homolog 2 (AtMPK2)[Arabidopsis thaliana] SWISS-PROT:Q39022; PMID:12119167 | At1g59580 | 479.800994 | 640.009 | 318.445943 | 575.815 | 132.08995 | 360.668 | 0.555316877 | 0.284 |
| 27891 | 43 | formin homology 2 domain-containing protein / FH2 domain-containing protein contains formin homology 2 domain, Pfam:PF02181 | At5g48360 | 314.446387 | 440.734 | 361.806076 | 499.494 | 110.291459 | 264.13 | 0.618456962 | 0.28208 |
| 25035 | 39 | aminoalcoholphosphotransferase, putative strong similarity to aminoalcoholphosphotransferase [Arabidopsis thaliana] GI:3661593; contains Pfam profile PF01066: CDP-alcohol phosphatidyltransferase | At3g25585 | 239.348598 | 333.756 | 191.802424 | 302.924 | 132.236346 | 255.278 | 0.622771918 | 0.2772 |
| 14395 | 23 | cyclin, putative similar to mitotic cyclin a2-type [Glycine max] GI:857397, cyclin A-like protein [Nicotiana tabacum] GI:1064927; contains Pfam profiles PF00134: Cyclin, N-terminal domain, PF02984: Cyclin, C-terminal domain | At1g44110 | 189.695904 | 322.342 | 249.141113 | 346.016 | 202.048427 | 336.446 | 0.636352618 | 0.27531 |
| 12396 | 20 | cytochrome P450, putative similar to gb\|AF069494 cytochrome P450 from Sinapis alba and is a member of the PF\|00067 Cytochrome P450 family | At1g16410 | 220.686724 | 315.717 | 423.596615 | 554.702 | 113.002868 | 245.587 | 0.640927547 | 0.27255 |
| 22291 | 35 | expressed protein | At2g11090 | 307.080252 | 380.839 | 311.74108 | 501.57 | 113.877117 | 206.711 | 0.659585442 | 0.26832 |
| 6998 | 11 | legume lectin family protein contains Pfam domain, PF00139: Legume lectins beta domain | At5g03350 | 161.415377 | 246.834 | 236.403785 | 296.647 | 108.462547 | 210.752 | 0.65516932 | 0.264187 |
| 7510 | 12 | peroxidase 33 (PER33) (P33) (PRXCA) / neutral peroxidase C (PERC) identical to SP\|P24101 Peroxidase 33 precursor (EC 1.11.1.7) (Atperox P33) (ATPCa) (Neutral peroxidase C) (PERC) {Arabidopsis thaliana} | At3g49110 | 147.87886 | 236.745 | 158.676449 | 179.74 | 185.068614 | 409.872 | 0.652990815 | 0.261702 |
| 23324 | 36 | expressed protein | At5g42530 | 376.845159 | 787.226 | 467.751835 | 1851.247 | 179.029286 | 330.002 | 0.424626103 | 0.25542 |
| 22615 | 35 | expressed protein contains Pfam profile PF04654: Protein of unknown function, DUF599 | At5g10580 | 233.191078 | 511.797 | 284.582241 | 632.985 | 96.5426564 | 292.272 | 0.411845824 | 0.25256 |
| 10800 | 17 | nodulin MtN21 family protein similar to MtN21 GI:2598575 (root nodule development) from [Medicago truncatula] | At4g01450 | 243.821544 | 311.583 | 95.0184899 | 128.549 | 143.226315 | 316.98 | 0.657844454 | 0.2508 |
| 4647 | 8 | jacalin lectin family protein similar to myrosinase-binding protein homolog [Arabidopsis thaliana] GI:2997767; contains Pfam profile PF01419 jacalin-like lectin domain | At1g52060 | 274.355657 | 344.012 | 70.165245 | 221.156 | 180.206635 | 909.797 | 0.437618998 | 0.250245 |
| 30462 | 47 | expressed protein | At5g36710 | 321.008205 | 461.86 | 237.815503 | 344.877 | 101.437626 | 296.149 | 0.575707326 | 0.24871 |
| 28217 | 44 | fumarate hydratase, putative / fumarase, putative similar to SP\|P55250 Fumarate hydratase, mitochondrial precursor (EC 4.2.1.2) (Fumarase) {Rhizopus oryzae}; contains Pfam profile PF00206: Lyase | At2g47510 | 198.370125 | 300.638 | 249.453411 | 308.621 | 79.4164485 | 290.447 | 0.580514286 | 0.241345 |
| 20108 | 31 | late embryogenesis abundant protein-related / LEA protein-related similar to late embryogenesis abundant protein (GI:1350543)[Picea glauca] | At5g54370 | 308.855739 | 344.411 | 233.602656 | 410.687 | 111.627575 | 451.884 | 0.570867186 | 0.236652 |
| 8829 | 14 | expressed protein | At3g59680 | 196.977543 | 305.844 | 182.529063 | 316.071 | 176.047767 | 261.65 | 0.631458844 | 0.234423 |
| 10028 | 16 | expressed protein | At3g04630 | 142.214438 | 197.967 | 168.71781 | 209.428 | 92.1260494 | 247.957 | 0.631842457 | 0.233472 |
| 15745 | 25 | calmodulin-binding family protein low similarity to SF16 protein [Helianthus annuus] GI:560150; contains Pfam profile PF00612: IQ calmodulin-binding motif | At1g72670 | 245.650511 | 364.61 | 201.897959 | 266.825 | 106.159395 | 241.175 | 0.62352628 | 0.22752 |
| 27631 | 43 | homocysteine S-methyltransferase 1 (HMT-1) identical to GB:AAF23821 from [Arabidopsis thaliana] | At3g25900 | 200.819442 | 235.057 | 229.537992 | 362.796 | 77.2287641 | 174.171 | 0.643480978 | 0.21912 |
| 26953 | 42 | disease resistance-responsive protein-related / dirigent protein-related similar to pathogenesis-related protein [Pisum sativum] gi\|4585273\|gb\|AAD25355; similar to dirigent protein [Forsythia x intermedia] gi\|6694695\|gb\|AAF25358 | At3g13662 | 245.638745 | 328.708 | 758.197645 | 910.458 | 68.7316413 | 176.043 | 0.656825323 | 0.217294 |
| 27199 | 42 | hypothetical protein | At5g26050 | 255.256328 | 373.475 | 294.247569 | 398.233 | 67.3749058 | 203.288 | 0.584590594 | 0.217056 |
| 26988 | 42 | hypothetical protein similar to hypothetical protein GB:CAB55689 GI:5881771 from [Arabidopsis thaliana] | At3g30160 | 136.52539 | 225.095 | 194.189279 | 282.637 | 87.3898468 | 172.838 | 0.597734332 | 0.213774 |
| 455 | 1 | expressed protein weak similarity to phragmoplast-associated kinesin-related protein 1 [Arabidopsis thaliana] GI:8745333 | At4g26660 | 139.177926 | 185.446 | 114.05756 | 167.135 | 132.271398 | 249.486 | 0.654369056 | 0.21216 |
| 13064 | 21 | nodulin MtN21 family protein similar to MtN21 GI:2598575 (root nodule development) from [Medicago truncatula] | At1g21890 | 252.5332 | 564.757 | 166.662374 | 336.488 | 76.4967868 | 309.715 | 0.396481413 | 0.20655 |
| 17634 | 28 | expressed protein | At1g35320 | 177.451455 | 267.99 | 323.907984 | 463.97 | 92.427088 | 164.793 | 0.640382499 | 0.195734 |
| 13021 | 21 | male sterility MS5 family protein similar to male sterility MS5 [Arabidopsis thaliana] GI:3859112; contains Pfam profile PF00515 TPR Domain | At1g04770 | 187.823085 | 278.071 | 123.557179 | 194.173 | 105.303703 | 173.799 | 0.63922298 | 0.195734 |
| 22084 | 34 | miRNA gene Arabidopsis thaliana miR397a stem-loop | ath-MIR39 | 196.198896 | 286.5 | 267.01703 | 389.183 | 79.5133582 | 138.672 | 0.648100274 | 0.186124 |
| 28054 | 44 | leucine-rich repeat protein kinase, putative similar to light repressible receptor protein kinase [Arabidopsis thaliana] gi\|1321686\|emb\|CAA66376; contains leucine rich repeat (LRR) domains, Pfam:PF00560; contains protein kinase domain, Pfam:PF00069 | At1g51860 | 171.167725 | 232.564 | 214.923685 | 357.197 | 97.2725718 | 167.722 | 0.639220293 | 0.173052 |
| 20140 | 31 | peroxidase 73 (PER73) (P73) (PRXR11) identical to SP\|Q43873 Peroxidase 73 precursor (EC 1.11.1.7) (Atperox P73) (PRXR11) (ATP10a) {Arabidopsis thaliana} | At5g67400 | 138.3458 | 330.899 | 162.78732 | 450.893 | 159.53807 | 350.846 | 0.411282597 | 0.171288 |
| 27851 | 43 | expressed protein | At5g25475 | 251.763109 | 320.981 | 235.495569 | 332.573 | 87.3197419 | 182.778 | 0.656731224 | 0.167552 |
| 9386 | 15 | formin homology 2 domain-containing protein / FH2 domain-containing protein contains formin homology 2 domain, Pfam:PF02181 | At3g05470 | 139.229266 | 231.095 | 593.941246 | 1137.604 | 94.4127054 | 180.77 | 0.548951856 | 0.16384 |
| 28914 | 45 | leucine-rich repeat transmembrane protein kinase, putative similar to receptor kinase GB:AAA33715 from [Petunia integrifolia] | At3g17840 | 121.469039 | 186.045 | 159.124183 | 199.559 | 79.2370625 | 194.169 | 0.6194545 | 0.15799 |
| 13591 | 21 | major intrinsic family protein / MIP family protein contains Pfam profile: MIP PF00230 | At5g47450 | 135.611977 | 298.31 | 171.21142 | 203.654 | 97.5035055 | 249.591 | 0.561983841 | 0.15732 |
| 12053 | 19 | expressed protein | At3g47510 | 112.777705 | 184.261 | 237.718308 | 325.57 | 99.039627 | 220.13 | 0.597376249 | 0.156078 |
| 8017 | 13 | serine/threonine protein kinase, putative similar to serine/threonine kinase Ayk1 [Mus musculus] gi\|1763647\|gb\|AAB62982 | At2g25880 | 197.83427 | 338.81 | 152.094274 | 224.074 | 107.429365 | 314.551 | 0.53473673 | 0.150282 |
| 26671 | 42 | fatty acid desaturase family protein similar to delta 9 acyl-lipid desaturase (ADS1) GI:2970034 from [Arabidopsis thaliana] | At1g06360 | 217.992474 | 316.877 | 117.561361 | 220.91 | 47.6878082 | 204.071 | 0.484597122 | 0.147436 |
| 30127 | 47 | proliferating cell nuclear antigen 2 (PCNA2) identical to SP\|Q9ZW35 Proliferating cell nuclear antigen 2 (PCNA 2) {Arabidopsis thaliana}; nearly identical to SP\|Q43124 Proliferating cell nuclear antigen (PCNA) {Brassica napus}; contains Pfam profiles PF00705: Proliferating cell nuclear antigen N-terminal domain, PF027- | At2g29570 | 169.43609 | 302.153 | 172.639071 | 200.193 | 93.5673231 | 275.378 | 0.587634502 | 0.146615 |
| 21547 | 34 | heavy-metal-associated domain-containing protein contains Pfam profile PF00403: Heavy-metal-associated domain | At1g51090 | 193.992371 | 291.616 | 157.884548 | 445.434 | 69.3089755 | 129.883 | 0.517769846 | 0.14592 |
| 24807 | 39 | legume lectin family protein | At1g53060 | 311.619511 | 526.524 | 296.822441 | 525.666 | 66.0820895 | 149.763 | 0.532582378 | 0.14276 |
| 14781 | 23 | Rac-like GTP-binding protein (ARAC3) / Rho-like GTP-binding protein (ROP6) identical to SP\|Q38912 RAC-like GTP binding protein ARAC3 (GTP-binding protein ROP6) {Arabidopsis thaliana}; identical to cDNA Rho-like GTP binding protein (Rop6) GI:2645642 | At4g35020 | 216.973173 | 250.697 | 267.925246 | 348.511 | 56.4859697 | 164.736 | 0.65904628 | 0.138138 |

| | | | | | | | | | | | |
|---|---|---|---|---|---|---|---|---|---|---|---|
| 27667 | 43 | leucine-rich repeat transmembrane protein kinase, putative brassinosteroid-insensitive protein BRI1 - Arabidopsis thaliana, PIR:T09356 | At3g51740 | 196.247026 | 326.862 | 188.418125 | 279.272 | 61.2324819 | 160.268 | 0.552378759 | 0.1343 |
| 24559 | 38 | calcium-binding EF hand family protein contains INTERPRO:IPR002048 calcium-binding EF-hand domain | At5g08580 | 167.285183 | 254.159 | 195.361992 | 228.728 | 53.6879605 | 173.942 | 0.606989696 | 0.126 |
| 23410 | 37 | nodulin MtN21 family protein similar to MtN21 GI:2598575 (root nodule development) from [Medicago truncatula] | At1g01070 | 273.99949 | 359.85 | 160.021245 | 231.997 | 37.7555974 | 129.495 | 0.580914298 | 0.125925 |
| 16995 | 27 | cyclin, putative similar to cyclin A-like protein [Nicotiana tabacum] GI:1064931, A-type cyclin [Catharanthus roseus] GI:2190259; contains Pfam profiles PF00134: Cyclin, N-terminal domain, PF02984: Cyclin, C-terminal domain | At1g47230 | 330.716701 | 465.848 | 244.434324 | 283.571 | 58.1190008 | 158.491 | 0.646204198 | 0.123662 |
| 2349 | 4 | pollen Ole e 1 allergen and extensin family protein contains Pfam domain, PF01190: Pollen proteins Ole e I family | At4g02270 | 86.9037133 | 134.846 | 175.518274 | 204.913 | 150.506913 | 350.04 | 0.643662402 | 0.12078 |
| 16125 | 25 | expressed protein contains Pfam profile PF03267: Arabidopsis protein of unknown function, DUF266 | At5g14550 | 221.634363 | 306.758 | 256.790839 | 374.008 | 54.1230231 | 119.538 | 0.620621918 | 0.11952 |
| 24954 | 39 | expansin, putative (EXP4) similar to alpha-expansin 6 precursor GI:16923359 from [Cucumis sativus]; alpha-expansin gene family, PMID:11641069 | At2g39700 | 313.122258 | 451.845 | 262.670341 | 431.73 | 41.0154739 | 185.05 | 0.507681604 | 0.118524 |
| 17227 | 27 | CXC domain protein (TSO1) identical to CXC domain protein TSO1 [Arabidopsis thaliana] GI:7767425 | At3g22780 | 190.786866 | 331.456 | 301.647138 | 694.989 | 128.908426 | 159.709 | 0.605593193 | 0.117612 |
| 14772 | 23 | expressed protein contains Pfam profile PF04862: Protein of unknown function, DUF642 | At4g32460 | 182.661335 | 238.157 | 241.045245 | 315.074 | 69.6368189 | 196.411 | 0.62885612 | 0.11592 |
| 22308 | 35 | reticulon family protein non-consensus AA donor splice site at 46485 and non-consensus GG acceptor splice site at 46563, confirmed by Ceres cDNA 1885; contains Pfam profile PF02453: Reticulon | At2g20590 | 185.653995 | 271.146 | 168.091619 | 260.289 | 41.1185693 | 153.383 | 0.53285579 | 0.115425 |
| 24366 | 38 | hypothetical protein | At3g20555 | 131.228875 | 375.267 | 327.811719 | 364.327 | 42.8567578 | 196.792 | 0.489081649 | 0.1136 |
| 29342 | 46 | glutamine synthetase, putative similar to glutamine synthetase, cytosolic isozyme (glutamate-- ammonia ligase, GS1) [Arabidopsis thaliana] SWISS-PROT:Q9LVI8 | At1g48470 | 177.774465 | 261.949 | 168.458092 | 251.445 | 54.90861 | 128.389 | 0.592098129 | 0.11297 |
| 24548 | 38 | nicotianamine synthase, putative similar to nicotianamine synthase [Lycopersicon esculentum][GI:4753801], nicotianamine synthase 2 [Hordeum vulgare][GI:4894912] | At5g04950 | 530.108266 | 678.564 | 620.203022 | 990.784 | 29.8873565 | 106.054 | 0.563001781 | 0.11008 |
| 19467 | 30 | proton-dependent oligopeptide transport (POT) family protein contains Pfam profile: PF00854 POT family | At5g62680 | 266.409814 | 350.197 | 289.445179 | 382.729 | 44.9207277 | 158.249 | 0.600290137 | 0.105825 |
| 16950 | 27 | sulfotransferase family protein similar to SP\|P52837 Flavonol 4'-sulfotransferase (EC 2.8.2.-) (F4-ST) {Flaveria chloraefolia}; contains Pfam profile PF00685: Sulfotransferase domain | At1g18590 | 205.770486 | 308.487 | 234.354723 | 395.637 | 73.0204099 | 108.9 | 0.643302109 | 0.105 |
| 5860 | 10 | endo-1,4-beta-glucanase / cellulase (CEL2) identical to endo-1,4-beta glucanase; ATCEL2 GI:3132891 from [Arabidopsis thaliana] | At1g02800 | 99.8230614 | 158.293 | 110.276515 | 169.345 | 75.507071 | 162.808 | 0.581865445 | 0.104784 |
| 24272 | 38 | Dof-type zinc finger domain-containing protein similar to zinc finger protein OBP2 GI:5059394 from [Arabidopsis thaliana] | At2g28810 | 239.056605 | 307.544 | 392.138889 | 434.701 | 32.2028791 | 110.707 | 0.656760418 | 0.104576 |
| 11671 | 18 | AT hook motif-containing protein contains Pfam PF03479: Domain of unknown function (DUF296); contains Pfam PF02178: AT hook motif; similar to AT-Hook DNA-Binding Protein SAP1 protein (GI:4165183) [Antirrhinum majus]; similar to AT-hook protein 2, Arabidopsis thaliana, EMBL:ATAJ4119 | At5g62260 | 98.6262115 | 146.604 | 150.958208 | 209.545 | 84.7959665 | 144.892 | 0.65946135 | 0.1036 |
| 27876 | 43 | expressed protein | At5g41860 | 162.073163 | 197.187 | 214.208266 | 296.248 | 52.1951391 | 129.812 | 0.649026503 | 0.102638 |
| 27893 | 43 | superoxide dismutase [Fe], putative / iron superoxide dismutase, putative similar to Fe-superoxide dismutase precursor [Medicago sativa] gi\|16974682\|gb\|AAL32441 | At5g51100 | 177.776605 | 224.556 | 135.526507 | 194.556 | 76.0782195 | 195.981 | 0.625488707 | 0.100768 |
| 24543 | 38 | leucine-rich repeat transmembrane protein kinase, putative leucine-rich receptor-like protein (LRPKm1) - Malus domestica, EMBL:AF053127 | At5g01890 | 184.979096 | 287.745 | 318.07469 | 373.146 | 35.9885422 | 130.682 | 0.590220476 | 0.099484 |
| 18194 | 28 | expressed protein | At1g35320 | 179.219456 | 222.982 | 109.78098 | 236.44 | 86.6599313 | 191.097 | 0.573844721 | 0.0966 |
| 10102 | 16 | oxidoreductase, 2OG-Fe(II) oxygenase family protein similar to hyoscyamine 6 beta-hydroxylase from Atropa belladonna [GI:4996123] and Hyoscyamus niger [SP\|P24397], gibberellin 20-oxidase [GI:9791186]; contains PF03171 2OG-Fe(II) oxygenase superfamily domain | At3g46490 | 128.29825 | 142.66 | 74.4306724 | 103.679 | 77.9648653 | 224.593 | 0.654787542 | 0.09656 |
| 3880 | 6 | GDP-D-mannose 4,6-dehydratase, putative strong similarity to GDP-D-mannose-4,6-dehydratase [Arabidopsis thaliana] GI:1764100 | At5g66280 | 107.646973 | 124.61 | 86.9082824 | 162.41 | 60.3231804 | 151.515 | 0.599040342 | 0.09646 |
| 12676 | 20 | cyclopropane-fatty-acyl-phospholipid synthase family protein similar to cyclopropane synthase [Sterculia foetida] GI:21069167; contains Pfam profile PF02353: Cyclopropane-fatty-acyl-phospholipid synthase | At3g23470 | 113.760641 | 132.701 | 137.895834 | 207.633 | 69.8223906 | 203.003 | 0.621783516 | 0.09504 |
| 17443 | 27 | hypothetical protein | At5g22180 | 277.774006 | 379.547 | 276.500714 | 611.699 | 39.4071857 | 273.349 | 0.442680638 | 0.09296 |
| 30173 | 47 | expressed protein | At3g01680 | 206.944875 | 266.045 | 121.892117 | 182.325 | 131.481687 | 304.764 | 0.625940353 | 0.092664 |
| 25236 | 39 | heavy-metal-associated domain-containing protein / copper chaperone (CCH)-related similar to copper homeostasis factor [Arabidopsis thaliana][GI:3168840], and farnesylated proteins GMFP7 [Glycine max][GI:4097573], ATFP7 [GI:4097555], and ATFP6 [GI:4097553]; contains heavy-metal-associated domain PF0040 | At5g17450 | 169.217897 | 205.628 | 246.045212 | 300.145 | 51.1848042 | 162.41 | 0.652614876 | 0.092416 |
| 23884 | 37 | O-methyltransferase family 2 protein similar to caffeic acid O-methyltransferase, Pinus taeda, gb:U39301 | At4g35150 | 157.96708 | 196.505 | 210.774573 | 249.083 | 35.0895503 | 116.58 | 0.650358864 | 0.091542 |
| 14569 | 23 | WRKY family transcription factor contains Pfam profile: PF03106 WRKY DNA -binding domain | At2g46130 | 121.958903 | 170.274 | 126.068316 | 184.102 | 86.8166363 | 155.581 | 0.653013614 | 0.0912 |
| 27654 | 43 | chitinase, putative similar to basic endochitinase CHB4 precursor SP:Q06209 from [Brassica napus] | At3g47540 | 156.37235 | 178.654 | 193.703303 | 248.895 | 56.4735982 | 175.838 | 0.658233953 | 0.090919 |
| 21173 | 33 | DJ-1 family protein low similarity to SP\|Q51732 protease I from Pyrococcus furiosus; contains Pfam profile: PF01965 DJ-1/PfpI family | At3g54600 | 307.969065 | 415.519 | 316.823901 | 441.287 | 55.7374971 | 165.593 | 0.598571616 | 0.085272 |
| 19071 | 30 | expressed protein | At2g28780 | 126.874651 | 212.868 | 256.121627 | 377.385 | 33.8029198 | 156.604 | 0.496849767 | 0.084864 |
| 27627 | 43 | CXC domain containing TSO1-like protein 1 (SOL1) identical to CXC domain containing TSO1-like protein 1 (SOL1) [Arabidopsis thaliana] GI:7767427; contains Pfam profile PF03638: Tesmin/TSO1-like CXC domain; supporting cDNA gi\|7767426\|gb\|AF205142.1\|AF205142 | At3g22760 | 122.027355 | 165.15 | 104.389046 | 161.999 | 72.8740144 | 167.846 | 0.605813568 | 0.08294 |
| 26964 | 42 | hypothetical protein | At3g19516 | 148.048948 | 252.22 | 175.81942 | 211.75 | 30.6213957 | 122.463 | 0.555781838 | 0.082928 |
| 28916 | 45 | DNA-directed RNA polymerase family protein similar to SP\|P38420 DNA-directed RNA polymerase II 135 kDa polypeptide (EC 2.7.7.6) (RNA polymerase II subunit 2) {Arabidopsis thaliana}; contains Pfam profiles PF04560: RNA polymerase Rpb2 domain 7, PF04561: RNA polymerase Rpb2 domain 2, PF04565: RNA po | At3g18090 | 100.466943 | 143.183 | 186.840698 | 233.507 | 51.1291327 | 110.898 | 0.654288295 | 0.081249 |
| 2932 | 5 | synaptobrevin-related similar to SYNAPTOBREVIN-RELATED PROTEIN GB:P47192 from [Arabidopsis thaliana] (Proc. Natl. Acad. Sci. U.S.A. (1992) 89(9), 3894-3898); contains Pfam profile PF00957: synaptobrevin | At3g24890 | 193.272549 | 248.977 | 93.6019919 | 141.107 | 66.2181754 | 136.213 | 0.641914727 | 0.079872 |
| 5227 | 9 | syntaxin-related protein KNOLLE (KN) / syntaxin 111 (SYP111) identical to SP\|Q42374 Syntaxin-related protein KNOLLE (Syntaxin 111) (AtSYP111) {Arabidopsis thaliana}; BAC F22O13 has a deletion of a cytosine at position 7887 | At1g08560 | 320.967561 | 522.158 | 222.949445 | 305.697 | 48.8630958 | 208.194 | 0.526236426 | 0.079849 |
| 21511 | 34 | expressed protein | At1g23230 | 153.705909 | 175.347 | 187.489196 | 276.495 | 42.2505568 | 103.186 | 0.654711349 | 0.07956 |
| 16311 | 26 | caleosin-related family protein similar to caleosin GB:AAF13743 GI:6478218 from [Sesamum indicum]; similar to Ca+2-binding EF hand protein GB:AAB71227 [Glycine max]; contains Pfam profile PF05042: Caleosin related protein | At1g23240 | 59.4574519 | 115.535 | 139.981544 | 197.975 | 103.670672 | 248.093 | 0.546521387 | 0.07497 |
| 26379 | 41 | expressed protein | At3g57860 | 152.740087 | 252.498 | 209.359669 | 236.309 | 48.8218576 | 180.922 | 0.586907857 | 0.073656 |
| 23722 | 37 | 4-coumarate--CoA ligase 2 / 4-coumaroyl-CoA synthase 2 (4CL2) identical to SP\|Q9S725 4-coumarate--CoA ligase 2 (EC 6.2.1.12) (4CL 2) (4-coumaroyl-CoA synthase 2) {Arabidopsis thaliana} | At3g21240 | 125.994394 | 163.72 | 135.201461 | 259.894 | 32.027617 | 93.164 | 0.544522287 | 0.07176 |
| 18486 | 29 | cyclin family low similarity to microtubule-binding protein TANGLED1 [Zea mays] GI:11228986; contains Pfam profile PF00134: Cyclin, N-terminal domain | At3g05330 | 216.643745 | 308.641 | 128.925212 | 150.76 | 73.0760814 | 180.629 | 0.653887004 | 0.069654 |
| 6521 | 11 | ethylene-responsive protein, putative similar to ethylene-inducible ER33 protein [Lycopersicon esculentum] gi\|5669656\|gb\|AAD46413; identical to bHLH transcription factor (bHLH-alpha gene) | At1g05710 | 128.029788 | 145.781 | 92.8467388 | 133.231 | 64.4201916 | 164.201 | 0.655814772 | 0.0693 |
| 22229 | 35 | no apical meristem (NAM) family protein similar to jasmonic acid 2 GI:6175246 from [Lycopersicon esculentum]; similar to NAC2 (GI:6456751) {Arabidopsis thaliana} | At1g65910 | 161.743735 | 242.651 | 177.430308 | 283.486 | 81.1731941 | 143.909 | 0.61883866 | 0.0689 |
| 21842 | 34 | zinc finger (C3HC4-type RING finger) family protein (ATL5) identical to RING-H2 zinc finger protein ATL5 [Arabidopsis thaliana] gi\|4928401\|gb\|AAD33583 | At3g62690 | 119.708525 | 200.404 | 103.200399 | 184.239 | 58.9561354 | 130.818 | 0.536051041 | 0.065772 |
| 13399 | 21 | hypothetical protein | At4g00640 | 120.076458 | 191.247 | 181.498158 | 236.881 | 34.0070486 | 186.429 | 0.525491069 | 0.06555 |
| 6566 | 11 | beta-1,3-glucanase-related similar to beta-1,3-glucanase GI:15150341 from [Camellia sinensis]; C-terminal homology only | At1g26450 | 74.8507177 | 116.843 | 115.317909 | 149.165 | 56.277717 | 174.051 | 0.579013107 | 0.065424 |
| 6522 | 11 | jacalin lectin family protein (RTM1) identical to gi:6503088 (GB:AAF14583) from [Arabidopsis thaliana] (Proc. Natl. Acad. Sci. U.S.A. 97 (1), 489-494 (2000)); contains Pfam profile PF01419 jacalin-like lectin domain | At1g05760 | 65.1229695 | 114.062 | 97.0372783 | 121.495 | 85.1485528 | 167.95 | 0.625541571 | 0.06512 |
| 7015 | 11 | cytochrome P450 family protein similar to Cytochrome P450 91A1 (SP:Q9FG65) {Arabidopsis thaliana} ; cytochrome P450, Helianthus tuberosus, EMBL:HTCYP81L | At5g10600 | 94.1104827 | 138.012 | 94.2520832 | 140.257 | 59.6118222 | 106.225 | 0.638360262 | 0.064288 |
| 23798 | 37 | cyclic nucleotide-binding domain-containing protein contains Pfam profile: PF00027 cyclic nucleotide-binding domain | At4g00510 | 112.26645 | 153.144 | 223.82898 | 274.899 | 32.9987756 | 85.253 | 0.644789697 | 0.0627 |
| 30035 | 47 | zinc knuckle (CCHC-type) family protein contains Pfam domain, PF00098: Zinc knuckle | At1g65660 | 191.384756 | 264.875 | 97.4403986 | 151.327 | 66.5109664 | 155.858 | 0.597731493 | 0.06132 |
| 6693 | 11 | bifunctional dihydrofolate reductase-thymidylate synthase 1 / DHFR-TS (THY-1) identical to GP:289193:L08593 [SP\|Q05762] | At2g16370 | 80.277721 | 132.526 | 86.8732284 | 145.828 | 56.5416412 | 172.65 | 0.509655835 | 0.060844 |
| 22041 | 34 | disease resistance-responsive protein-related / dirigent protein-related similar to dirigent protein [Thuja plicata] gi\|6694699\|gb\|AAF25360; similar to pathogenesis-related protein [Pisum sativum] gi\|4585273\|gb\|AAD25355 | At5g49040 | 95.101975 | 155.438 | 127.937328 | 233.798 | 47.1310931 | 96.773 | 0.54869087 | 0.058956 |
| 19650 | 31 | COP1-interacting protein-related similar to COP1-Interacting ProteinI 7 (CIP7) [Arabidopsis thaliana] GI:3327870 | At1g72410 | 270.755481 | 303.155 | 89.8607803 | 173.886 | 67.4511964 | 175.868 | 0.597812832 | 0.0585 |
| 18746 | 29 | ferric-chelate reductase, putative similar to ferric-chelate reductase (FRO1) [Pisum sativum] GI:15341529; contains Pfam profile PF01794: Ferric reductase like transmembrane component | At5g23990 | 150.223386 | 176.89 | 96.3648799 | 208.066 | 56.3643171 | 147.181 | 0.565117467 | 0.057132 |
| 7615 | 12 | peroxidase, putative identical to class III peroxidase ATP32 [Arabidopsis thaliana] gi\|17530547\|gb\|AAL40837; identical to cDNA class III peroxidase ATP32 GI:17530546 | At4g33420 | 107.818105 | 134.171 | 137.086407 | 218.775 | 88.4785342 | 241.966 | 0.598620502 | 0.056784 |
| 27613 | 43 | protein kinase family protein contains protein kinase domain, Pfam:PF00069 | At3g18750 | 150.13782 | 181.779 | 163.584 | 231.724 | 36.5431955 | 100.095 | 0.632321468 | 0.056576 |
| 21526 | 34 | expressed protein | At1g31050 | 95.0110615 | 146.171 | 90.8932782 | 175.694 | 56.3457599 | 115.496 | 0.551732304 | 0.055968 |
| 6780 | 11 | glutathione S-transferase, putative identical to glutathione S-transferase GB:AAB09584 from [Arabidopsis thaliana] | At3g03190 | 103.251036 | 130.065 | 69.3908716 | 143.049 | 51.4899666 | 115.561 | 0.57483057 | 0.05304 |
| 19953 | 31 | ABC transporter family protein Bactrocera tryoni membrane transporter (white) gene, PID:g3676298 | At4g25750 | 147.080986 | 166.085 | 180.511868 | 261.943 | 34.8008832 | 120.554 | 0.621125915 | 0.0528 |
| 25112 | 39 | zinc finger (C3HC4-type RING finger) family protein contains Pfam profile: PF00097: Zinc finger, C3HC4 type (RING finger) | At4g09130 | 184.96947 | 263.617 | 180.542142 | 214.699 | 35.8936945 | 172.029 | 0.583739041 | 0.052038 |
| 20033 | 31 | expressed protein | At5g17210 | 156.743491 | 210.095 | 149.774341 | 211.843 | 45.6279621 | 133.867 | 0.597970626 | 0.050286 |
| 14721 | 23 | gamma interferon responsive lysosomal thiol reductase family protein / GILT family protein similar to SP\|P13284 Gamma-interferon inducible lysosomal thiol reductase precursor {Homo sapiens}; contains Pfam profile PF03227: Gamma interferon inducible lysosomal thiol reductase (GILT) | At4g12870 | 89.3230829 | 166.419 | 154.952764 | 199.7 | 67.8759495 | 177.258 | 0.565195194 | 0.04995 |
| 24320 | 38 | pectate lyase family protein similar to pectate lyase P59 SP:P15722 from [Lycopersicon esculentum] | At3g01270 | 175.634254 | 224.859 | 228.137428 | 394.831 | 45.6052811 | 142.822 | 0.559404003 | 0.049284 |
| 10157 | 16 | hypothetical protein low similarity to chaperonin-containing-TCP1 theta subunit from Tetrahymena pyriformis [GI:4959731], Homo sapiens [SP\|P50990] | At4g08640 | 73.0805777 | 142.127 | 109.817627 | 170.281 | 39.0937757 | 162.357 | 0.466633694 | 0.049248 |
| 13212 | 21 | kinesin motor protein-related | At2g22610 | 139.71806 | 197.612 | 97.6746227 | 141.012 | 34.4771637 | 103.473 | 0.577633583 | 0.047736 |
| 9055 | 14 | expressed protein contains similarity to carboxyl-terminal proteinase contains Pfam profile PF03080: Arabidopsis proteins of unknown function | At5g56530 | 125.987977 | 212.564 | 131.10493 | 204.944 | 66.4697282 | 194.9 | 0.524487459 | 0.044733 |
| 14787 | 23 | cyclin 2b (CYC2b) identical to cyclin 2b protein [Arabidopsis thaliana] GI:509423 | At4g35620 | 132.279193 | 191.102 | 110.290855 | 169.998 | 45.6238383 | 184.585 | 0.529379581 | 0.044574 |
| 17755 | 28 | amino acid permease, putative similar to AUX1 [Arabidopsis thaliana] GI:1531758; contains Pfam profile PF01490: Transmembrane amino acid transporter protein | At2g21050 | 158.007724 | 240.003 | 118.82171 | 171.604 | 37.2586776 | 148.479 | 0.533903675 | 0.042976 |
| 13263 | 21 | expressed protein | At2g42860 | 88.7251927 | 154.657 | 134.19605 | 181.476 | 54.6261286 | 110.818 | 0.602031866 | 0.041292 |
| 7230 | 12 | copper amine oxidase, putative similar to copper amine oxidase [Lens culinaris] gi\|15451834\|gb\|AAB34918 | At1g31710 | 75.0239882 | 132.517 | 66.7968798 | 126.393 | 67.9625496 | 168.348 | 0.499444827 | 0.040404 |
| 448 | 1 | glycoside hydrolase family 28 protein / polygalacturonase (pectinase) family protein weak similarity to SP\|P27644 Polygalacturonase (EC 3.2.1.15) (Pectinase) {Agrobacterium tumefaciens}; contains PF00295: Glycosyl hydrolases family 28 | At4g23500 | 136.22591 | 154.357 | 86.0367139 | 153.245 | 32.5740226 | 106.762 | 0.583026386 | 0.0396 |
| 25735 | 40 | glutaredoxin family protein contains INTERPRO Domain IPR002109, Glutaredoxin (thioltransferase) | At3g62960 | 165.460495 | 308.803 | 152.765079 | 278.483 | 68.8532938 | 194.435 | 0.47949797 | 0.039072 |
| 1908 | 3 | glycosyl hydrolase family 17 protein similar to elicitor inducible chitinase Nt-SubE76 GI:11071974 from [Nicotiana tabacum] | At5g55180 | 218.433137 | 319.098 | 114.757046 | 237.551 | 185.245939 | 464.839 | 0.522044417 | 0.037422 |
| 9611 | 15 | beta-Ig-H3 domain-containing protein / fasciclin domain-containing protein contains Pfam profile PF02469: Fasciclin domain | At5g06390 | 62.0511616 | 160.048 | 85.1045977 | 152.401 | 48.9579436 | 172.515 | 0.409972797 | 0.03572 |
| 6938 | 11 | disease resistance protein (TIR class), putative contains Pfam profile PF01582: TIR domain | At4g19925 | 73.3907533 | 115.391 | 75.2910873 | 113.462 | 52.4570014 | 106.175 | 0.597886491 | 0.034914 |
| 5133 | 8 | glycine-rich protein | At5g46730 | 65.2780573 | 103.092 | 98.4697098 | 125.41 | 69.0038131 | 158.941 | 0.617510531 | 0.034452 |
| 30106 | 47 | FAD-binding domain-containing protein / cytokinin oxidase family protein similar to cytokinin oxidase, Zea mays [SP\|Q9T0N8] [gi:3441978] | At2g19500 | 188.718316 | 279.67 | 87.8435853 | 105.976 | 31.1018203 | 126.864 | 0.582949591 | 0.033152 |
| 21913 | 34 | expressed protein hypothetical protein T29E15.27 - Arabidopsis thaliana,PID:g3738334 | At4g30740 | 53.8582477 | 140.664 | 118.984233 | 171.442 | 47.512546 | 98.64 | 0.51952746 | 0.02976 |
| 21559 | 34 | expressed protein | At1g54840 | 81.6178934 | 110.985 | 113.227419 | 137.509 | 40.0484391 | 97.697 | 0.656246307 | 0.0273 |
| 5497 | 9 | expressed protein | At3g12320 | 354.810927 | 717.821 | 136.861743 | 177.112 | 115.65242 | 375.338 | 0.525052937 | 0.026752 |
| 6722 | 11 | no apical meristem (NAM) family protein contains Pfam PF02365: No apical meristem (NAM) domain; | At2g27300 | 99.011257 | 224.122 | 55.9747719 | 87.157 | 54.572519 | 90.058 | 0.563324652 | 0.0265 |
| 10945 | 17 | vegetative storage protein 2 (VSP2) identical to SP\|O82122 Vegetative storage protein 2 precursor {Arabidopsis thaliana}; contains Pfam profile PF03767: HAD superfamily (subfamily IIIB) phosphatase | At5g24770 | 246.1254 | 379.255 | 420.532582 | 675.852 | 61.8737353 | 112.911 | 0.606394457 | 0.025912 |
| 3181 | 5 | expressed protein similar to unknown protein (gb\|AAD10667.1) | At5g44040 | 126.294944 | 183.578 | 139.750507 | 163.213 | 75.148299 | 174.315 | 0.65843863 | 0.02574 |
| 25661 | 40 | expressed protein similar to GB:AAD49756 from [Arabidopsis thaliana] | At3g17680 | 155.102769 | 273.759 | 151.692747 | 282.395 | 106.72642 | 244.455 | 0.513440397 | 0.0248 |
| 10280 | 16 | expressed protein various predicted proteins | At5g19260 | 53.2624967 | 112.293 | 58.9193031 | 118.69 | 45.4836286 | 152.962 | 0.422694333 | 0.024679 |
| 6803 | 11 | expressed protein weak similarity to SP\|Q9UKL6 Phosphatidylcholine transfer protein (PC-TP) {Homo sapiens} | At3g13062 | 65.7572251 | 88.128 | 71.0049462 | 116.217 | 32.9616613 | 72.183 | 0.604588258 | 0.022134 |
| 27373 | 43 | expressed protein | At1g29950 | 257.971969 | 352.502 | 270.791702 | 394.071 | 33.3596095 | 122.954 | 0.563437927 | 0.021672 |
| 23540 | 37 | expressed protein contains Pfam profile PF01027: Uncharacterized protein family UPF0005 | At1g68600 | 234.821104 | 414.735 | 499.805478 | 603.316 | 28.8048548 | 88.155 | 0.573792856 | 0.021627 |
| 21972 | 34 | paired amphipathic helix repeat-containing protein low similarity to transcriptional repressor SIN3B [Mus musculus] GI:2921547; contains Pfam profile PF02671: Paired amphipathic helix repeat | At5g15030 | 170.515287 | 201.191 | 198.228449 | 258.471 | 28.2893778 | 140.441 | 0.605296367 | 0.021 |
| 6781 | 11 | DNA helicase (RECQI1) identical to DNA Helicase [Arabidopsis thaliana] GI:10944747 | At3g05740 | 63.8148841 | 107.25 | 71.3443321 | 96.424 | 45.8877625 | 99.759 | 0.598299664 | 0.0209 |
| 7062 | 11 | chromosome condensation family protein contains pfam profile: PF04154 chromosome condensation protein 3, C-terminal region | At5g37630 | 76.1438291 | 115.465 | 64.0642657 | 102.284 | 40.7123735 | 112.111 | 0.549644772 | 0.0198 |
| 17078 | 27 | E3 ubiquitin ligase SCF complex subunit SKP1/ASK1 (At14), putative E3 ubiquitin ligase; similar to Skp1 homolog Skp1b GI:3068809, UIP2 GI:3719211 from [Arabidopsis thaliana] | At2g03170 | 287.844017 | 324.118 | 285.382109 | 386.254 | 28.8543406 | 100.412 | 0.638096404 | 0.01968 |
| 28995 | 45 | zinc finger (C3HC4-type RING finger) family protein contains Pfam profile: PF00097 zinc finger, C3HC4 type (RING finger) | At4g00305 | 353.662208 | 420.05 | 706.305064 | 849.131 | 21.8191105 | 84.939 | 0.643543311 | 0.01935 |
| 23446 | 37 | hypothetical protein | At1g14680 | 237.470432 | 294.987 | 247.485611 | 291.389 | 21.3613669 | 85.58 | 0.634652548 | 0.019197 |
| 10769 | 17 | cell division control protein 2 homolog B (CDC2B) identical to cell division control protein 2 homolog B [Arabidopsis thaliana] SWISS-PROT:P25859 | At3g54180 | 95.6025343 | 147.14 | 59.1806143 | 131.722 | 37.0937249 | 138.212 | 0.455801865 | 0.018564 |
| 1191 | 2 | glycosyl transferase family 2 protein similar to beta-(1-3)-glucosyl transferase GB:AAC62210 GI:3687658 from [Bradyrhizobium japonicum], cellulose synthase from Agrobacterium tumeficiens [gi:710492] and Agrobacterium radiobacter [gi:710493]; contains Pfam glycosyl transferase, group 2 family protein domain PF00 | At5g22740 | 200.645102 | 244.174 | 138.117312 | 222.387 | 96.0704794 | 242.466 | 0.613006621 | 0.017958 |
| 22805 | 36 | E3 ubiquitin ligase SCF complex subunit, putative similar to Skp1 GI:4959710 from [Medicago sativa] | At1g20140 | 170.562348 | 274.427 | 259.062972 | 358.088 | 89.2290687 | 159.788 | 0.634468362 | 0.017856 |
| 6778 | 11 | expressed protein | At3g02950 | 67.1818937 | 82.14 | 36.7476847 | 80.008 | 56.2282312 | 143.841 | 0.55603352 | 0.017732 |
| 24448 | 38 | expressed protein similar to A. thaliana hypothetical protein T6B20.12 (1946366) | At4g02800 | 129.140003 | 290.139 | 136.739054 | 201.158 | 13.8147836 | 205.187 | 0.39739475 | 0.01708 |
| 6908 | 11 | equilibrative nucleoside transporter, putative (ENT6) identical to putative equilibrative nucleoside transporter ENT6 [Arabidopsis thaliana] GI:16518997; contains similarity to SWISS-PROT:O54699 equilibrative nucleoside transporter 2 (Equilibrative nitrobenzylmercaptopurine riboside-insensitive nucleoside transporter, E | At4g05110 | 69.1509739 | 90.868 | 56.306191 | 96.298 | 46.3063299 | 85.713 | 0.628653699 | 0.015824 |
| 21910 | 34 | expansin-related similar to blight-associated protein p12 precursor [Citrus jambhiri] gi\|4102727\|gb\|AAD03398; similar to beta-expansin [Oryza sativa] gi\|8118428\|gb\|AAF72986; expansin-related gene, PMID:11641069, www.bio.psu.edu/expansins | At4g30380 | 155.811895 | 214.45 | 243.648797 | 294.605 | 26.98419 | 79.37 | 0.631193457 | 0.015444 |
| 6947 | 11 | expressed protein probable membrane protein YPL012w, Saccharomyces cerevisiae, PIR2:S59681 | At4g23540 | 51.0762935 | 85.598 | 58.587884 | 108.542 | 78.7236474 | 109.856 | 0.617692931 | 0.015225 |
| 7078 | 11 | hypothetical protein | At5g44590 | 79.4969341 | 98.45 | 56.8001329 | 108.72 | 52.4240109 | 92.285 | 0.632665331 | 0.01512 |
| 14256 | 22 | mitogen-activated protein kinase kinase (MAPKK), putative (MKK6) similar to NQK1 MAPKK [Nicotiana tabacum] gi\|12718822\|dbj\|BAB32405; mitogen-activated protein kinase kinase (MAPKK) family, PMID:12119167 | At5g56580 | 95.2132104 | 155.086 | 157.62961 | 193.281 | 77.0225733 | 191.643 | 0.610463667 | 0.01449 |
| 11716 | 19 | importin alpha-2 subunit, putative similar to importin alpha-2 subunit (Karyopherin alpha-2 subunit) (KAP alpha) SP:O04294 from [Arabidopsis thaliana] | At1g02690 | 144.651991 | 189.694 | 93.8569297 | 158.542 | 110.27084 | 192.559 | 0.642404937 | 0.014268 |
| 7476 | 12 | cyclopropane fatty acid synthase-related similar to cyclopropane synthase [Sterculia foetida] GI:21069167 | At3g23480 | 55.9289372 | 90.07 | 64.4737595 | 89.054 | 45.5021857 | 102.712 | 0.59598072 | 0.014112 |
| 3195 | 5 | expressed protein | At5g50335 | 95.5426383 | 227.299 | 165.932615 | 367.436 | 64.6531872 | 82.071 | 0.553235463 | 0.01332 |
| 22216 | 35 | expressed protein | At1g58280 | 156.193731 | 203.41 | 146.122357 | 204.623 | 22.9242931 | 85.649 | 0.583211853 | 0.013248 |
| 23623 | 37 | heme oxygenase 2 (HO2) similar to heme oxygenase 2 [Arabidopsis thaliana] gi\|4530595\|gb\|AAD22109 | At2g26550 | 210.24878 | 265.998 | 104.749146 | 127.82 | 21.5036385 | 78.176 | 0.62832901 | 0.013104 |
| 15680 | 25 | subtilase family protein Strong similarity to gb\|U80583 proteinase TMP from Lycopersicon esculentum and is a member of the PF\|00082 subtilase family | At1g30600 | 160.588599 | 229.807 | 159.070009 | 180.779 | 27.8790581 | 102.245 | 0.617127012 | 0.013041 |
| 28861 | 45 | expressed protein contains Pfam profile PF04852: Protein of unknown function (DUF640) | At2g42610 | 104.331303 | 191.435 | 127.637776 | 153.887 | 22.6067593 | 134.947 | 0.513981515 | 0.012992 |
| 22436 | 35 | expressed protein contains Pfam PF03138: Plant protein family. The function of this family of plant proteins is unknown; | At3g26370 | 165.150319 | 251.87 | 135.548814 | 178.267 | 46.39293 | 112.194 | 0.609857546 | 0.012264 |
| 27941 | 43 | cytochrome P450, putative similar to SP\|O64636 Cytochrome P450 76C1 (EC 1.14.-.-) {Arabidopsis thaliana}; contains Pfam profile PF00067: Cytochrome P450 | At1g33720 | 98.6176549 | 119.425 | 79.7923322 | 129.831 | 70.7420016 | 152.28 | 0.634969631 | 0.011914 |
| 6644 | 11 | hypothetical protein | At1g70020 | 64.919751 | 77.079 | 39.6746889 | 98.869 | 59.279855 | 92.619 | 0.627858282 | 0.011687 |
| 30367 | 47 | hypothetical protein | At4g31960 | 147.89386 | 165.453 | 91.57205 | 132.103 | 31.5142019 | 121.903 | 0.61519259 | 0.011648 |
| 7477 | 12 | phragmoplast-associated kinesin-related protein, putative similar to kinesin like protein GB:CAB10194 from [Arabidopsis thaliana] | At3g23670 | 51.2025029 | 87.009 | 57.3657761 | 85.833 | 65.3727931 | 124.961 | 0.593320314 | 0.011616 |
| 9387 | 15 | epoxide hydrolase, putative similar to epoxide hydrolase from [Glycine max] GI:2764806, [Arabidopsis thaliana] GI:1109600; contains Pfam profile PF00561: hydrolase, alpha/beta fold family | At3g05600 | 81.9515996 | 140.091 | 47.1953528 | 93.364 | 45.2856854 | 117.099 | 0.492405545 | 0.01161 |
| 11973 | 19 | ABA-responsive protein-related similar to ABA-inducible protein [Fagus sylvatica] GI:3901016, cold-induced protein kin1 [Brassica napus] GI:167146 | At3g02480 | 105.527084 | 190.548 | 113.789876 | 467.732 | 56.4674125 | 96.589 | 0.460567944 | 0.011468 |
| 6865 | 11 | disease resistance protein (TIR-NBS-LRR class), putative domain signature TIR-NBS-LRR exists, suggestive of a disease resistance protein. | At3g51560 | 44.3454832 | 70.984 | 72.0246973 | 84.865 | 33.3204333 | 77.651 | 0.634175792 | 0.011088 |
| 6916 | 11 | subtilase family protein contains similarity to subtilase; SP1 GI:9957714 from [Oryza sativa] | At4g10510 | 51.7094796 | 76.174 | 64.4371122 | 94.298 | 35.7328656 | 66.359 | 0.633548885 | 0.010948 |
| 8670 | 14 | kinesin motor protein-related | At2g28620 | 158.287951 | 244.591 | 80.1301247 | 152.634 | 110.161559 | 283.68 | 0.520155378 | 0.01081 |
| 895 | 2 | protease inhibitor/seed storage/lipid transfer protein (LTP) family protein contains Pfam protease inhibitor/seed storage/LTP family domain PF00234 | At2g37870 | 122.890499 | 275.433 | 84.8018591 | 577.631 | 93.1631892 | 164.366 | 0.386595019 | 0.010656 |
| 1540 | 3 | xanthine/uracil permease family protein contains Pfam profile: PF00860 permease family | At2g34190 | 167.608193 | 218.253 | 97.3161164 | 179.354 | 65.869713 | 202.591 | 0.545227497 | 0.01053 |
| 6933 | 11 | pentatricopeptide (PPR) repeat-containing protein contains Pfam profile PF01535: PPR repeat | At4g19440 | 86.4512848 | 141.921 | 60.8361163 | 117.982 | 76.9730875 | 132.43 | 0.568675251 | 0.01045 |
| 22391 | 35 | pectate lyase family protein simliar to style development-specific protein 9612 SP:P24396 from [Lycopersicon esculentum] | At3g09540 | 116.011019 | 148.474 | 160.66815 | 222.776 | 25.9449884 | 124.197 | 0.570489032 | 0.01026 |
| 7103 | 11 | DegP protease family contains similarity to DegP2 protease [Arabidopsis thaliana] gi\|13172275\|gb\|AAK14061 | At5g54745 | 59.0007451 | 96.608 | 47.4821578 | 99.982 | 37.9246738 | 77.547 | 0.524894762 | 0.009828 |
| 19463 | 30 | myb family transcription factor (MYB59) contains PFAM profile: myb DNA binding domain PF00249 | At5g59780 | 123.776104 | 169.514 | 134.425494 | 198.19 | 29.8791088 | 109.255 | 0.560642813 | 0.009828 |
| 20470 | 32 | zinc finger (GATA type) family protein similar to zinc finger protein ZIM gi:8918533 from [Arabidopsis thaliana]; contains Pfam PF00320: GATA zinc finger | At3g21175 | 106.937848 | 119.955 | 151.832963 | 246.975 | 60.8572146 | 134.405 | 0.653014475 | 0.009792 |
| 24237 | 38 | hypothetical protein | At2g14340 | 91.4044681 | 128.979 | 179.662606 | 204.254 | 27.5986386 | 86.403 | 0.635899529 | 0.009729 |

| | | | | | | | | | | | |
|---|---|---|---|---|---|---|---|---|---|---|---|
| 15014 | 24 | lysine and histidine specific transporter, putative similar to lysine and histidine specific transporter GI:2576361 from [Arabidopsis thaliana]; contains Pfam profile PF01490: Transmembrane amino acid transporter protein | At1g24400 | 79.9889368 | 124.85 | 109.275884 | 180.913 | 59.0262403 | 128.955 | 0.567477412 | 0.00966 |
| 9912 | 16 | phosphoglycerate/bisphosphoglycerate mutase family protein similar to SP\|P31217 Phosphoglycerate mutase 1 (EC 5.4.2.1) {Escherichia coli O157:H7}; contains Pfam profile PF00300: phosphoglycerate mutase family | At1g78050 | 50.6559521 | 69.412 | 31.3621246 | 132.688 | 43.0773819 | 84.601 | 0.491776492 | 0.0096 |
| 15551 | 24 | actin-related protein, putative (ARP8) strong similarity to actin-related protein 8A (ARP8) [Arabidopsis thaliana] GI:21427473; contains Pfam profile PF00022: Actin; supporting cDNA gi\|21427470\|gb\|AF507916.1\| | At5g56180 | 218.014935 | 272.876 | 243.280721 | 273.006 | 35.1885219 | 144.774 | 0.64437644 | 0.00957 |
| 15316 | 24 | pectate lyase family protein similar to pectate lyase 2 GP:6606534 from [Musa acuminata] | At3g53190 | 77.8604905 | 133.296 | 160.789245 | 260.618 | 53.205474 | 159.786 | 0.511350176 | 0.009177 |
| 6986 | 11 | zinc finger protein-related similar to lateral root primordium 1 (LRP1) [Arabidopsis thaliana] GI:1483241; contains Pfam profile PF05142: Domain of unknown function (DUF702), TIGR01624: LRP1 C-terminal domain, TIGR01623: putative zinc finger domain, LRP1 type | At4g36260 | 57.8862521 | 68.025 | 35.5112366 | 106.854 | 32.7039228 | 71.288 | 0.547349166 | 0.009072 |
| 6646 | 11 | disease resistance protein (TIR-NBS-LRR class), putative domain signature TIR-NBS-LRR exists, suggestive of a disease resistance protein. | At1g72860 | 39.2853428 | 70.368 | 67.2031869 | 94.542 | 46.4053014 | 80.171 | 0.615980712 | 0.008892 |
| 7086 | 11 | forkhead-associated domain-containing protein / FHA domain-containing protein | At5g47790 | 59.9344806 | 93.966 | 66.0591537 | 94.096 | 40.9041309 | 98.363 | 0.58524009 | 0.0084 |
| 24599 | 38 | kinesin motor protein-related non-consensus AT donor splice site at exon 12; non-consensus AC acceptor splice site at exon 13 | At5g27000 | 125.652132 | 157.286 | 133.115751 | 188.077 | 23.2108984 | 68.363 | 0.616391224 | 0.008184 |
| 18964 | 30 | leucine-rich repeat family protein similar to receptor kinase GI:498278 from [Petunia integrifolia]; contains protein kinase domain, Pfam:PF00069; contains leucine-rich repeats, Pfam:PF00560 | At1g60630 | 136.938245 | 194.052 | 175.111967 | 227.166 | 39.535024 | 105.612 | 0.616958299 | 0.008052 |
| 22923 | 36 | methyladenine glycosylase family protein similar to SP\|P05100 DNA-3-methyladenine glycosylase I (EC 3.2.2.20) (3-methyladenine-DNA glycosylase I, constitutive) {Escherichia coli}; contains Pfam profile PF03352: Methyladenine glycosylase | At1g80850 | 149.613731 | 197.331 | 227.809196 | 309.33 | 30.3966477 | 113.401 | 0.587564136 | 0.007808 |
| 20847 | 33 | cyclin, putative similar to cyclin A2 [Lycopersicon esculentum] GI:5420276, cyclin [Medicago sativa] GI:1050559; contains Pfam profiles PF00134: Cyclin, N-terminal domain, PF02984: Cyclin, C-terminal domain | At1g15570 | 196.373236 | 322.28 | 201.053478 | 293.256 | 44.7805179 | 122.918 | 0.553075792 | 0.007626 |
| 7535 | 12 | basic helix-loop-helix (bHLH) protein, putative very strong similarity to PIF3 like basic Helix Loop Helix protein 2 (PIL2) [Arabidopsis thaliana] | At3g62090 | 36.2969615 | 81.652 | 53.4604483 | 70.28 | 94.2271337 | 130.192 | 0.642988525 | 0.00759 |
| 30366 | 47 | WRKY family transcription factor identical to WRKY DNA-binding protein 18 (WRKY18) GI:13506730 from [Arabidopsis thaliana] | At4g31805 | 189.944044 | 295.707 | 93.2689795 | 143.731 | 53.9848753 | 92.271 | 0.625440282 | 0.007524 |
| 14799 | 23 | ankyrin repeat family protein contains ankyrin repeat domains, Pfam:PF00023 | At5g02620 | 181.266615 | 255.695 | 163.961627 | 192.828 | 27.8563771 | 85.679 | 0.62811404 | 0.007473 |
| 22358 | 35 | ribonuclease P family protein similar to Ribonuclease P protein subunit p29 (EC 3.1.26.5) (hPOP4) (Swiss-Prot:O95707) [Homo sapiens] | At2g43190 | 126.569823 | 140.725 | 126.13683 | 158.733 | 24.1552522 | 90.262 | 0.653890981 | 0.00708 |
| 15811 | 25 | transport protein-related contains Pfam PF05911: Plant protein of unknown function (DUF869) profile; weak similarity to Intracellular protein transport protein USO1 (Swiss-Prot:P25386) [Saccharomyces cerevisiae] | At2g23360 | 81.7890248 | 138.126 | 119.80322 | 142.906 | 41.9598278 | 94.608 | 0.624660583 | 0.006837 |
| 9034 | 14 | CHP-rich zinc finger protein, putative contains similarity to CHP-rich zinc finger protein | At5g46660 | 308.289936 | 367.956 | 71.0925811 | 97.676 | 65.8408463 | 293.528 | 0.596664657 | 0.006624 |
| 20139 | 31 | expressed protein | At5g67350 | 147.682085 | 304.365 | 113.753229 | 160.95 | 19.4458543 | 83.513 | 0.474941091 | 0.0066 |
| 19550 | 31 | prenylated rab acceptor (PRA1) family protein weak similarity to prenylated Rab acceptor 1 (PRA1) [Homo sapiens] GI:4877285; contains Pfam profile PF03208: Prenylated rab acceptor (PRA1) | At1g17700 | 102.59432 | 140.242 | 101.772747 | 136.596 | 39.1453234 | 99.276 | 0.623641315 | 0.00648 |
| 11733 | 19 | expressed protein contains Pfam profile PF04520: Protein of unknown function, DUF584 | At1g11700 | 101.315113 | 137.516 | 78.4570956 | 145.686 | 63.5954284 | 95.657 | 0.646704932 | 0.006384 |
| 25327 | 39 | amino acid transporter 4, putative (AAP4) identical to amino acid transporter GI:608671 from [Arabidopsis thaliana]; | At5g63850 | 123.441328 | 154.909 | 101.857195 | 192.899 | 21.8500391 | 72.649 | 0.54188635 | 0.006272 |
| 24835 | 39 | cytochrome P450 family protein similar to Cytochrome P450 90A1 (SP:Q42569) [Arabidopsis thaliana] | At1g65670 | 802.646722 | 1019.197 | 286.115055 | 343.317 | 32.7781515 | 92.183 | 0.65882999 | 0.00616 |
| 27444 | 43 | CLE26, putative CLAVATA3/ESR-Related 26 (CLE26); | At1g69970 | 122.09046 | 154.473 | 88.0507222 | 113.509 | 23.7222515 | 89.903 | 0.609982792 | 0.006138 |
| 20711 | 32 | calmodulin-binding protein-related has weak similarity to calmodulin-binding proteins | At5g39380 | 325.092897 | 410.248 | 279.994955 | 391.366 | 20.5943371 | 54.865 | 0.627741321 | 0.00568 |
| 27431 | 43 | zinc finger (C2H2 type) family protein contains Pfam domain, PF00096: Zinc finger, C2H2 type | At1g66140 | 125.098094 | 261.386 | 107.68093 | 149.955 | 27.7759627 | 89.86 | 0.50192872 | 0.005544 |
| 20201 | 32 | cyclin, putative similar to SP\|Q40671 G2/mitotic-specific cyclin 2 (B-like cyclin) (CYCOS2) {Oryza sativa}; contains Pfam profiles PF00134: Cyclin, N-terminal domain, PF02984: Cyclin, C-terminal domain | At1g20590 | 96.5940266 | 159.653 | 65.1620915 | 105.929 | 27.633691 | 108.108 | 0.491928486 | 0.005376 |
| 21036 | 33 | DNA-binding family protein / AT-hook protein 1 (AHP1) identical to AT-hook protein 1 [Arabidopsis thaliana] gi\|2598227\|emb\|CAA10857 | At2g33620 | 192.761294 | 312.447 | 236.687403 | 260.438 | 36.8751627 | 128.085 | 0.604547327 | 0.005368 |
| 24733 | 39 | expressed protein similar to hypothetical protein GB:AAB71979 | At1g10820 | 182.862415 | 238.826 | 220.030407 | 275.344 | 22.7572786 | 90.837 | 0.605103944 | 0.005313 |
| 20760 | 32 | haloacid dehalogenase-like hydrolase family protein low similarity to SP\|P53078 SSM1 protein {Saccharomyces cerevisiae}; contains InterPro accession IPR005834: Haloacid dehalogenase-like hydrolase | At5g59480 | 262.895204 | 346.488 | 346.183171 | 404.445 | 23.9676186 | 68.945 | 0.65410757 | 0.005293 |
| 23382 | 36 | ankyrin repeat family protein contains ankyrin repeats, Pfam:PF00023 | At5g65860 | 185.876466 | 274.115 | 275.020482 | 404.686 | 39.4628572 | 97.738 | 0.587149377 | 0.005106 |
| 7061 | 11 | pentatricopeptide (PPR) repeat-containing protein contains Pfam profile PF01535: PPR repeat | At5g37570 | 42.511169 | 72.455 | 55.6513196 | 92.419 | 40.3907158 | 65.557 | 0.6016681 | 0.005096 |
| 15521 | 24 | cysteine proteinase, putative / thiol protease, putative similar to cysteine proteinase RD21A precursor (thiol protease) GI:435619, SP:P43297 from [Arabidopsis thaliana] | At5g43060 | 79.0017228 | 120.664 | 164.34244 | 189.231 | 25.281054 | 105.357 | 0.587718741 | 0.005088 |
| 24986 | 39 | C2 domain-containing protein similar to cold-regulated gene SRC2 [Glycine max] GI:2055230; contains Pfam profile PF00168: C2 domain | At3g05440 | 178.776654 | 203.739 | 194.783602 | 248.09 | 29.3966224 | 101.694 | 0.650560338 | 0.00507 |
| 26990 | 42 | hypothetical protein similar to At2g04970, At2g15200, At1g32830, At2g14140, At4g03990, At5g34895, At3g47270, At2g02200 | At3g30450 | 64.3368348 | 114.247 | 126.038042 | 140.773 | 20.8087755 | 79.606 | 0.573287793 | 0.00504 |
| 2363 | 4 | high mobility group (HMG1/2) family protein similar to SP\|P40618 High mobility group protein HMG2A {Gallus gallus}; contains Pfam profile PF00505: HMG (high mobility group) box | At4g11080 | 103.325906 | 143.882 | 73.7327803 | 117.464 | 25.6439498 | 142.706 | 0.508510865 | 0.005031 |
| 18663 | 29 | auxin-responsive AUX/IAA family protein similar to SP\|Q38827 Auxin-responsive protein IAA8, SP\|Q38829 Auxin-responsive protein IAA9 from Arabidopsis thaliana; contains Pfam profile: PF02309: AUX/IAA family | At4g29080 | 115.670895 | 162.14 | 105.110838 | 167.446 | 23.5366798 | 92.202 | 0.532134701 | 0.005016 |
| 25960 | 40 | haloacid dehalogenase-like hydrolase family protein low similarity to SP\|P53078 SSM1 protein {Saccharomyces cerevisiae}; contains InterPro accession IPR005834: Haloacid dehalogenase-like hydrolase | At5g59480 | 129.180646 | 172.087 | 245.904996 | 346.369 | 25.5367306 | 80.417 | 0.59272513 | 0.00494 |
| 30011 | 47 | mechanosensitive ion channel domain-containing protein / MS ion channel domain-containing protein contains Pfam profile PF00924: Mechanosensitive ion channel | At1g53470 | 283.186035 | 401.892 | 269.716183 | 322.129 | 11.4394656 | 43.385 | 0.601865976 | 0.00494 |
| 22962 | 36 | expressed protein | At2g24440 | 135.023713 | 208.671 | 137.717378 | 194.72 | 34.6998497 | 65.44 | 0.628192666 | 0.004872 |
| 2463 | 4 | transducin family protein / WD-40 repeat family protein contains 3 WD-40 repeats (PF00400); similar to uncharacterized protein KIAA0007 (GI:1663708) {Homo sapiens} 1.2e-11 | At5g11240 | 57.4263366 | 124.209 | 64.9995686 | 75.55 | 79.6638775 | 154.377 | 0.612783576 | 0.004805 |
| 21730 | 34 | adenylylsulfate kinase, putative similar to adenylylsulfate kinase 1, chloroplast precursor (APS kinase, Adenosine-5'phosphosulfate kinase, ATP adenosine-5'- phosphosulfate 3'-phosphotransferase) [Arabidopsis thaliana] SWISS-PROT:Q43295 | At3g03900 | 59.8842107 | 98.485 | 131.329594 | 186.433 | 31.5946163 | 76.767 | 0.574684133 | 0.004416 |
| 22701 | 35 | expressed protein contains Pfam profile PF04398: Protein of unknown function, DUF538 | At5g54530 | 81.8564078 | 105.377 | 131.490523 | 180.974 | 22.4521162 | 75.119 | 0.600751477 | 0.004408 |
| 7058 | 11 | Ca+2-binding EF hand family protein contains similarity to Ca+2-binding EF hand protein GI:2270994 from [Glycine max] | At5g29560 | 71.1500021 | 95.781 | 46.5309213 | 85.886 | 38.8607801 | 71.917 | 0.608323939 | 0.004257 |
| 27038 | 42 | F-box family protein contains Pfam:PF00646 F-box domain | At3g62230 | 91.6354954 | 122.597 | 85.6638674 | 95.638 | 25.281054 | 86.672 | 0.644949681 | 0.00392 |
| 20583 | 32 | kinesin-like protein A (KATA) | At4g21270 | 125.689566 | 174.488 | 121.14483 | 153.67 | 22.2933493 | 86.445 | 0.588856064 | 0.00384 |
| 14055 | 22 | nodulin MtN21 family protein similar to MtN21 GI:2598575 (root nodule development) from [Medicago truncatula] | At4g08290 | 66.2096536 | 127.699 | 117.433892 | 156.295 | 60.848967 | 107.367 | 0.612193586 | 0.003726 |
| 7068 | 11 | disease resistance protein (TIR-NBS-LRR class), putative domain signature TIR-NBS-LRR exists, suggestive of a disease resistance protein. | At5g38340 | 78.6380686 | 99.189 | 35.7789213 | 92.375 | 43.1928488 | 90.951 | 0.551678448 | 0.003696 |
| 23168 | 36 | F-box family protein contains F-box domain Pfam:PF00646 | At4g14096 | 100.558926 | 142.611 | 162.61205 | 209.609 | 77.0473162 | 166.633 | 0.647764085 | 0.003572 |
| 25933 | 40 | basic helix-loop-helix (bHLH) family protein contains similarity to bHLH DNA-binding protein | At5g46690 | 90.1241916 | 135.31 | 164.068382 | 279.85 | 32.8008325 | 65.418 | 0.584577834 | 0.0031 |
| 15639 | 25 | expressed protein | At1g12180 | 90.9712918 | 119.688 | 88.5159836 | 114.194 | 26.1078791 | 81.118 | 0.619019271 | 0.003034 |
| 18015 | 28 | dem protein-related / defective embryo and meristems protein-related identical to dem GI:2190419 from [Lycopersicon esculentum] | At4g33400 | 89.6268411 | 108.459 | 75.07757 | 113.68 | 31.4131684 | 75.178 | 0.634881907 | 0.002944 |
| 2263 | 4 | WD-40 repeat family protein / mitotic checkpoint protein, putative contains 5 WD-40 repeats (PF00400) (1 weak); similar to testis mitotic checkpoint protein BUB3 (GB:AAC28439,SP\|O43684)[Homo sapiens] | At3g19590 | 73.3447618 | 110.963 | 67.2462076 | 78.134 | 32.5101034 | 154.13 | 0.577520924 | 0.002904 |
| 3822 | 6 | leucine-rich repeat transmembrane protein kinase, putative | At5g43020 | 57.779295 | 91.091 | 88.6769131 | 118.423 | 31.153368 | 78.615 | 0.593131871 | 0.00285 |
| 25932 | 40 | AWPM-19-like membrane family protein similar to ABA induced plasma membrane protein PM 19 [Triticum aestivum] GI:1724112; contains Pfam profile PF05512: AWPM-19-like family | At5g46530 | 92.706136 | 118.248 | 169.696133 | 192.654 | 20.955171 | 72.565 | 0.65120304 | 0.002784 |
| 19862 | 31 | leucine-rich repeat transmembrane protein kinase, putative receptor-like protein kinase (RKL1), Arabidopsis thaliana, EMBL:AF084034 | At3g50230 | 126.701381 | 143.361 | 97.389411 | 131.103 | 18.7303723 | 80.375 | 0.619892123 | 0.002728 |
| 24152 | 38 | caldesmon-related weak similarity to Caldesmon (CDM) (Swiss-Prot:P12957) [Gallus gallus] | At1g52410 | 91.1606059 | 126.087 | 152.965843 | 179.606 | 4.73001695 | 34.048 | 0.571198046 | 0.002684 |
| 20682 | 32 | calcium-dependent protein kinase 19 (CDPK19) identical to calcium-dependent protein kinase [Arabidopsis thaliana] gi\|836942\|gb\|AAA67655 | At5g19450 | 84.1025069 | 107.181 | 175.825793 | 205.765 | 18.309743 | 57.306 | 0.652894584 | 0.002684 |
| 24819 | 39 | expressed protein contains Pfam domain, PF04678: Protein of unknown function, DUF607 | At1g57610 | 103.99118 | 140.678 | 125.011917 | 141.322 | 13.1529111 | 66.203 | 0.607492968 | 0.002655 |
| 7094 | 11 | expressed protein | At5g51180 | 34.1171758 | 85.291 | 35.0396018 | 78.703 | 38.3514888 | 75.338 | 0.451427053 | 0.002576 |
| 6607 | 11 | ubiquitin family protein contains Pfam profile: PF00240 ubiquitin family | At1g53930 | 48.1659908 | 92.07 | 38.2151702 | 59.955 | 30.367781 | 91.055 | 0.498017731 | 0.002508 |
| 18592 | 29 | expressed protein | At3g60990 | 127.294993 | 186.843 | 132.612249 | 162.047 | 29.5698226 | 69.099 | 0.64252825 | 0.002394 |
| 27384 | 43 | light stress-responsive one-helix protein (OHP2) contains similarity to photosystem II 22 kDa protein GI:6006279 from [Arabidopsis thaliana] | At1g34000 | 84.6897014 | 103.751 | 85.2193197 | 117.979 | 33.0400138 | 84.702 | 0.642892727 | 0.00234 |
| 27294 | 42 | hypothetical protein | At1g35150 | 83.6072955 | 102.946 | 94.3190044 | 112.142 | 4.34031634 | 19.203 | 0.626412514 | 0.002322 |
| 6775 | 11 | hypothetical protein | At3g02680 | 43.9401158 | 74.987 | 67.7210292 | 98.527 | 27.4151288 | 63.442 | 0.568477841 | 0.002268 |
| 3085 | 5 | nucleotide-sugar transporter family protein similar to SP\|O77592 UDP N-acetylglucosamine transporter (Golgi UDP-GlcNAc transporter) {Canis familiaris}, SP\|P78382 CMP-sialic acid transporter {Homo sapiens}; contains Pfam profile PF04142: Nucleotide-sugar transporter | At4g35335 | 71.5724826 | 99.48 | 56.6360167 | 96.265 | 43.1309915 | 94.654 | 0.587823515 | 0.00221 |
| 15868 | 25 | hypothetical protein and genefinder; expression supported by MPSS | At2g46640 | 51.6474444 | 99.8 | 69.6697097 | 81.175 | 53.2322788 | 99.154 | 0.637546574 | 0.002187 |
| 30564 | 48 | zinc finger (C3HC4-type RING finger) family protein / BRCT domain-containing protein contains Pfam domain, PF00533: BRCA1 C Terminus (BRCT) domain | At1g04020 | 110.959435 | 158.139 | 140.351204 | 174.793 | 9.55900549 | 72.96 | 0.545210451 | 0.002162 |
| 6829 | 11 | F-box family protein contains F-box domain Pfam:PF00646 | At3g25750 | 57.734373 | 69.535 | 81.6661247 | 106.345 | 19.6025594 | 62.676 | 0.636996071 | 0.00216 |
| 30683 | 48 | glycosyl transferase family 20 protein / trehalose-phosphatase family protein similar to SP\|Q00764 Alpha,alpha-trehalose-phosphate synthase [UDP-forming] 56 kDa subunit (EC 2.4.1.15) (Trehalose-6-phosphate synthase) {Saccharomyces cerevisiae}; contains Pfam profile: PF02358 trehalose-phosphatase | At1g68020 | 125.383669 | 172.514 | 100.713162 | 153.283 | 58.5973635 | 106.271 | 0.64507971 | 0.002145 |
| 21937 | 34 | hydroxyproline-rich glycoprotein family protein | At4g37130 | 52.3897267 | 70.385 | 86.6007637 | 119.302 | 28.2027776 | 68.207 | 0.627904731 | 0.002142 |
| 16183 | 25 | expressed protein | At5g45030 | 87.2352803 | 108.998 | 98.7294276 | 119.447 | 16.1035015 | 47.988 | 0.654155378 | 0.002112 |
| 6621 | 11 | hypothetical protein | At1g62530 | 60.7997635 | 71.115 | 71.4016931 | 102.098 | 20.530418 | 51.021 | 0.652228708 | 0.0021 |
| 7127 | 11 | transducin family protein / WD-40 repeat family protein Similar to (SP:Q13112) Chromatin assembly factor 1 subunit B (CAF-1 subunit B) (CAF-Ip60) [Homo sapiens] | At5g64630 | 69.0386689 | 86.359 | 51.5292947 | 92.935 | 15.8643202 | 82.252 | 0.51559286 | 0.00208 |
| 24917 | 39 | jacalin lectin family protein similar to myrosinase-binding protein homolog [Arabidopsis thaliana] GI:2997767; contains Pfam profile PF01419 jacalin-like lectin domain | At2g25980 | 139.620729 | 236.246 | 166.727702 | 322.318 | 41.7247703 | 145.16 | 0.465238041 | 0.002025 |
| 17924 | 28 | delta 1-pyrroline-5-carboxylate synthetase B / P5CS B (P5CS2) identical to SP\|P54888 | At3g55610 | 96.33626 | 116.9 | 76.2216102 | 109.467 | 29.5595131 | 92.117 | 0.613759921 | 0.002024 |
| 5568 | 9 | expressed protein contains Pfam PF05701: Plant protein of unknown function (DUF827) | At3g51720 | 146.078798 | 199.506 | 70.7420417 | 95.086 | 62.9892275 | 157.167 | 0.62565369 | 0.002016 |
| 24469 | 38 | expressed protein | At4g14840 | 56.5578449 | 79.21 | 128.518905 | 150.098 | 19.8005025 | 77.552 | 0.608525462 | 0.001998 |
| 24742 | 39 | myb family transcription factor (MYB124) contains PFAM profile: PF00249 myb-like DNA binding domain | At1g14350 | 97.338448 | 120.085 | 106.516183 | 127.016 | 10.2023208 | 33.56 | 0.651062143 | 0.001911 |
| 21486 | 34 | protein kinase family protein contains protein kinase domain, Pfam:PF00069 | At1g12680 | 55.1000196 | 72.974 | 98.3533944 | 116.907 | 26.7573801 | 85.828 | 0.636038527 | 0.00188 |
| 6969 | 11 | expressed protein low similarity to SP\|Q13061 Triadin {Homo sapiens} | At4g32970 | 35.3375563 | 64.775 | 40.5749379 | 80.419 | 81.2927848 | 98.287 | 0.625727769 | 0.001872 |
| 21453 | 34 | Control: Stratagene Spotreport Alien Oligo | Alien1 | 78.8423567 | 102.945 | 91.1976102 | 100.545 | 18.1839667 | 74.569 | 0.638918581 | 0.001848 |
| 16506 | 26 | strictosidine synthase family protein similar to strictosidine synthase [Rauvolfia serpentina][SP\|P15324]; contains strictosidine synthase domain PF03088; protein alignments support a CG non-consensus donor splice site. | At2g41300 | 109.447061 | 143.174 | 101.528963 | 113.387 | 19.0355347 | 60.562 | 0.658056175 | 0.001833 |
| 15047 | 24 | jacalin lectin family protein nearly identical to myrosinase-binding protein homolog GI:2997767 from [Arabidopsis thaliana]; contains Pfam profile PF01419 jacalin-like lectin domain; identical to cDNA myrosinase-binding protein homolog GI:2997766 | At1g52040 | 50.9126492 | 124.118 | 294.065926 | 469.386 | 47.1929503 | 65.314 | 0.586413682 | 0.001722 |
| 22012 | 34 | F-box family protein contains F-box domain Pfam:PF00646 | At5g38396 | 62.6715127 | 94.72 | 107.672963 | 130.305 | 15.1715191 | 47.708 | 0.601991007 | 0.00171 |
| 17468 | 27 | expressed protein ; expression supported by MPSS | At5g38320 | 119.806926 | 149.071 | 69.6458093 | 87.677 | 12.1673191 | 47.008 | 0.618956898 | 0.001692 |
| 30196 | 47 | phytochromobilin:ferredoxin oxidoreductase, chloroplast / phytochromobilin synthase (HY2) identical to SP\|Q9SR43 Phytochromobilin:ferredoxin oxidoreductase, chloroplast precursor (EC 1.3.7.4) (Phytochromobilin synthase) (PFB synthase) (PPhiB synthase) {Arabidopsis thaliana}; identical to cDNA for phytochromobili | At3g09150 | 70.6269818 | 105.237 | 41.4018922 | 67.04 | 62.6098364 | 147.257 | 0.571289005 | 0.001596 |
| 26399 | 41 | plastocyanin-like domain-containing protein | At4g01380 | 82.5045678 | 95.715 | 118.909345 | 178.265 | 21.4314718 | 68.288 | 0.614286018 | 0.001596 |
| 21499 | 34 | pentatricopeptide (PPR) repeat-containing protein contains Pfam profile PF01535: PPR repeat | At1g19290 | 48.5403407 | 98.638 | 94.891021 | 114.277 | 23.0686267 | 81.93 | 0.534676905 | 0.0015 |
| 20160 | 32 | expressed protein contains 2 predicted transmembrane domains; expression supported by MPSS | At1g03660 | 73.1500998 | 107.413 | 125.405478 | 150.767 | 30.8523294 | 104.016 | 0.603137302 | 0.001488 |
| 12027 | 19 | kinase interacting family protein similar to kinase interacting protein 1 (GI:13936326) [Petunia integrifolia] | At3g22790 | 77.3898793 | 112.016 | 28.3634192 | 118.758 | 117.811237 | 219.699 | 0.488651807 | 0.001485 |
| 9800 | 16 | expressed protein | At1g20460 | 67.3166596 | 88.824 | 16.4020581 | 45.223 | 73.9214637 | 129.475 | 0.563830277 | 0.00147 |
| 15560 | 24 | haloacid dehalogenase-like hydrolase family protein low similarity to SP\|P53078 SSM1 protein {Saccharomyces cerevisiae}; contains InterPro accession IPR005834: Haloacid dehalogenase-like hydrolase | At5g59480 | 89.3369873 | 123.995 | 128.950706 | 189.084 | 35.3637841 | 83.536 | 0.608600083 | 0.00144 |
| 6551 | 11 | zinc finger (C3HC4-type RING finger) family protein contains Pfam profile: PF00097 zinc finger, C3HC4 type (RING finger) | At1g18780 | 34.6530308 | 55.672 | 48.9066225 | 66.422 | 30.8440818 | 70.469 | 0.598816211 | 0.001404 |
| 25057 | 39 | leucine-rich repeat transmembrane protein kinase, putative similar to Xa21 receptor type precursor, Oryza sativa, PIR:A57676 | At3g47110 | 76.550266 | 111.247 | 98.2832866 | 114.297 | 30.0791139 | 85.285 | 0.633564686 | 0.001344 |
| 7710 | 12 | methyl-CpG-binding domain-containing protein contains Pfam profile PF01429:Methyl-CpG binding domain | At5g35338 | 67.0268059 | 88.421 | 83.6689131 | 131.828 | 23.5387417 | 60.183 | 0.619899996 | 0.00132 |
| 6700 | 11 | expressed protein contains Pfam profile PF03087: Arabidopsis protein of unknown function; expression supported by MPSS | At2g17070 | 46.9648626 | 55.354 | 31.6282159 | 82.121 | 33.281257 | 67.637 | 0.575214762 | 0.00128 |
| 31158 | 48 | CwfJ-like family protein / zinc finger (CCCH-type) family protein contains Pfam domain, PF00642: Zinc finger C-x8-C-x5-C-x3-H type (and similar), PF04677: Protein similar to CwfJ C-terminus 1, PF04676: Protein similar to CwfJ C-terminus 2 | At5g56900 | 96.4646085 | 125.145 | 112.755785 | 130.244 | 10.5301642 | 46.281 | 0.621358906 | 0.001225 |
| 23952 | 37 | copper-binding family protein similar to copper homeostasis factor gi:3168840 from Arabidopsis thaliana; contains Pfam profile PF00403: Heavy-metal-associated domain | At5g52740 | 43.7016015 | 104.319 | 49.5407802 | 78.934 | 27.8275104 | 72.742 | 0.476365457 | 0.001188 |
| 22606 | 35 | expressed protein contains Pfam profile PF04788: Protein of unknown function (DUF620) | At5g06610 | 60.1462556 | 93.189 | 71.0288467 | 114.968 | 24.182057 | 57.557 | 0.561125784 | 0.001178 |
| 10792 | 17 | Dof zinc finger protein DAG1 / Dof affecting germination 1 (DAG1) / transcription factor BBFa (BBFA) identical to SP\|Q43385 DOF zinc finger protein DAG1 (Dof affecting germination 1) (Transcription factor BBFa) (AtBBFa) (rolB domain B factor a) {Arabidopsis thaliana} | At3g61850 | 63.044793 | 107.247 | 350.496399 | 475.807 | 48.2630806 | 119.481 | 0.576140559 | 0.001144 |
| 6856 | 11 | ABC transporter family protein contains Pfam domain, PF00005: ABC transporter | At3g47790 | 53.2004615 | 59.537 | 43.1657429 | 76.787 | 21.51601 | 66.568 | 0.593480247 | 0.001131 |
| 7011 | 11 | protease inhibitor/seed storage/lipid transfer protein (LTP) family protein identical to tapetum-specific protein A9 [Precursor] SP\| Q00762; contains Pfam protease inhibitor/seed storage/LTP family domain PF00234 | At5g07230 | 56.8241681 | 64.995 | 31.3812449 | 82.146 | 32.2915412 | 88.516 | 0.540371124 | 0.001056 |
| 16326 | 26 | expressed protein similar to EST gb\|T43267 | At1g31060 | 86.6555728 | 116.85 | 104.427287 | 122.227 | 18.5159338 | 73.158 | 0.616354498 | 0.001056 |
| 27493 | 43 | F-box family protein contains Pfam:PF00646 F-box domain | At2g16300 | 68.9980252 | 116.863 | 73.2930126 | 112.546 | 28.9718693 | 77.087 | 0.539159541 | 0.00104 |
| 7056 | 11 | calcium-binding EF hand family protein contains INTERPRO:IPR002048 calcium-binding EF-hand domain | At5g28830 | 33.6187557 | 48.362 | 29.7831038 | 91.482 | 68.3233835 | 89.132 | 0.572937095 | 0.001008 |
| 20455 | 32 | expressed protein | At3g17120 | 66.7412304 | 88.522 | 91.262938 | 151.481 | 7.26410188 | 42.111 | 0.509640293 | 0.001008 |
| 11708 | 19 | Control: Stratagene Spotreport Alien Oligo | Alien1 | 64.3079564 | 79.077 | 79.3669048 | 136.744 | 43.4773921 | 116.15 | 0.589319405 | 0.00099 |
| 24942 | 39 | harpin-induced family protein (YLS9) / HIN1 family protein / harpin-responsive family protein similar to harpin-induced protein hin1 ( GI:1619321) [Nicotiana tabacum]; identical to cDNA YLS9 mRNA for hin1 homolog GI:13122295 | At2g35980 | 65.6620333 | 94.618 | 301.167536 | 399.398 | 57.8756957 | 139.739 | 0.620731145 | 0.00099 |
| 21864 | 34 | ankyrin repeat family protein contains ankyrin repeats, Pfam:PF00023 | At4g11000 | 58.0071136 | 101.826 | 67.6429545 | 99.026 | 23.3366747 | 76.218 | 0.519645 | 0.000986 |
| 24844 | 39 | transcription factor CRC (CRABS CLAW) identical to transcription factor CRC (CRABS CLAW) GI:4836698 [Arabidopsis thaliana] | At1g69180 | 61.3784014 | 78.323 | 111.391868 | 122.743 | 19.9510218 | 79.418 | 0.647464648 | 0.000924 |
| 6845 | 11 | DC1 domain-containing protein contains Pfam profile PF03107: DC1 domain | At3g43890 | 56.9632123 | 70.512 | 59.1152865 | 73.632 | 20.3592796 | 62.835 | 0.644903618 | 0.000918 |
| 21742 | 34 | kinesin motor protein-related similar to carboxy-terminal kinesin 2 GB:P79955 [Xenopus laevis] | At3g10310 | 40.0607818 | 75.246 | 55.6560997 | 82.648 | 19.5819403 | 69.033 | 0.496489799 | 0.000864 |
| 20295 | 32 | bHLH family protein contains Pfam profile: PF00010 helix-loop-helix DNA-binding domain | At1g74500 | 86.0063433 | 104.21 | 104.773046 | 196.756 | 34.2709729 | 114.054 | 0.552766739 | 0.000854 |
| 27345 | 43 | expressed protein | At1g15430 | 96.3822515 | 233.914 | 94.736465 | 122.957 | 48.6218525 | 87.979 | 0.57839295 | 0.00084 |
| 14495 | 23 | zinc finger (C3HC4-type RING finger) family protein similar to Pfam domain, PF00097: Zinc finger, C3HC4 type (RING finger) | At2g15530 | 72.62601 | 119.946 | 85.3786558 | 102.895 | 32.7389752 | 68.985 | 0.636611719 | 0.00078 |
| 6730 | 11 | expressed protein | At2g30695 | 68.7263542 | 89.281 | 24.3720499 | 83.34 | 39.9247246 | 80.487 | 0.519418811 | 0.00076 |
| 10023 | 16 | expressed protein ; expression supported by MPSS | At3g04160 | 48.5991671 | 65.193 | 59.5439006 | 85.292 | 21.4417813 | 70.073 | 0.583192108 | 0.000754 |
| 21218 | 33 | hypothetical protein late embryogenesis abundant protein -Picea glauca,PID:g1161171 | At4g13235 | 198.490986 | 281.574 | 96.3361994 | 205.33 | 41.8959087 | 157.703 | 0.479924808 | 0.000744 |
| 11861 | 19 | protein kinase, putative similar to protein kinase APK1B [Arabidopsis thaliana] SWISS-PROT:P46573; contains Pfam profile: PF00069 Eukaryotic protein kinase domain | At1g76370 | 61.4800107 | 77.838 | 63.6786724 | 103.903 | 31.959574 | 76.099 | 0.607561965 | 0.00072 |
| 14876 | 23 | TCP family transcription factor, putative similar to PCF1 (GI:2580438) and PCF2 [(GI:2580440) Oryza sativa] ; similar to unknown protein (emb\|CAB61988.1) | At5g41030 | 72.8559678 | 92.856 | 95.8215438 | 138.223 | 32.6358798 | 73.353 | 0.640922191 | 0.000672 |
| 12460 | 20 | plant defensin-fusion protein, putative (PDF1.5) plant defensin protein family member, personal communication, Bart Thomma (Bart.Thomma@agr.kuleuven.ac.be); similar to antifungal protein 4 [Raphanus sativus] gi\|1655683\|emb\|CAA65983 | At1g55010 | 32.1673478 | 79.358 | 112.037179 | 148.427 | 25.3429112 | 106.936 | 0.465722102 | 0.00066 |
| 10932 | 17 | AAA-type ATPase family protein contains Pfam profile: ATPase family PF00004 | At5g17760 | 175.99363 | 251.005 | 103.47605 | 127.492 | 18.9654298 | 48.738 | 0.633971301 | 0.00066 |
| 19123 | 30 | myb family transcription factor contains Pfam profile: PF00249 myb-like DNA-binding domain | At3g04030 | 52.1458645 | 84.384 | 74.583635 | 82.189 | 19.1468777 | 50.745 | 0.634246542 | 0.000616 |
| 6628 | 11 | hypothetical protein | At1g63205 | 61.4907064 | 79.62 | 38.9465229 | 61.469 | 18.400467 | 54.603 | 0.580961603 | 0.000558 |
| 18700 | 29 | expressed protein | At5g02580 | 105.369857 | 162.985 | 65.4616433 | 93.985 | 43.5619303 | 90.034 | 0.608950201 | 0.00055 |
| 18620 | 29 | ubiquitin-protein ligase, putative similar to SP\|P39940 Ubiquitin--protein ligase RSP5 (EC 6.3.2.-) {Saccharomyces cerevisiae}; contains Pfam profiles PF00240: Ubiquitin family, PF00632: HECT-domain (ubiquitin-transferase) | At4g12570 | 74.3030974 | 108.28 | 78.3375936 | 93.027 | 19.3427589 | 78.551 | 0.589619456 | 0.00054 |
| 15497 | 24 | WD-40 repeat family protein contains 5 WD-40 repeats (PF00400); similar to fizzy1 (GI:3298595) {Xenopus laevis}; WD-repeat protein, carrot, PIR:T14352 | At5g26900 | 39.7473975 | 106.215 | 75.0552699 | 125.838 | 95.2580877 | 140.778 | 0.54910489 | 0.000528 |
| 25100 | 39 | F-box family protein ; similar to SKP1 interacting partner 2 (SKIP2) TIGR_Ath1:At5g67250 | At4g00755 | 93.1585645 | 103.883 | 76.9545562 | 105.273 | 12.4312433 | 100.316 | 0.583895022 | 0.000528 |
| 18164 | 28 | subtilase family protein contains similarity to prepro-cucumisin GI:807698 from [Cucumis melo] | At5g59090 | 117.069894 | 134.69 | 57.9951537 | 91.182 | 46.7248972 | 133.303 | 0.618578024 | 0.000522 |
| 30913 | 48 | expressed protein contains Pfam profile PF04949: Family of unknown function (DUF662) | At3g52900 | 71.5457433 | 98.2 | 47.4216101 | 76.947 | 41.3206363 | 156.846 | 0.536102723 | 0.00051 |
| 27590 | 43 | transcription factor IIB (TFIIB) family protein contains Pfam domain, PF00382: Transcription factor TFIIB repeat | At3g09360 | 90.5081676 | 145.058 | 116.847536 | 170.447 | 22.0191155 | 67.571 | 0.545115642 | 0.000486 |

| | | | | | | | | | | | |
|---|---|---|---|---|---|---|---|---|---|---|---|
| 19572 | 31 | phosphoribulokinase/uridine kinase family protein weak similarity to SP\|Q59190 Uridine kinase (EC 2.7.1.48) (Uridine monophosphokinase) (Cytidine monophosphokinase) {Borrelia burgdorferi}; contains Pfam profile PF00485: Phosphoribulokinase / Uridine kinase family | At1g26190 | 130.854525 | 221.497 | 50.9891454 | 99.945 | 28.3223683 | 64.703 | 0.512891409 | 0.000473 |
| 1322 | 3 | jacalin lectin family protein similar to gi:6503088 (GB:AAF14583) from [Arabidopsis thaliana] (Proc. Natl. Acad. Sci. U.S.A. 97 (1), 489-494 (2000)); contains Pfam profile PF01419 jacalin-like lectin domain | At1g05770 | 65.7957296 | 75.463 | 57.2367138 | 111.566 | 80.1937878 | 151.905 | 0.637614881 | 0.000468 |
| 22249 | 35 | sugar transporter family protein contains Pfam profile: PF00083 sugar (and other) transporter | At1g73220 | 50.2816023 | 79.005 | 53.4445147 | 87.116 | 15.6601913 | 25.396 | 0.622187518 | 0.000442 |
| 30183 | 47 | sugar transporter family protein similar to sugar-porter family proteins 1 and 2 [Arabidopsis thaliana] GI:14585699, GI:14585701; contains Pfam profile PF00083: major facilitator superfamily protein | At3g05150 | 97.0806814 | 134.96 | 50.9190375 | 83.503 | 42.2258139 | 105.971 | 0.575860655 | 0.00044 |
| 22590 | 35 | expressed protein ; expression supported by MPSS | At4g39190 | 1331.23727 | 1890.812 | 74.6346226 | 120.965 | 42.1495233 | 83.424 | 0.608764655 | 0.000416 |
| 24146 | 38 | endo-1,4-beta-glucanase, putative / cellulase, putative similar to endo-beta-1,4-glucanase GI:4972236 from [Fragaria x ananassa] | At1g48930 | 61.583759 | 90.776 | 118.891818 | 153.895 | 24.3800002 | 63.877 | 0.610879035 | 0.000396 |
| 20076 | 31 | myb family transcription factor contains Pfam profile: PF00249 Myb DNA binding domain | At5g41020 | 60.0767335 | 84.099 | 69.092913 | 119.892 | 43.2402727 | 97.542 | 0.577983081 | 0.000396 |
| 14607 | 23 | phosphoesterase identical to phosphoesterase [Arabidopsis thaliana] GI:21630064; contains Pfam profile PF00645: Poly(ADP-ribose) polymerase and DNA-Ligase Zn-finger region | At3g14890 | 66.1486881 | 93.211 | 75.214064 | 90.754 | 13.1013634 | 59.825 | 0.58581182 | 0.00039 |
| 6941 | 11 | WD-40 repeat family protein contains 6 WD-40 repeats (PF00400); similar to fizzy-related protein (GI:5813825) Drosophila melanogaster, PID:g2326419; | At4g22910 | 31.0132807 | 54.554 | 39.9901744 | 69.058 | 29.1223886 | 71.343 | 0.518590399 | 0.000384 |
| 17856 | 28 | expressed protein | At3g16330 | 46.3883638 | 69.609 | 59.5040665 | 92.001 | 46.8609831 | 84.856 | 0.621810285 | 0.000378 |
| 6790 | 11 | protein phosphatase 2C family protein / PP2C family protein similar to protein phosphatase-2c (GI:3608412) [Mesembryanthemum crystallinum]; contains Pfam PF00481 : Protein phosphatase 2C domain | At3g09400 | 46.3498593 | 63.417 | 29.1186723 | 76.937 | 24.4480432 | 51.104 | 0.529248812 | 0.000368 |
| 21360 | 33 | hypothetical protein | At5g32070 | 188.998544 | 268.017 | 153.035951 | 209.812 | 37.4360016 | 90.756 | 0.615686657 | 0.000336 |
| 6341 | 10 | serine/threonine protein kinase, putative similar to serine-threonine protein kinase [Triticum aestivum] gi\|2055374\|gb\|AAB58348 | At4g40010 | 19.8758379 | 60.797 | 52.6786806 | 81.769 | 11.161108 | 49.549 | 0.418983166 | 0.000322 |
| 18281 | 29 | late embryogenesis abundant group 1 domain-containing protein / LEA group 1 domain-containing protein contains Pfam domain, PF03760: Late embryogenesis abundant (LEA) group 1 | At1g32560 | 78.8402176 | 108.119 | 106.16883 | 157.665 | 27.7285388 | 64.873 | 0.61000298 | 0.000312 |
| 28299 | 44 | SIT4 phosphatase-associated family protein contains Pfam profile: PF04499 SIT4 phosphatase-associated protein | At3g45190 | 37.1611748 | 64.892 | 46.07522 | 78.24 | 48.5599953 | 80.562 | 0.588107786 | 0.0003 |
| 19528 | 31 | LOB domain protein 1 / lateral organ boundaries domain protein 1 (LBD1) identical to SP\|Q9LQR0 LOB domain protein 1 {Arabidopsis thaliana} | At1g07900 | 79.0092098 | 90.462 | 103.914224 | 148.447 | 24.4315479 | 70.164 | 0.640537303 | 0.00028 |
| 23331 | 36 | NLI interacting factor (NIF) family protein contains Pfam profile PF03031: NLI interacting factor | At5g45700 | 85.1752866 | 180.082 | 78.2451786 | 184.547 | 51.0734612 | 222.721 | 0.375427182 | 0.00027 |
| 3015 | 5 | beta-fructofuranosidase, putative / invertase, putative / saccharase, putative / beta-fructosidase, putative similar to neutral invertase [Daucus carota] GI:4200165; contains Pfam profile PF04853: Plant neutral invertase | At4g09510 | 120.682905 | 134.51 | 55.4505562 | 88.842 | 14.0560268 | 69.735 | 0.574305133 | 0.000261 |
| 10020 | 16 | vacuolar ATP synthase subunit G 1 (VATG1) / V-ATPase G subunit 1 (VAG1) / vacuolar proton pump G subunit 1 (VMA10) identical to SWISS-PROT:O82628 vacuolar ATP synthase subunit G 1 (V-ATPase G subunit 1, Vacuolar proton pump G subunit 1) [Arabidopsis thaliana] | At3g01390 | 42.6191956 | 63.733 | 20.5288631 | 67.988 | 40.8649547 | 96.573 | 0.464604662 | 0.00026 |
| 17200 | 27 | protein kinase, putative similar to viroid symptom modulation protein [Lycopersicon esculentum] gi\|7672777\|gb\|AAF66637 | At3g12690 | 43.7379669 | 93.923 | 73.157577 | 122.736 | 14.4745942 | 76.076 | 0.41733343 | 0.000258 |
| 10311 | 16 | pollen specific phosphatase, putative / phosphatase and tensin, putative (PTEN1) identical to phosphatase and tensin homolog [Arabidopsis thaliana] GI:21535746 | At5g39400 | 35.8819679 | 51.163 | 68.0795354 | 79.868 | 31.3286302 | 100.918 | 0.621387884 | 0.00024 |
| 2942 | 5 | pentatricopeptide (PPR) repeat-containing protein contains Pfam profile PF01535: PPR repeat | At3g28660 | 108.85131 | 136.284 | 39.8929794 | 65.025 | 20.7860945 | 53.569 | 0.600078753 | 0.00021 |
| 10207 | 16 | expressed protein | At4g31010 | 41.050135 | 46.043 | 45.1893113 | 67.592 | 15.5674054 | 51.527 | 0.620747401 | 0.000208 |
| 21355 | 33 | SSXT protein-related / glycine-rich protein contains weak hit to Pfam profile PF05030: SSXT protein (N-terminal region) | At5g28640 | 96.622905 | 220.242 | 100.915519 | 160.835 | 37.1432107 | 113.959 | 0.4640316 | 0.000204 |
| 31148 | 48 | seed maturation family protein similar to SP\|P09444 Late embryogenesis abundant protein D-34 (LEA D-34) {Gossypium hirsutum}; contains Pfam profile PF04927: Seed maturation protein | At5g53270 | 44.8567382 | 70.279 | 195.424133 | 226.64 | 37.6958021 | 87.878 | 0.643163137 | 0.000201 |
| 18615 | 29 | glycosyltransferase family protein contains Pfam profile: PF00201 UDP-glucoronosyl and UDP-glucosyl transferase | At4g09500 | 68.6589712 | 110.189 | 67.8915188 | 95.391 | 14.9117187 | 34.444 | 0.589248866 | 0.0002 |
| 24353 | 38 | protein kinase family protein contains protein kinase domain, Pfam:PF00069 | At3g14370 | 39.0970983 | 89.679 | 112.425959 | 129.639 | 36.5328859 | 58.28 | 0.6433472 | 0.000188 |
| 1744 | 3 | protein kinase family protein contains Pfam domain, PF00069: Protein kinase domain | At4g23210 | 88.1893377 | 111.423 | 89.9611621 | 122.058 | 30.8255246 | 90.264 | 0.623340871 | 0.000177 |
| 23353 | 36 | expressed protein | At5g55540 | 88.2588598 | 111.602 | 98.5382243 | 113.137 | 24.2872143 | 86.168 | 0.647886141 | 0.000174 |
| 23279 | 36 | expressed protein similar to p53 inducible protein [Homo sapiens] GI:5616320 | At5g18410 | 85.3581832 | 109.986 | 111.164017 | 138.419 | 5.36714652 | 63.209 | 0.554697111 | 0.000168 |
| 30523 | 47 | regulator of chromosome condensation (RCC1) family protein weak similarity to UVB-resistance protein UVR8 [Arabidopsis thaliana] GI:5478530; contains Pfam profile PF00415: Regulator of chromosome condensation (RCC1) | At5g60870 | 56.2262779 | 71.086 | 60.7978757 | 84.985 | 12.5590816 | 53.947 | 0.579720264 | 0.00016 |
| 4404 | 7 | lectin protein kinase family protein contains Pfam domains, PF00138: Legume lectins alpha domain, PF00139: Legume lectins beta domain and PF00069: Protein kinase domain | At5g03140 | 75.5512867 | 100.48 | 64.4498591 | 94.557 | 90.455904 | 192.138 | 0.634762607 | 0.00016 |
| 5249 | 9 | actin-related protein 4 (ARP4) neary identical to actin-related protein 4 (ARP4) [Arabidopsis thaliana] GI:21427463; contains Pfam profile PF00022: Actin; supporting cDNA gi\|21427462\|gb\|AF507912.1\| | At1g18450 | 211.737623 | 239.18 | 53.5162159 | 89.589 | 37.2174394 | 99.917 | 0.618366946 | 0.000142 |
| 7043 | 11 | harpin-induced family protein / HIN1 family protein / harpin-responsive family protein similar to harpin-induced protein hin1 ( GI:1619321) [Nicotiana tabacum] | At5g22200 | 34.0005925 | 65.473 | 28.6964316 | 47.536 | 37.9349834 | 53.296 | 0.611588048 | 0.00013 |
| 10352 | 16 | expressed protein | At5g56250 | 29.7971785 | 57.733 | 39.9248466 | 74.238 | 29.4667272 | 64.974 | 0.502477116 | 0.000126 |
| 5273 | 9 | acetyltransferase-related low similarity to O-acetyltransferase [Cryptococcus neoformans var. neoformans] GI:17063556 | At1g29890 | 158.145698 | 231.217 | 43.8317677 | 60.066 | 54.4096283 | 97.708 | 0.656852387 | 0.000126 |
| 23003 | 36 | basic helix-loop-helix (bHLH) family protein contains Pfam profile: PF00010 helix-loop-helix DNA-binding domain | At2g41130 | 70.8612179 | 110.931 | 64.714357 | 150.526 | 14.208608 | 39.935 | 0.474833751 | 0.00012 |
| 6506 | 11 | D-3-phosphoglycerate dehydrogenase / 3-PGDH identical to SP\|O04130 | At1g17745 | 48.9382211 | 57.754 | 33.6772781 | 64.38 | 19.656169 | 33.029 | 0.655192149 | 0.000114 |
| 27740 | 43 | pentatricopeptide (PPR) repeat-containing protein contains Pfam profile PF01535: PPR repeat | At4g20090 | 29.8923703 | 62.525 | 39.9583072 | 59.227 | 187.80889 | 229.468 | 0.657067968 | 0.000114 |
| 19519 | 31 | ribonuclease P family protein / Rpp14 family protein contains Pfam profile: PF01900 Rpp14 family | At1g04635 | 91.6900435 | 110.938 | 43.6596848 | 116.766 | 39.2051187 | 68.195 | 0.591767617 | 0.000112 |
| 30349 | 47 | DNA-binding protein-related contains Pfam domain PF03479: Domain of unknown function (DUF296), found in AT-hook motifs Pfam:PF02178 | At4g25320 | 109.070572 | 194.664 | 53.538523 | 67.863 | 21.9139582 | 69.541 | 0.554781742 | 0.000111 |
| 1623 | 3 | expressed protein | At3g22415 | 65.2224396 | 98.927 | 37.7499088 | 87.185 | 24.2934001 | 114.724 | 0.43468004 | 0.000106 |
| 11940 | 19 | expressed protein contains Pfam profile: PF05097 protein of unknown function (DUF688) | At2g34170 | 73.1800478 | 88.422 | 41.2776101 | 97.169 | 23.9098852 | 84.845 | 0.511410509 | 0.000105 |
| 6514 | 11 | glycoside hydrolase family 28 protein / polygalacturonase (pectinase) family protein similar to polygalacturonase PG1 GI:5669846, PG2 GI:5669848 from (Glycine max); contains PF00295: Glycosyl hydrolases family 28 (polygalacturonases) | At1g02460 | 38.172989 | 48.435 | 63.2245645 | 96.482 | 20.2025746 | 52.579 | 0.609220008 | 0.000104 |
| 20009 | 31 | expressed protein similar to unknown protein (sp\|Q9ZE28) | At3g06180 | 55.632666 | 80.142 | 69.860913 | 89.787 | 13.1632207 | 61.169 | 0.562481427 | 0.000102 |
| 13899 | 22 | apurinic endonuclease-redox protein / DNA-(apurinic or apyrimidinic site) lyase identical to apurinic endonuclease-redox protein SP: P45951 from [Arabidopsis thaliana] | At2g41460 | 70.1210748 | 103.539 | 65.4664234 | 83.227 | 25.4109542 | 99.42 | 0.573145291 | 0.0001 |
| 6581 | 11 | protein kinase family protein contains Pfam domain, PF00069: Protein kinase domain | At1g33770 | 26.5863263 | 56.038 | 32.8710374 | 70.314 | 13.1920874 | 51.652 | 0.39910878 | 0.000099 |
| 30888 | 48 | hypothetical protein | At3g32394 | 51.5779223 | 97.756 | 50.2896599 | 102.97 | 15.8931869 | 35.019 | 0.489951683 | 0.000084 |
| 12078 | 19 | leucine-rich repeat transmembrane protein kinase, putative several receptor-like protein kinases | At3g57830 | 77.9781433 | 114.894 | 33.231137 | 85.009 | 19.7798834 | 50.622 | 0.486782152 | 0.00008 |
| 15392 | 24 | clathrin adaptor complexes medium subunit family protein contains Pfam profile: PF00928 adaptor complexes medium subunit family | At4g24550 | 65.2716398 | 99.627 | 114.591336 | 139.145 | 38.2370358 | 79.929 | 0.652779345 | 0.00008 |
| 28377 | 44 | farnesyl pyrophosphate synthetase 2 (FPS2) / FPP synthetase 2 / farnesyl diphosphate synthase 2 identical to SP\|Q43315 Farnesyl pyrophosphate synthetase 2 (FPP synthetase 2) (FPS 2) (Farnesyl diphosphate synthetase 2) [Includes: Dimethylallyltransferase (EC 2.5.1.1); Geranyltranstransferase (EC 2.5.1.10)] (Aral | At4g17190 | 73.4453014 | 94.746 | 49.8514856 | 80.395 | 16.6828976 | 68.491 | 0.546280298 | 0.000078 |
| 5870 | 10 | VHS domain-containing protein / GAT domain-containing protein weak similarity to SP\|Q9UJY5 ADP-ribosylation factor binding protein GGA1 {Homo sapiens}; contains Pfam profiles PF00790: VHS domain, PF03127: GAT domain | At1g06210 | 62.9153749 | 76.81 | 41.4241993 | 47.711 | 5.68674226 | 57.454 | 0.595438212 | 0.000076 |
| 12000 | 19 | protein kinase, putative similar to viroid symptom modulation protein [Lycopersicon esculentum] gi\|7672777\|gb\|AAF66637 | At3g12690 | 47.3862736 | 67.24 | 67.2127471 | 80.6 | 29.4131176 | 70.331 | 0.625528316 | 0.000075 |
| 13443 | 21 | hypothetical protein | At4g22217 | 29.683804 | 75.308 | 82.1648467 | 96.119 | 11.4683323 | 52.967 | 0.488502661 | 0.000074 |
| 3008 | 5 | Ulp1 protease family protein contains Pfam profile PF02902: Ulp1 protease family, C-terminal catalytic domain; similar to At1g32840, At2g06430, At2g15140, At2g04980, At2g14130, At3g44500, At2g15190, At3g47260, At5g34900, At3g29210, At2g02210, At3g32900 | At4g04010 | 50.1746452 | 69.215 | 35.2403652 | 53.166 | 46.4176729 | 89.105 | 0.636226265 | 0.000072 |
| 8800 | 14 | calcineurin-like phosphoesterase family protein contains Pfam profile: PF00149 calcineurin-like phosphoesterase | At3g46120 | 90.5691331 | 141.307 | 69.4386724 | 90.957 | 56.3498837 | 101.341 | 0.653468047 | 0.000068 |
| 19997 | 31 | expressed protein | At5g02440 | 63.8619453 | 84.574 | 55.1701246 | 121.273 | 31.376054 | 92.771 | 0.516078719 | 0.000066 |
| 6666 | 11 | eukaryotic translation initiation factor 2 family protein / eIF-2 family protein similar to SP\|O60841 Translation initiation factor IF-2 {Homo sapiens}; contains Pfam profile PF00009: Elongation factor Tu GTP binding domain | At1g76825 | 28.7607642 | 59.523 | 15.3823071 | 62.245 | 31.1904823 | 52.847 | 0.440172018 | 0.000063 |
| 28485 | 44 | speckle-type POZ protein-related contains Pfam PF00651 : BTB/POZ domain; contains Pfam PF00917: MATH domain; similar to Speckle-type POZ protein (SP:O43791) [Homo sapiens] | At5g19000 | 148.907814 | 213.247 | 63.866689 | 91.439 | 12.8992964 | 31.552 | 0.601858931 | 0.000059 |
| 3236 | 5 | disease resistance protein (CC-NBS-LRR class), putative domain signature CC-NBS-LRR exists, suggestive of a disease resistance protein. | At5g66910 | 68.2396994 | 89.164 | 64.4004649 | 74.647 | 14.8560471 | 47.941 | 0.64598109 | 0.000056 |
| 5816 | 9 | peroxidase, putative similar to peroxidase [Nicotiana tabacum] gi\|5381253\|dbj\|BAA82306 | At5g58390 | 68.2450472 | 105.839 | 75.7611288 | 86.533 | 19.0437823 | 84.081 | 0.582270348 | 0.000054 |
| 7065 | 11 | hypothetical protein | At5g38050 | 33.2946757 | 52.715 | 14.6637013 | 48.255 | 28.4234018 | 91.187 | 0.415727237 | 0.000052 |
| 21539 | 34 | F-box family protein similar to hypothetical protein GB:AAD22295 GI:4544385 from [Arabidopsis thaliana] | At1g47300 | 37.8403524 | 56.732 | 67.7019089 | 78.547 | 17.7303469 | 50.378 | 0.626958925 | 0.000052 |
| 1580 | 3 | no apical meristem (NAM) family protein similar to NAC2 (GI:6456751) {Arabidopsis thaliana} | At3g03200 | 43.0705546 | 78.03 | 21.578888 | 149.716 | 26.938828 | 36.168 | 0.480310457 | 0.000048 |
| 7394 | 12 | DC1 domain-containing protein contains Pfam profile PF03107: DC1 domain | At2g37800 | 42.4095597 | 50.517 | 54.1806474 | 80.871 | 21.6789007 | 57.295 | 0.62928262 | 0.000048 |
| 2554 | 4 | expressed protein | At5g55610 | 37.9868836 | 50.179 | 48.6405312 | 79.115 | 6.8558441 | 58.253 | 0.496508767 | 0.000046 |
| 21659 | 34 | expressed protein and genefinder | At2g24030 | 29.8677702 | 38.37 | 76.9577429 | 96.448 | 3.66194861 | 45.053 | 0.552538362 | 0.000044 |
| 20147 | 31 | galactosyltransferase family protein contains Pfam profile: PF01762 galactosyltransferase | At1g33430 | 69.0365298 | 97.206 | 68.496996 | 105.096 | 5.70323753 | 34.265 | 0.509469988 | 0.00004 |
| 21647 | 34 | DC1 domain-containing protein contains Pfam profile PF03107: DC1 domain | At2g17590 | 57.0006473 | 69.097 | 45.7963819 | 84.233 | 30.4935574 | 65.176 | 0.6121628 | 0.00004 |
| 28432 | 44 | expressed protein | At4g36630 | 83.5142428 | 114.569 | 79.8831537 | 102.078 | 23.6047228 | 62.747 | 0.629233787 | 0.000037 |
| 7093 | 11 | expressed protein contains similarity to unknown protein (pir\|\|T26512) | At5g51130 | 20.2148919 | 55.607 | 22.1094772 | 57.815 | 25.3965208 | 57.587 | 0.395653481 | 0.000036 |
| 7024 | 11 | armadillo/beta-catenin repeat family protein contains Pfam profile: PF00514 armadillo/beta-catenin-like repeat | At5g14510 | 35.3118866 | 79.926 | 32.1556183 | 56.547 | 30.2873666 | 62.209 | 0.499108304 | 0.000036 |
| 1445 | 3 | CCAAT-binding transcription factor (CBF-B/NF-YA) family protein contains Pfam profile: PF02045 CCAAT-binding transcription factor (CBF-B/NF-YA) subunit B | At1g72830 | 53.6806989 | 60.918 | 31.3254773 | 69.174 | 20.7242373 | 56.772 | 0.566363251 | 0.000035 |
| 15491 | 24 | hypothetical protein | At5g23640 | 33.819835 | 62.192 | 1990.88704 | 3319.302 | 34.4111826 | 65.936 | 0.555158564 | 0.000034 |
| 1787 | 3 | hypothetical protein | At4g36460 | 71.0409058 | 100.365 | 39.0341578 | 68.698 | 40.990731 | 96.351 | 0.567152042 | 0.000034 |
| 4013 | 7 | transcription factor, putative similar to Transcription factor GB:CAA74053 GI:2398533 from [Arabidopsis thaliana] similarity to transcription factor Hap5a similar to transcription factor Hap5a [Arabidopsis thaliana](GI:6523090) | At1g56170 | 63.0105668 | 91.284 | 79.0482326 | 117.074 | 48.7352575 | 89.471 | 0.636724315 | 0.000032 |
| 6917 | 11 | cytochrome P450 71A19, putative (CYP71A19) Identical to Cytochrome P450 (SP:Q9T0K0) [Arabidopsis thaliana]; similar to cytochrome P450LIA1, Persea americana, M32885 | At4g13290 | 28.6163721 | 54.136 | 23.2949378 | 50.535 | 21.6809626 | 55.703 | 0.459597422 | 0.00003 |
| 11828 | 19 | hypothetical protein | At1g63700 | 66.6353429 | 77.296 | 40.848996 | 84.63 | 18.1365428 | 44.866 | 0.582998532 | 0.00003 |
| 23184 | 36 | transcription initiation factor-related contains weak similarity to Transcription initiation factor IIE, beta subunit (TFIIE-beta) (Swiss-Prot:P29084) [Homo sapiens] | At4g20330 | 68.3038736 | 98.815 | 104.605743 | 127.26 | 20.3840225 | 64.992 | 0.608951074 | 0.00003 |
| 14218 | 22 | protein kinase, putative similar to stpk1 protein kinase [Solanum tuberosum] gi\|1200256\|emb\|CAA62476 | At5g40030 | 41.780652 | 68 | 61.8494939 | 97.833 | 47.4816174 | 77.835 | 0.618881693 | 0.00003 |
| 27963 | 44 | protease inhibitor/seed storage/lipid transfer protein (LTP) family protein contains Pfam profile PF00234:Protease inhibitor/seed storage/LTP family; low similarity to SP:Q42978 Nonspecific lipid-transfer protein 2 precursor (LTP 2) {Oryza sativa} | At1g03103 | 58.4135506 | 97.851 | 66.2057429 | 115.296 | 20.4953655 | 47.511 | 0.534189953 | 0.000028 |
| 8706 | 14 | DNA polymerase delta small subunit-related similar to DNA polymerase delta small subunit SP:Q9LRE5 from [Oryza sativa] | At2g42120 | 52.9523211 | 86.996 | 73.7311869 | 113.415 | 18.7881057 | 95.422 | 0.4852237 | 0.000024 |
| 14314 | 23 | speckle-type POZ protein-related contains Pfam profile:PF00651 BTB/POZ domain; similar to Speckle-type POZ protein (SP:O43791) [Homo sapiens] | At1g01640 | 49.636651 | 81.298 | 70.0154691 | 111.624 | 21.4376575 | 86.664 | 0.495053664 | 0.000024 |
| 28459 | 44 | myosin heavy chain-related contains weak similarity to Myosin heavy chain, cardiac muscle alpha isoform (MyHC-alpha) (Alpha isomyosin) (Fragment) (Swiss-Prot:P04460) [Oryctolagus cuniculus] | At5g07890 | 166.679805 | 239.104 | 27.2640001 | 113.019 | 11.7858661 | 43.484 | 0.403124874 | 0.000024 |
| 9941 | 16 | hypothetical protein | At2g15045 | 31.5427184 | 52.634 | 37.9777594 | 48.781 | 15.5570959 | 46.416 | 0.570995546 | 0.000024 |
| 28169 | 44 | expressed protein | At2g27790 | 53.9555787 | 96.655 | 69.3096101 | 79.392 | 12.7858915 | 39.715 | 0.584391538 | 0.000024 |
| 12019 | 19 | dynein light chain, putative similar to SP\|O02414 Dynein light chain LC6, flagellar outer arm {Anthocidaris crassispina}; contains Pfam profile PF01221: Dynein light chain type 1 | At3g16120 | 56.4434008 | 86.858 | 27.6878341 | 46.139 | 29.5327083 | 71.635 | 0.554065981 | 0.000023 |
| 6879 | 11 | DNAJ heat shock N-terminal domain-containing protein contains Pfam profile PF00226 DnaJ domain | At3g58020 | 21.3229675 | 44.961 | 19.4820249 | 37.336 | 16.5715546 | 51.966 | 0.438316558 | 0.000022 |
| 27468 | 43 | glycine-rich protein | At1g76965 | 46.7648528 | 63.106 | 57.3323155 | 65.349 | 18.7963533 | 60.77 | 0.642560209 | 0.000021 |
| 21582 | 34 | protein kinase-related | At1g66940 | 14.9130285 | 57.093 | 57.0550707 | 64.892 | 4.68053116 | 42.133 | 0.41717551 | 0.00002 |
| 27718 | 43 | cytochrome P450 71A20, putative (CYP71A20) Identical to Cytochrome P450 (SP:Q9T0K2) [Arabidopsis thaliana]; similar to cytochrome P450 71A4, Solanum melongena, PIR2:S36805 | At4g13310 | 57.8488171 | 64.259 | 52.8246972 | 82.464 | 1.80004568 | 61.238 | 0.523405912 | 0.00002 |
| 7156 | 12 | D-3-phosphoglycerate dehydrogenase / 3-PGDH identical to SP\|O04130 | At1g17745 | 30.3982774 | 50.494 | 58.9639172 | 86.932 | 33.887458 | 79.568 | 0.568728994 | 0.00002 |
| 25808 | 40 | expressed protein | At4g31020 | 40.5303235 | 57.693 | 83.3471205 | 92.851 | 13.6993168 | 40.034 | 0.627450997 | 0.00002 |
| 6870 | 11 | expressed protein DNA-binding Mel-18 protein, Homo sapiens, PIR:JN0717 | At3g54360 | 21.6759259 | 80.303 | 32.0743569 | 61.123 | 19.0912062 | 32.961 | 0.457961222 | 0.000016 |
| 6270 | 10 | expressed protein | At4g14200 | 30.2774159 | 55.899 | 36.3238507 | 43.555 | 9.44766246 | 39.809 | 0.537648807 | 0.000016 |
| 15394 | 24 | protein kinase (AFC2) identical to protein kinase AFC2 [Arabidopsis thaliana] SWISS-PROT:P51567 | At4g24740 | 48.1285559 | 72.714 | 91.1625563 | 104.214 | 17.9324139 | 86.881 | 0.581017835 | 0.000015 |
| 30772 | 48 | zinc finger (C3HC4-type RING finger) family protein contains Pfam profile: PF00097 zinc finger, C3HC4 type (RING finger) | At2g28920 | 61.8907259 | 81.12 | 59.5072533 | 76.967 | 11.631223 | 48.746 | 0.591571442 | 0.000015 |
| 28319 | 44 | expressed protein | At3g55070 | 30.8228971 | 55.103 | 57.2112201 | 73.586 | 123.469113 | 210.712 | 0.640934828 | 0.000015 |
| 31041 | 48 | VHS domain-containing protein / GAT domain-containing protein weak similarity to Hrs [Rattus norvegicus] GI:8547026; contains Pfam profiles PF00790: VHS domain, PF03127: GAT domain | At5g01760 | 36.622111 | 56.014 | 48.7504732 | 58.063 | 36.7452625 | 154.919 | 0.576868795 | 0.000014 |
| 12097 | 19 | myb family transcription factor contains PFAM profile: PF00249 myb-like DNA binding domain | At4g01280 | 36.6830766 | 68.187 | 39.4611785 | 71.556 | 28.9512502 | 57.088 | 0.532194657 | 0.000012 |
| 27492 | 43 | expressed protein | At2g11360 | 41.0116304 | 53.42 | 58.2086641 | 92.405 | 23.8500898 | 65.085 | 0.588031865 | 0.000012 |
| 14526 | 23 | glutathione S-transferase, putative | At2g29440 | 61.3580796 | 69.429 | 40.6307055 | 60.135 | 26.8192374 | 121.705 | 0.593257915 | 0.000012 |
| 5607 | 9 | callose synthase, putative / 1,3-beta-glucan synthase, putative similar to callose synthase 1 catalytic subunit GI:13649388 from [Arabidopsis thaliana] | At4g04970 | 75.1544759 | 99.908 | 37.6925478 | 66.101 | 36.7308291 | 72.776 | 0.60905805 | 0.000012 |
| 14817 | 23 | cytochrome P450 family protein | At5g09970 | 63.4940128 | 120.771 | 86.7903737 | 103.863 | 19.6211165 | 36.682 | 0.632086725 | 0.000012 |
| 7403 | 12 | expressed protein an isoform contains a GA donor splice site supported by FL-cDNA alignment which truncates the ORF. | At2g41150 | 45.8503696 | 54.492 | 33.2120167 | 40.673 | 28.7141308 | 90.097 | 0.658892956 | 0.000012 |
| 6533 | 11 | pentatricopeptide (PPR) repeat-containing protein contains Pfam profile PF01535: PPR repeat | At1g11710 | 43.4267217 | 56.344 | 23.8510208 | 69.346 | 20.2932985 | 44.643 | 0.523084439 | 0.000011 |
| 15079 | 24 | peptidase M3 family protein / thimet oligopeptidase family protein similar to SP\|P42676 Neurolysin, mitochondrial precursor (EC 3.4.24.16) (Mitochondrial oligopeptidase M) {Rattus norvegicus}; contains Pfam profile PF01432: Peptidase family M3 | At1g67690 | 36.3258399 | 74.499 | 92.8531123 | 115.914 | 38.5453082 | 65.685 | 0.625158005 | 0.00001 |
| 6806 | 11 | expressed protein contains Pfam profile: PF03398 eukaryotic protein of unknown function, DUF292 | At3g15490 | 28.3596751 | 59.858 | 37.0328964 | 72.845 | 23.8129755 | 44.62 | 0.50528194 | 0.000009 |
| 1312 | 3 | no apical meristem (NAM) family protein contains Pfam PF02365 : No apical meristem (NAM) protein; similar to NAC1 (GI:21554126) (Arabidopsis thaliana) | At1g02250 | 25.2055102 | 37.128 | 40.1017096 | 68.726 | 20.9572329 | 40.275 | 0.594245375 | 0.000009 |
| 28049 | 44 | cinnamyl-alcohol dehydrogenase, putative (CAD) similar to GB:X88797 from [Eucalyptus gunnii] (Plant Mol. Biol. 36 (5), 755-765 (1998)) | At1g51410 | 65.7326249 | 77.332 | 53.481162 | 75.151 | 15.3096669 | 44.146 | 0.63615037 | 0.000009 |
| 30399 | 47 | heavy-metal-associated domain-containing protein low similarity to gi:3168840 copper homeostasis factor; contains Pfam heavy-metal-associated domain PF00403; predicted proteins, Arabidopsis thaliana | At5g02600 | 52.5095187 | 65.338 | 21.3319171 | 44.649 | 6.11149531 | 43.794 | 0.473659964 | 0.000008 |
| 17580 | 28 | kinase interacting family protein similar to kinase interacting protein 1 (GI:13936326) [Petunia integrifolia] | At1g09720 | 36.2509699 | 47.976 | 53.4110541 | 64.959 | 10.3425305 | 46.365 | 0.600300377 | 0.000008 |
| 30084 | 47 | expressed protein contains Pfam profile: PF04765 protein of unknown function (DUF616) | At2g02910 | 44.4952232 | 64.056 | 37.1603652 | 47.957 | 17.5695181 | 45.238 | 0.619292709 | 0.000006 |
| 15411 | 24 | expressed protein | At4g31430 | 26.3745513 | 57.974 | 99.5978094 | 123.652 | 41.6773464 | 94.595 | 0.566997811 | 0.000005 |
| 23325 | 36 | transcriptional factor B3 family protein contains Pfam profile PF02362: B3 DNA binding domain | At5g42700 | 25.5424251 | 86.689 | 45.1781578 | 106.632 | 25.2088872 | 33.778 | 0.488212706 | 0.000004 |
| 23071 | 36 | elongation factor Tu family protein similar to eukaryotic translation elongation factor 2 GB:NP_001952 [Homo sapiens] | At3g22980 | 63.1239413 | 83.778 | 35.2594856 | 54.204 | 45.4341428 | 111.121 | 0.6042779 | 0.000004 |
| 9029 | 14 | expressed protein contains similarity to carboxyl-terminal proteinase contains Pfam profile PF03080: Arabidopsis proteins of unknown function; expression supported by MPSS | At5g46200 | 48.6654805 | 77.919 | 88.7613612 | 99.005 | 18.7283104 | 63.298 | 0.60565812 | 0.000004 |
| 30345 | 47 | protease inhibitor/seed storage/lipid transfer protein (LTP) family protein similar to hydroxyproline-rich glycoprotein DZ-HRGP from Volvox carteri f. nagariensis GP\|6523547; contains Pfam profile PF00234 Protease inhibitor/seed storage/LTP family | At4g22470 | 30.9234368 | 72.897 | 44.3735105 | 61.764 | 38.5803606 | 49.609 | 0.640110825 | 0.000004 |
| 6963 | 11 | aspartyl protease family protein contains Pfam domain, PF00026: eukaryotic aspartyl protease | At4g30040 | 22.7668883 | 32.211 | 12.3342075 | 59.307 | 11.2827606 | 50.907 | 0.378803909 | 0.000003 |
| 6597 | 11 | protein kinase family protein contains protein kinase domain, Pfam:PF00069 | At1g50230 | 26.7574577 | 33.395 | 18.1515685 | 77.1 | 18.7448056 | 50.334 | 0.469692921 | 0.000003 |
| 14089 | 22 | calmodulin-binding family protein contains IQ calmodulin-binding motif, Pfam:PF00612 | At4g21820 | 28.7971296 | 82.08 | 15.916083 | 67.832 | 28.2048395 | 69.847 | 0.329763618 | 0.000002 |
| 6904 | 11 | calcium-dependent protein kinase, putative / CDPK, putative similar to calcium-dependent protein kinase [Nicotiana tabacum] gi\|3283996\|gb\|AAC25423; contains protein kinase domain, Pfam:PF00069; contains EF hand domain (calcium-binding EF-hand), Pfam:PF00036, INTERPRO:IPR002048 | At4g04710 | 15.7408764 | 36.985 | 20.6515519 | 52.02 | 13.5632308 | 43.829 | 0.377350695 | 0.000002 |
| 7391 | 12 | origin recognition complex subunit 2 (ORC2) identical to origin recognition complex subunit 2 (ORC2) Sp:Q38899 from [Arabidopsis thaliana] | At2g37560 | 37.2550743 | 46.784 | 31.9118341 | 73.675 | 20.5139227 | 76.336 | 0.499430776 | 0.000002 |
| 14984 | 24 | serine/threonine protein phosphatase 2A (PP2A) regulatory subunit B', putative similar to SWISS-PROT:Q28653 serine/threonine protein phosphatase 2A, 56 kDa regulatory subunit, delta isoform (PP2A, B subunit, B' delta isoform, PP2A, B subunit, B56 delta isoform, PP2A, B subunit, PR61 delta isoform, PP2A, B subu | At1g13460 | 14.1172676 | 72.421 | 53.7647802 | 71.672 | 53.9642562 | 95.425 | 0.503532873 | 0.000002 |
| 7224 | 12 | F-box family protein contains F-box domain Pfam:PF00646 | At1g30930 | 28.8473994 | 52.17 | 35.3742076 | 50.056 | 16.4684592 | 46.671 | 0.537501801 | 0.000002 |
| 3428 | 6 | hypothetical protein | At2g05087 | 41.3335713 | 51.5 | 34.4946723 | 70.524 | 11.9570045 | 36.358 | 0.540193946 | 0.000002 |
| 20024 | 31 | expressed protein | At5g13590 | 59.6114701 | 72.976 | 36.4608798 | 84.481 | 28.5553639 | 57.13 | 0.582760705 | 0.000002 |
| 6635 | 11 | cox19 family protein contains Pfam profile PF05490: Cox19 protein | At1g66590 | 14.0423977 | 27.973 | 5.09238176 | 23.479 | 2.42892762 | 25.564 | 0.271300928 | 0.000001 |
| 6632 | 11 | subtilase family protein contains similarity to subtilase; SP1 GI:9957714 from [Oryza sativa] | At1g66210 | 9.19724103 | 40.53 | 19.0773113 | 44.152 | 12.2745383 | 45.891 | 0.308826156 | 0.000001 |
| 6810 | 11 | hypothetical protein | At3g15910 | 9.12344063 | 25.059 | 9.90911206 | 41.518 | 23.7696754 | 50.756 | 0.357020429 | 0.000001 |
| 6603 | 11 | expressed protein ; expression supported by MPSS | At1g51000 | 9.00257911 | 30.864 | 20.7216598 | 64.187 | 2.06396991 | 4.4 | 0.361200719 | 0.000001 |

| | | | | | | | | | | | | |
|---|---|---|---|---|---|---|---|---|---|---|---|---|
| 12055 | 19 | expressed protein | At3g47720 | 15.5975539 | 32.185 | 23.4463071 | 71.655 | 18.9674917 | 60.802 | | 0.374595981 | 0.000001 |
| 15696 | 25 | F-box protein-related contains weak hit to Pfam PF00646: F-box domain; contains weak hit to TIGRFAM TIGR01640 : F-box protein interaction domain | At1g46984 | 13.8691272 | 58.055 | 14.0231702 | 39.462 | 16.8829027 | 29.042 | | 0.39186077 | 0.000001 |
| 24163 | 38 | SWIM zinc finger family protein contains Pfam domain PF04434: SWIM zinc finger | At1g60560 | 30.9555239 | 59.674 | 47.8645644 | 70.278 | 8.46413234 | 47.269 | | 0.45962721 | 0.000001 |
| 19868 | 31 | AAA-type ATPase family protein contains Pfam profile: ATPase family PF00004 | At3g50930 | 37.7002386 | 55.134 | 36.1995686 | 76.83 | 11.5961706 | 42.261 | | 0.476450517 | 0.000001 |
| 24579 | 38 | heavy-metal-associated domain-containing protein contains Pfam heavy-metal-associated domain PF00403; glycine-rich protein GRP22, rape, PIR:S31415; isoform contains a non-consensus TG-acceptor splice site at intron 3 | At5g19090 | 22.777584 | 40.335 | 45.6545727 | 56.602 | 7.50328321 | 42.525 | | 0.515914531 | 0.000001 |
| 22198 | 35 | C2 domain-containing protein low similarity to CLB1 [Lycopersicon esculentum] GI:2789434; contains Pfam profile PF00168: C2 domain | At1g50260 | 39.0404111 | 54.587 | 31.7604648 | 64.137 | 16.6767119 | 47.494 | | 0.520508795 | 0.000001 |
| 28287 | 44 | myosin heavy chain-related similar to Myosin heavy chain, non-muscle (Zipper protein) (Myosin II)(SP:Q99323) {Drosophila melanogaster} | At3g30230 | 24.7295511 | 53.068 | 69.0690126 | 122.056 | 8.12185561 | 12.918 | | 0.55353368 | 0.000001 |
| 20290 | 32 | expressed protein contains Pfam profile: PF04842 plant protein of unknown function (DUF639) | At1g71240 | 35.6990713 | 58.498 | 31.0609793 | 68.931 | 25.1800205 | 41.874 | | 0.55406647 | 0.000001 |
| 6846 | 11 | hypothetical protein | At3g43950 | 18.8693716 | 21.306 | 4.16026557 | 32.622 | 6.78161541 | 10.436 | | 0.554331652 | 0.000001 |
| 3173 | 5 | nitrate-responsive NOI protein, putative similar to nitrate-induced NOI protein [Zea mays] GI:2642213 | At5g40645 | 44.1829084 | 49.373 | 19.8150374 | 43.344 | 14.1611841 | 43.452 | | 0.559313869 | 0.000001 |
| 7240 | 12 | endonuclease/exonuclease/phosphatase family protein similar to SP\|P32019 Type II inositol-1,4,5-trisphosphate 5-phosphatase precursor (EC 3.1.3.56) {Homo sapiens}; contains Pfam profile PF03372: Endonuclease/Exonuclease/phosphatase family | At1g47510 | 31.7833718 | 52.203 | 47.9872532 | 64.656 | 26.0130313 | 73.073 | | 0.569007374 | 0.000001 |
| 21813 | 34 | serine carboxypeptidase S10 family protein similar to SP\|P52711 Serine carboxypeptidase II-3 precursor (EC 3.4.16.6) Hordeum vulgare; contains Pfam profile PF0450 serine carboxypeptidase | At3g52020 | 25.0889269 | 38.353 | 22.0505229 | 79.81 | 17.7468422 | 22.203 | | 0.576581715 | 0.000001 |
| 5047 | 8 | expressed protein | At5g05020 | 39.9923293 | 45.788 | 40.2674192 | 67.749 | 27.7862722 | 63.65 | | 0.634777835 | 0.000001 |
| 15081 | 24 | hypothetical protein hypothetical protein | At1g67855 | 28.4484495 | 56.915 | 56.3157512 | 62.594 | 22.2871636 | 39.1 | | 0.656514679 | 0.000001 |
| 23131 | 36 | DC1 domain-containing protein contains Pfam profile PF03107: DC1 domain | At3g59130 | 51.2335205 | 63.632 | 41.1469545 | 48.072 | 20.1819555 | 64.2 | | 0.658486123 | 0.000001 |
